# Awakening the HSC: Dynamic Modeling of HSC Maintenance Unravels Regulation of the TP53 Pathway and Quiescence

**DOI:** 10.3389/fphys.2020.00848

**Published:** 2020-07-31

**Authors:** Nensi Ikonomi, Silke D. Kühlwein, Julian D. Schwab, Hans A. Kestler

**Affiliations:** Institute of Medical Systems Biology, Ulm University, Ulm, Germany

**Keywords:** hematopoietic stem cell, Boolean network, dynamic modeling, TP53, stem cell awakening, maintenance of quiescence, niche interactions, homeostasis

## Abstract

Hematopoietic stem cells (HSCs) provide all types of blood cells during the entire life of the organism. HSCs are mainly quiescent and can eventually enter the cell cycle to differentiate. HSCs are maintained and tightly regulated in a particular environment. The stem cell niche regulates dormancy and awakening. Deregulations of this interplay can lead to hematopoietic failure and diseases. In this paper, we present a Boolean network model that recapitulates HSC regulation in virtue of external signals coming from the niche. This Boolean network integrates and summarizes the current knowledge of HSC regulation and is based on extensive literature research. Furthermore, dynamic simulations suggest a novel systemic regulation of TP53 in homeostasis. Thereby, our model indicates that TP53 activity is balanced depending on external stimulations, engaging a regulatory mechanism involving ROS regulators and RAS activated transcription factors. Finally, we investigated different mouse models and compared them to *in silico* knockout simulations. Here, the model could recapitulate *in vivo* observed behaviors and thus sustains our results.

## 1. Introduction

The hematopoietic system is composed of heterogenic populations. These populations comprise highly specialized cells with unique and peculiar functions. All these different blood cells, approximately 4 − 5 · 10^11^ in total, are produced hierarchically from a population of cells called hematopoietic stem cells (HSCs) (Kaushansky, 2006; Jagannathan-Bogdan and Zon, 2013). Nevertheless, recent results coming from single-cell RNA sequencing suggest that hematopoiesis is not necessarily hierarchical, but can also be radial (Macaulay et al., 2016; Athanasiadis et al., 2017; Laurenti and Göttgens, 2018; Zhang et al., 2018; Yokota, 2019).

HSCs are a particular population of cells characterized by the ability to self-renew and to generate differentiated blood cells (Pietras et al., 2011; Sun et al., 2014; Busch et al., 2015; Sawai et al., 2016). Under steady-state conditions, HSCs are maintained as a quiescent population of cells that can be activated in the presence of external differentiation stimuli (Fleming et al., 1993; Bradford et al., 1997; Cheshier et al., 1999; Passegué et al., 2005). This dormant state is believed to contribute to the long-term preservation of HSCs, mostly by minimizing replication and metabolic activity (Wilson et al., 2009; Warr et al., 2011; Bakker and Passegué, 2013). Fine-tuning of dormancy and the awakening mechanism of HSCs is of crucial relevance for correct hematopoiesis. In fact, not responsive dormant HSCs would lead to hematopoietic failure due to a lack of differentiated blood cells (Wilson et al., 2009). On the other hand, highly active HSCs would get to exhaustion of the population and lack of long-term maintenance of the hematopoietic system (Wilson et al., 2009).

Leading intrinsic players regulating HSC quiescence are polycomb ring finger protein (BMI1), TP53, reactive oxygen species (ROS) regulators and protein kinase B (AKT) signaling (Abbas et al., 2011; Pietras et al., 2011; Suda et al., 2011; Warr et al., 2011). Besides intrinsic factors, HSCs are regulated by the niche. The niche is defined as the specialized bone marrow environment where the HSCs reside (Schofield, 1978). Classically, two types of niches have been individuated for HSCs: the endosteal and the vascular niches. The endosteal niche is responsible for the maintenance of quiescence and is composed mainly of osteoblastic cells. In this context, hypoxic condition and signals promoting quiescence such as transforming growth factor beta (TGF-β) set HSCs in a dormant state (Zhang et al., 2003; Kopp et al., 2005; Sugiyama et al., 2006; Geiger et al., 2007; Jones and Wagers, 2008; Wagner et al., 2008; Guerrouahen et al., 2011). On the other hand, the vascular niche is characterized by endothelial and stromal cells and creates a milieu that supports proliferation, differentiation and trans-endothelial migration of HSCs (Zhang et al., 2003; Kopp et al., 2005; Sugiyama et al., 2006; Geiger et al., 2007; Jones and Wagers, 2008; Wagner et al., 2008; Guerrouahen et al., 2011). Here, a higher oxygen concentration and proliferating signals such as stem cell factor (SCF), fibroblast growth factor (FGF), and other growth factors are mostly present. Overall, these signals stimulate HSC activation and cycling (Coskun and Hirschi, 2010; Lilly et al., 2011). Moreover, HSCs and progenitors can be defined by surface markers as LSK (Lin^−^ Sca^+^ cKit^+^) population (Spangrude et al., 1988). Additionally, HSCs can be further divided into long-term HSCs (LT-HSC) and short-term HSCs (ST-HSC) depending again on marker selection. In this context, LT-HSCs are defined as Lin^−^ Sca^+^ cKit^+^CD150^+^CD48^+^ (Adolfsson et al., 2001; Kiel et al., 2005; Wilson and Trumpp, 2006; Challen et al., 2009; Foudi et al., 2009; Wilson et al., 2009). Phenotypically these two types of HSCs are distinguished based on their quiescence, long-term self-renewal ability, and engraftment. Hence, even the dormant state itself is regulated distinguishing between highly dormant and more active HSCs (Adolfsson et al., 2001; Kiel et al., 2005; Wilson and Trumpp, 2006; Challen et al., 2009; Foudi et al., 2009; Wilson et al., 2009).

Different models to unravel HSC fate commitment are available (Glauche et al., 2011; Krumsiek et al., 2011; Hamey et al., 2017; Olariu and Peterson, 2019). However, as it was underlined, HSC function does not only reside in its ability to differentiate. Unraveling mechanistic strategies by which HSCs are kept in quiescence and activeness is of crucial interest. Accordingly, the deregulation of HSC function is linked to cancerous conditions and aging (Warr et al., 2011; Bakker and Passegué, 2013). Even if a lot of effort has been implied in understanding the maintenance of HSCs, still many regulatory mechanisms are unknown. Crucial regulators of quiescence such as TP53, BMI1, ataxia-telangiectasia mutated (ATM) have been individuated. However, it is still not clear how they are differentially regulated in dormant (LT-HSC) and more active HSC (ST-HSC) (Warr et al., 2011; Bakker and Passegué, 2013). The regulation of these factors is often believed to be a result of crosstalk among different intrinsic and extrinsic pathways involved in HSC maintenance (Warr et al., 2011; Bakker and Passegué, 2013). Of interest in this context is the regulation of TP53.

TP53 is a ubiquitously expressed protein described to be activated in the context of DNA damage response and regulates cell survival (Toledo and Wahl, 2006; Vousden and Lane, 2007; Meek, 2009). In normal conditions, instead, TP53 is down-regulated by mouse double minute 2 homolog (MDM2) that causes its degradation (Jones et al., 1995; Montes de Oca Luna et al., 1995; Ringshausen et al., 2006). MDM2 expression is further sustained by TP53, causing negative feedback on its activity (Jones et al., 1995; Montes de Oca Luna et al., 1995; Ringshausen et al., 2006). Nevertheless, high levels of TP53 have been detected in HSCs. Its expression is linked to the maintenance of quiescence by induction of growth factor independent one transcriptional repressor (GFI1) (Forsberg et al., 2005; Liu Y. et al., 2009). How both intrinsic and extrinsic factors regulate TP53 in steady-state conditions is still an open question and a central point in further understanding of HSC maintenance (van Os et al., 2009; Pant et al., 2012).

To get new mechanistic insights about HSC maintenance and, in particular, on TP53 regulation, we constructed a Boolean network model describing quiescence and activation of HSCs.

Among dynamic modeling approaches, Boolean network models are used to describe the qualitative dynamic behavior of gene and protein regulatory networks (Kauffman, 1969; Hopfensitz et al., 2013). Boolean network models rely on the assumption of a switch-like behavior for each node (corresponding to a gene or a protein) that can be considered either active (1) or inactive (0) (Kauffman, 1969; Hopfensitz et al., 2013). Moreover, interactions are qualitatively described and summarized in Boolean functions, which do not require frequently unavailable kinetic parameters to be constructed. This peculiarity makes Boolean networks a powerful tool to describe large biological networks (Kauffman, 1969; Hopfensitz et al., 2013). Despite their simplicity, Boolean network models can faithfully represent the behavior of biological networks. In this context, they have been successfully applied to recapitulate different biological conditions such as cancer, aging, and senescence (Herrmann et al., 2012; Dahlhaus et al., 2016; Meyer et al., 2017; Siegle et al., 2018).

In the present work, we performed an extensive literature investigation to construct a Boolean network model recapitulating the regulation of HSC quiescence and cell cycle entry in the dependency on niche stimulation. Our aim is to unravel new mechanistic insights in the maintenance of hematopoietic stem cell quiescence, with particular attention to TP53 regulation. Strikingly, our model recapitulates the different statuses of the HSC, successfully describing dormant LT-HSC, activated ST-HSC, and cycling HSC as well as the progression from one to the other. Moreover, we could suggest a new general mechanism for TP53 regulation in homeostatic conditions. Finally, we tested our regulatory model by assessing its ability to recapitulate different knockout (K.O.) conditions. Here, the results support our model-based hypothesis of HSCs regulation.

## 2. Materials and Methods

### 2.1. Boolean Networks

Boolean networks are dynamic mathematical models to describe gene regulatory processes (Kauffman, 1969). These networks are defined as a set of *n* variables *X* = {*x*_1_, *x*_2_, …, *x*_*n*_}, *x*_*i*_ ∈ 𝔹 and a corresponding set of transition functions $F=\left\{f_{1},f_{2},\ldots .,f_{n}\right\},f_{i}:{𝔹}^{n}\to 𝔹$. Boolean functions integrate information about regulatory interactions coming from literature statements or other data sources. Here, regulatory dependencies are expressed using logical operators (Kauffman, 1969). Each compound *x*_*i*_ has its own Boolean function *f*_*i*_ which defines whether *x*_*i*_ is present or not. The state of the network at a specific point in time *t* is defined by a vector $\vec{x}\left(t\right)=\left(x_{1}\left(t\right),\ldots ,x_{n}\left(t\right)\right)$ containing the assignments of all compounds *x*_*i*_ within the network at this particular time step. Considering all possible combinations of assignments, this leads to a total number of 2^*n*^ possible states. Using synchronous updates, transitions from states at one point in time to their successors, $\vec{x}\left(t\right)\mapsto \vec{x}\left(t+1\right)$, are computed by updating all compounds at the same time. The transition of each compound is done by applying the corresponding Boolean function $x_{i}\left(t+1\right)=f_{i}\left(\vec{x}\left(t\right)\right),f_{i}:{𝔹}^{n}\to 𝔹$ (Kauffman, 1969). Due to the deterministic nature of the state space, with synchronous updating, the model eventually enters a recurrent sequence of states called attractor (Kauffman, 1969). Attractors denote the long-term behavior of a system, and in a biological context, they are often related to phenotypes (Kauffman, 1993; Thomas and Kaufman, 2001). All states leading to the same attractor are part of its so-called basin of attraction (Hopfensitz et al., 2013; Schwab et al., 2020).

### 2.2. Model Setup

For the construction of the Boolean network model describing the maintenance of HSC in their niche, we performed extensive literature research collecting published papers from NCBI and Google Scholar. We collected data in the context of hematopoietic stem cell regulation. Moreover, the influences of niche factors on intrinsic regulators were considered. In the present work, studies from mouse models as well as from human HSCs when available were used. For all components of the model, different levels of regulation have been considered. Detailed descriptions about all interactions and resulting Boolean network functions can be found in Table 1. The java framework ViSiBooL was used for modeling (Schwab et al., 2017).

**Table 1:** Boolean functions for the HSC model.

**Node**	**Boolean function**
External quiescence	External quiescence
External cycling	External cycling
PI3K	RAS
TSC1/2	¬AKT
mTORC1	¬TSC1/2
FOXO3A	External quiescence ∧¬AKT
ATM	FOXO3A
Mitochondria	mTORC1
ROS	Mitochondria ∨¬ATM ∨¬ FOXO3A ∨¬BMI1 ∨¬ TP53
Autophagy	FOXO3A ∧ ROS ∧¬ mTORC1
RAS	External cycling
ETS	RAS ∧¬ MEF
MEF	RAS
GSK3β	¬AKT
CTNNB1	¬GSK3β
cMYC	CTNNB1 ∧¬GSK3β
BMI1	cMYC ∨ (FOXO3A ∧ ATM)
MDM2	(TP53 ∨ MEF) ∧¬CDKN2D ∧¬ATM
TP53	¬MDM2
CDKN1C	External quiescence ∨ FOXO3A
CDKN1A	(TP53 ∨ FOXO3A ∨ External quiescence ∨ GFI1) ∧¬cMYC
CDKN1B	FOXO3A
GFI1	TP53
RB	¬CCND1 ∧¬CCNE1
E2F	¬RB ∧¬ GFI1
CCND1	¬CDKN2A ∧¬ CDKN1C ∧ cMYC
CCNE1	¬CDKN1C ∧ ((¬CDKN1A ∧¬CDKN1B) ∨ CCND1) ∧ E2F
S-phase	E2F ∧ CCNE1
AKT	PI3K
CDKN2A	(ETS ∨ ROS) ∧¬BMI1
CDKN2D	(E2F ∨ ROS) ∧¬BMI1
Pro-apoptotic proteins	ROS ∧ TP53 ∧¬AKT
Anti-apoptotic proteins	(RAS ∨ External quiescence) ∧¬GSK3β
CYCS	Pro-apoptotic proteins ∧¬Anti-apoptotic proteins
Apoptosis	CYCS ∧¬AKT
Senescence	(CDKN2A ∧ ROS) ∨ (TP53 ∧ ROS ∧ CDKN1A)

*Compounds are abbreviated according to accepted nomenclature. Regulatory interactions are summarized by logical connectives AND (∧), OR (∨), and NOT (¬). References for each Boolean function are provided in the Supplementary Section 1.1*.

### 2.3. Model Simulation

The analysis of the dynamic behavior of the HSC model was performed by synchronous updates with the R package BoolNet (Müssel et al., 2010). Simulations were performed by applying the SAT-based attractor search algorithm.

First, analyses of the general network dynamics were performed by exhaustive attractor-search in the entire state space. Resulting attractors were compared to described HSC phenotypes. Here, one attractor was excluded from further analyses due to a complete lack of external stimulation. This does not correspond to a biologically plausible condition. In a further step, progressions from one attractor to the next were studied. We used the attractor pattern of each HSC phenotype as a starting state and altered the external niche stimuli to trigger the cascade. Finally, paths from one attractor to the next were examined. Altogether, we analyzed trajectories from:

- LT-HSC to ST-HSC by activating external cycling stimuli
- ST-HSC to cycling HSC by inactivating external quiescence stimuli
- LT-HSC to cycling HSC by combining activation of external cycling stimuli and inactivation of quiescence stimuli.

We simulated all possible single-compound perturbations (knockout and overexpression, see Supplementary Section 5). To validate the model, *in silico* intervention studies were performed and compared to known mouse phenotypes. Knockouts were obtained by fixing the state of desired node(s) constantly to 0. Dynamic analyses of the considered interventions were simulated exhaustively and attractors were analyzed.

All scripts to run the simulations are provided on github (https://github.com/sysbio-bioinf/HSC-boolean-network-model) and reported in the Supplementary Section 6.

### 2.4. Robustness Analysis

We applied stochastic noise to our Boolean network model to evaluate the robustness—and thus the significance—of our model and the applied simulations. First, the ability of the network to compensate noise has been computed based on its attractors and their robustness against noise in the form of random bit flips in their basin of attraction. In our setup, we created a set of 1 million random initial states and computed the attractors for all of these states individually. Next, we created a perturbed copy of each drawn initial state and applied attractor search on all these perturbed states. For perturbation, we used random bit flips (assignments of selected nodes were toggled for 1 to 0 and vice versa). Flipping nodes in the networks' states is a common approach to simulate noise in Boolean networks (see, Qu et al., 2002; Aldana and Cluzel, 2003; Kauffman et al., 2004). To evaluate the robustness of the network, we compared the resulting attractor of each random state and its perturbed copy. Consequently, we could assess if this perturbation causes a switch of the attractors toward different basins. This procedure was exerted with toggling a different amount of random nodes to simulate an increasing amount of noise. We ran the simulation repeatedly, flipping 1, 2, and 3 nodes of each random state. For each number of bit flips, we repeated the simulation three times.

As a second measure for robustness, we analyzed the impact of noise in the model and compared it with randomly generated Boolean networks. Again, random bit flips are applied by adding noise to the network. Then, the corresponding successor state of the original one as well as of the perturbed state is computed. Then, the distance between the two successor states is measured using the normalized Hamming distance. The Hamming distance measures the number of differing bits across two state vectors. It can be formally described as $H\left(x,y\right)=\overset{n}{\underset{i}{\sum }}x_{i}⊕y_{i},\forall x,y\in {𝔹}^{n}$. The normalization is done by dividing the measured Hamming distance by the number of bits in the vectors, $HD\left(x,y\right)=\frac{H\left(x,y\right)}{n},\forall x,y\in {𝔹}^{n}$. The distance is an indicator of the ability of the Boolean network to maintain its functionality under noisy conditions. A normalized Hamming distance of zero indicates that the mutation has no effect on evaluated network behavior. The procedure was repeatedly done for 1,000 randomly drawn states. The number of bit flips for the perturbation was set to one. Finally, results were compared to the ones obtained by 1,000 randomly generated Boolean networks. For this computerintensive test, we computed a p-value to evaluate if our null hypothesis “the hamming distance for the constructed network is larger or equal than the distance for the random networks” can be rejected, as follows : $\frac{\overset{r}{\underset{i}{\sum }}\left(HD_{rnd_{i}}>HD_{c}\right)}{r}$. Here *HD*_*rnd*_*i*__ represents the mean Hamming distance over all states of one random network *rnd*_*i*_ and *r* is the total number of random networks tested. *HD*_*c*_ represents the mean normalized Hamming distance over all measured states of the constructed network. We considered *p* < 0.001 to be significant. This test procedure is included in the BoolNet package (Müssel et al., 2010). The final aim of this analysis is to test the robustness of the constructed Boolean network model compared to a number of randomly generated ones.

## 3. Results

### 3.1. Modeling the HSC Phenotypes

The HSC regulation in the niche is of crucial relevance for the maintenance of the hematopoietic system. To unravel HSC maintenance mechanisms in homeostatic conditions, we constructed a Boolean network model based on extensive literature research (Table 1). A version of Table 1 endowed with references is available in our Supplementary Section 1.1.

**Figure 1:**
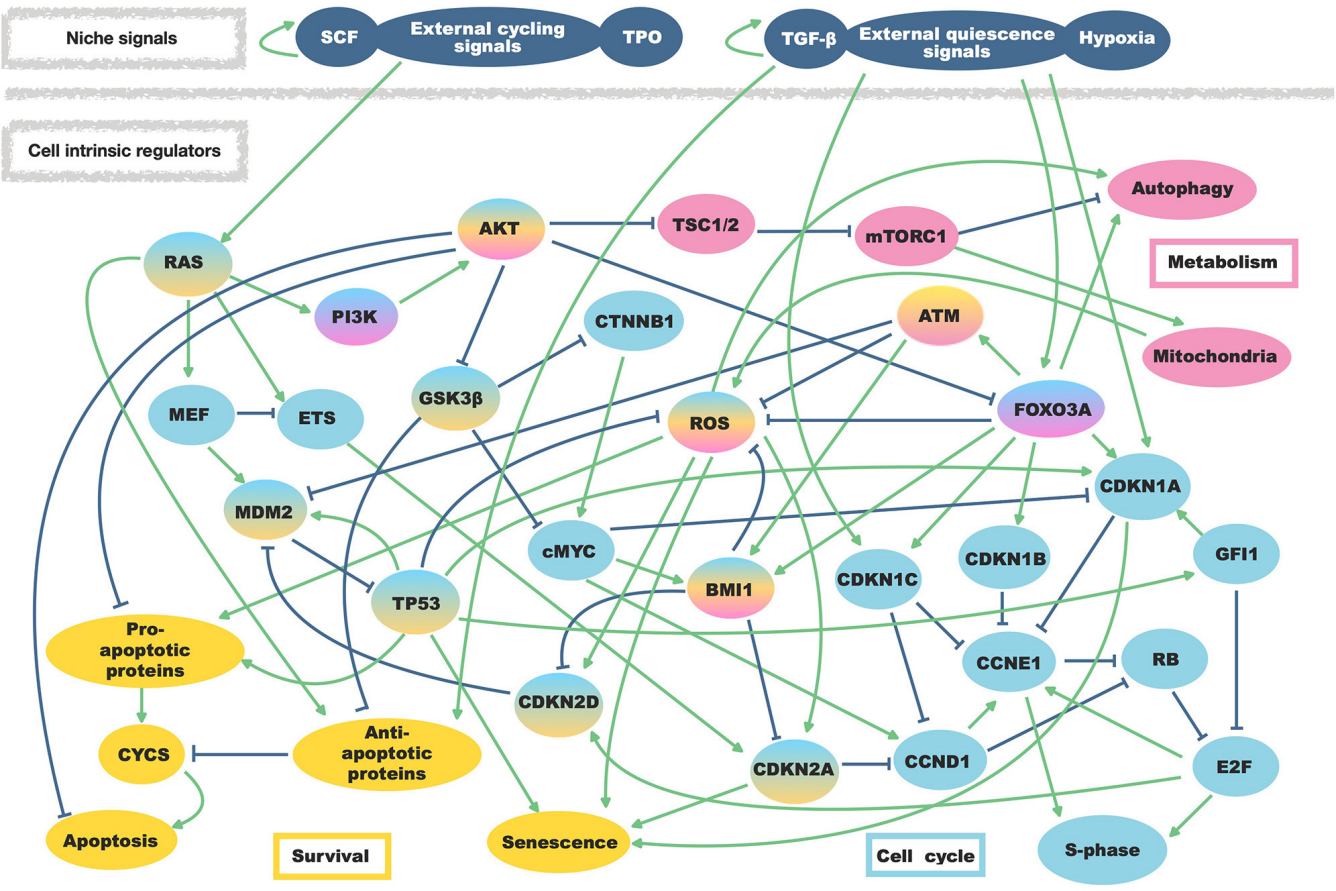
Regulatory interactions of the HSC model. External niche factors as well as regulations within the hematopoietic stem cells are summarized in one model. Regulatory activating interactions are depicted by arrows while bar-headed lines represent inhibiting regulations. Affected mechanisms and involved proteins are separated by colors.

Here we considered primary intrinsic regulators of HSC quiescence and niche external stimuli involved in their regulation. As intrinsic regulatory factors, we included cell cycle regulators (such as BMI1, MYC protoncogene (cMyc), TP53 together with cyclin-dependent kinases inhibitors (CDKN) and cyclins), proliferative pathways involving phosphoinositide 3-kinase (PI3K) and rat sarcoma (RAS), regulators of ROS, regulators of apoptosis and all potential cross-regulation among them. Furthermore, we also included a node describing autophagy in light of possible enlargement of our work toward aging studies (Ho et al., 2017). Extrinsic stimuli from the niche have been summarized as quiescence signals able to induce cell cycle inhibitors, or as cycling signals activating the RAS/PI3K axis. In total, the network consists of 37 nodes and 78 regulatory interactions (Figure 1).

**Figure 2:**
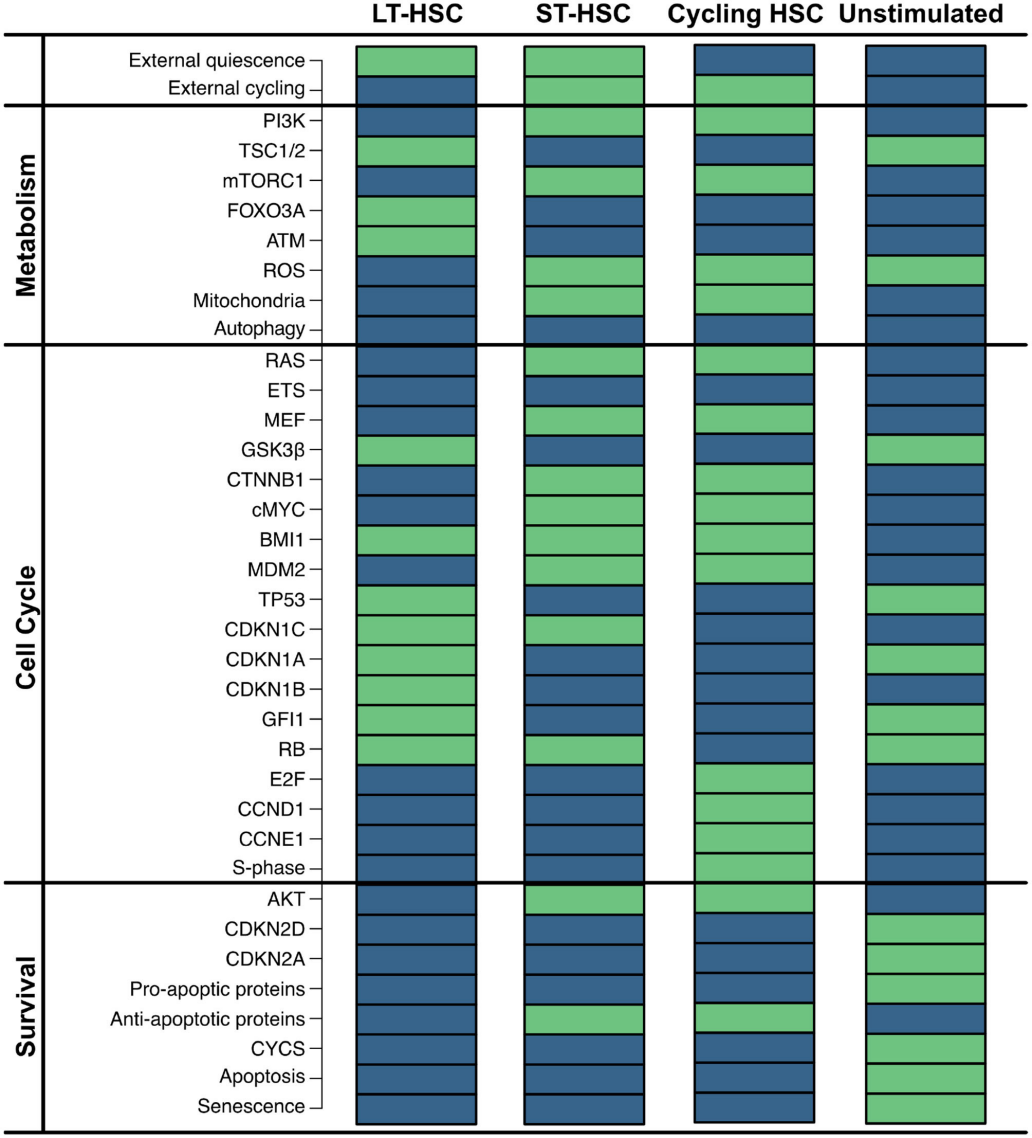
Impact of external stimulation. Simulation of the HSC model revealed four attractors that are dependent on the combinations of the two external inputs (external quiescence an external cycling). Components of the model are listed on the left separated into assigned pathways. The state of each component is depicted by colored rectangles. Green indicates active nodes, blue indicates inactive ones.

First, we exhaustively simulated our model, obtaining four single state attractors. The network contains two external inputs (external quiescence and external cycling), resulting in four possible combinations. In accordance, each reached attractor shows a different combination of these nodes (Figure 2). Given that these input nodes represent niche stimuli, a condition with any stimulation from the environment is considered not realistic. Therefore, we do not consider this condition for further analyses.

Attractor 1 is affected by quiescent external stimulation, while external cycling factors are absent. This attractor shows neither activation of the cell cycle [synthesis phase (S-phase) and cyclins] nor active metabolism or cell growth [mammalian target of rapamycin complex 1 (mTORC1) and ROS]. Instead, many inhibitors of the cell cycle [cyclin-dependent kinase inhibitor 1C (CDKN1C), cyclin-dependent kinase inhibitor 1A (CDKN1A), cyclin dependent kinase inhibitor 1B (CDKN1B), GFI1], as well as ROS and mTORC1 signaling [Forkhead box O3 (FOXO3A), ATM, Tuberous sclerosis protein 1/2 (TSC1/2)] are active. Furthermore, no apoptosis is observable, although TP53 is active. However, pro-apoptotic proteins and ROS that are required for apoptosis induction are inactive. This attractor pattern matches the behavior of a dormant LT-HSC which is considered as a reservoir of the HSCs and typically found in the endosteal region (Kopp et al., 2005; Sugiyama et al., 2006; Geiger et al., 2007; Jones and Wagers, 2008; Wagner et al., 2008; Guerrouahen et al., 2011). The attractor pattern was compared to the expression levels of quiescent HSCs. Here, low ROS (Chen et al., 2008, 2009; Ludin et al., 2014), AKT/mTORC1 (Jang and Sharkis, 2007; Chen et al., 2008, 2009; Ludin et al., 2014; Rodgers et al., 2014; Cabezas-Wallscheid et al., 2017; Baumgartner et al., 2018), RAS (Geest and Coffer, 2009), and cMYC (Wilson et al., 2004; Forsberg et al., 2005; Cabezas-Wallscheid et al., 2017) have also been observed. Additionally, quiescence is described to be maintained by expression of FOXO3A and ATM (Suda et al., 2011; Bakker and Passegué, 2013; Ludin et al., 2014) as well as TP53 activity (Forsberg et al., 2005; Liu Y. et al., 2009; Pant et al., 2012).

Attractor 2 is affected by the activity of both external stimuli. Similarly to attractor 1, the cell cycle (S-phase) is not active based on these external conditions. Hence, this attractor also shows quiescent behavior. However, this time RAS/PI3K signaling as well as mTORC1, ROS, and cMYC are active. Moreover, in attractor 2, many cell cycle inhibitors (CDKN1A, CDKN1B, GFI1) and ROS regulators (FOXO3A and ATM) are inactive. In comparison to attractor 1, apoptosis is still inactive. This might be due to inactive TP53 and the activation of the anti-apoptotic protein. Attractor 2 resembles the phenotype of an ST-HSC. The observed attractor pattern is comparable with the expression levels of ST-HSC (Wilson et al., 2004; Forsberg et al., 2005; Jang and Sharkis, 2007; Chen et al., 2008, 2009; Geest and Coffer, 2009; Liu Y. et al., 2009; Ludin et al., 2014; Rodgers et al., 2014; Cabezas-Wallscheid et al., 2017; Baumgartner et al., 2018). Moreover, in our attractor, the main cell cycle inhibitor CDKN1C is still active. It is also consistent with experimental observations indicating CDKN1C as the crucial regulator of quiescence in HSC (Umemoto et al., 2005; Matsumoto et al., 2011; Tesio and Trumpp, 2011; Zou et al., 2011) and is maintained by several quiescence-inducing external stimuli (Scandura et al., 2004; Qian et al., 2007; Blank and Karlsson, 2015).

The presence of cycling stimulation regulates attractor 3. Here, proliferative components such as cyclins are active, while cell cycle inhibitors are inactive. Thus, a proliferative phenotype can be assumed (Orford and Scadden, 2008; Pietras et al., 2011).

To sum up, our model is able to recapitulate the three main phenotypes observed for HSC, which we summarized again in Table 2. A version of Table 2 with references for each phenotype describes is provided in the Supplementary Section 1.2. Moreover, we are able to show that intrinsic stem cell behavior is affected by external stimulation. This indicates that complex niche-stem cell interactions determine the overall long-term behavior of HSCs.

**Table 2:** Summary of analyzed attractors.

**Attractor**	**Process**	**Phenotypical description**	**Associated HSC phenotype**
LT-HCS	Metabolism	Inactive ROS	Quiescent
	Metabolism	Inactive mTORC1	
	Metabolism	Active FOXO3A	
	Cell cycle	Inactive MYC	
	Cell cycle	Active TP53	
	Cell cycle	Active CDKN1C	
	Cell cycle	Active CDKN1A	
	Cell cycle	Active CDKN1B	
	Cell cycle	Active GFI1	
	Cell cycle	Inactive S-phase
ST-HSC	Metabolism	Active ROS	Activated HSC
	Metabolism	Active mTORC1	
	Metabolism	Inactive FOXO3A	
	Cell cycle	Active MYC	
	Cell cycle	Inactive TP53	
	Cell cycle	Active CDKN1C	
	Cell cycle	Inactive S-phase	
Cycling HSC	Cell cycle	Active CCND1	Proliferating HSC
	Cell cycle	Active CCNE1	
	Cell cycle	Active S-phase	
Unstimulated	External Stimuli	–	–

*Main nodes discussed in the text, final biological interpretation and references are reported for each obtained attractor. A version with references is provided in the Supplementary Section 1.2*.

### 3.2. Awakening of the HSC: Progression From Dormancy to Cycling

**Figure 3:**
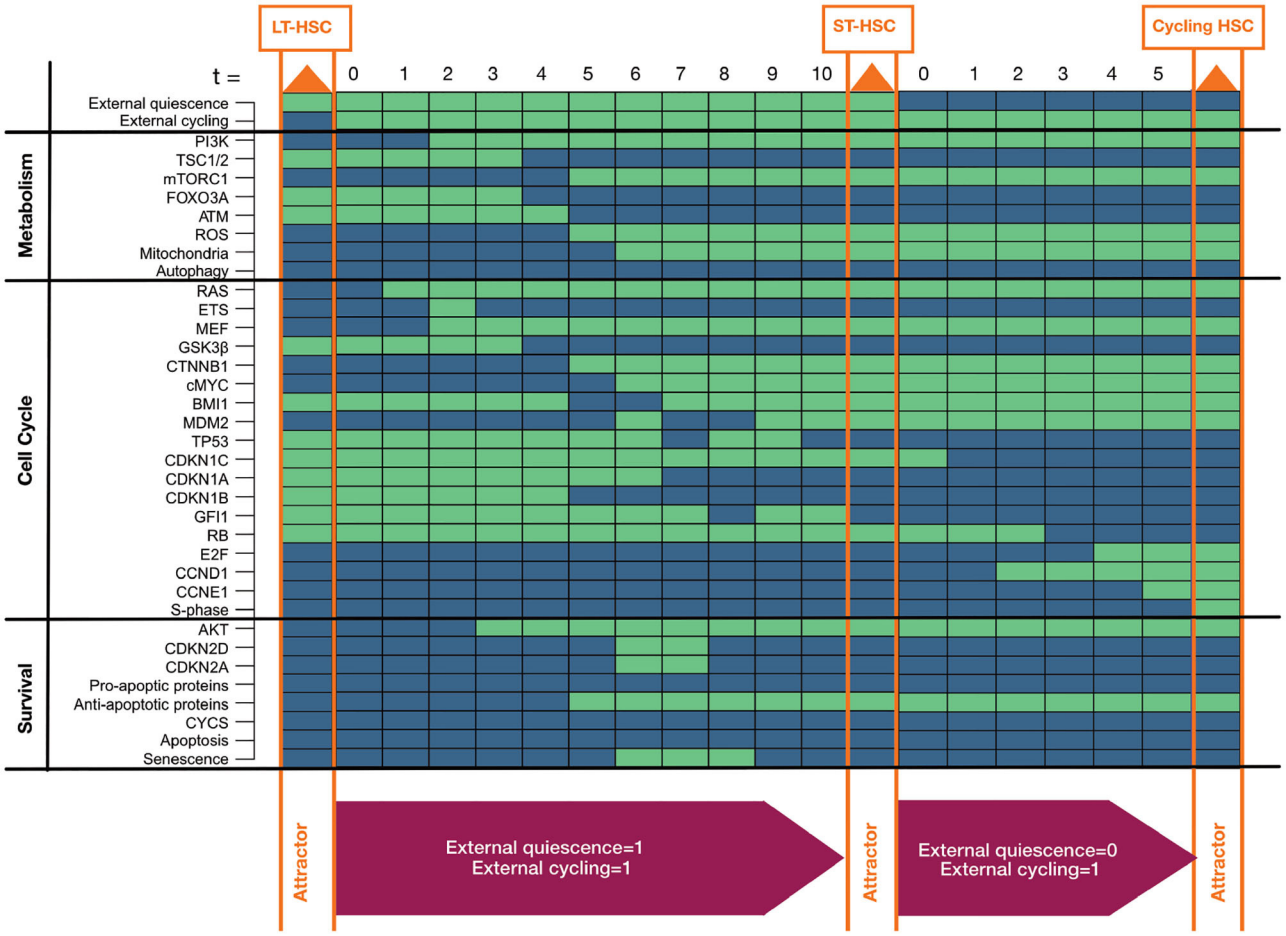
Progression toward awakening. The figure shows progression from LT to ST to cycling HSC depending on activation of external stimuli. Attractors are highlighted in orange in the progression. Activated external stimuli are depicted in magenta boxes below. Components of the model are listed on the left separated into assigned pathways. The state of each component is depicted by colored rectangles. Green indicates active components, blue indicates inactive ones.

Above, we showed the presence of different individual phenotypes in the HSC model. Furthermore, we believe that distinct phenotypes depend on each other. To support this assumption, we investigated whether the single HSC entities transit into each other by alterations of external niche factors (Figure 3). This hypothesis is in line with previous findings indicating that LT-HSCs are activated and transit to ST-HSCs which further activated proliferation (Wilson and Trumpp, 2006; Wilson et al., 2007, 2009; Foudi et al., 2009; Pietras et al., 2011). Since we already excluded the possibility of no external stimulation, there are three remaining influences from the niche: only quiescent stimuli, only proliferative (cycling) stimuli, or both stimuli are present.

Hence, we started our simulation by the LT-HSC attractor. We used this attractor state and further activated the external cycling input. This progression leads to a single state attractor describing the ST-HSC phenotype (Figure 3). Finally, form the obtained attractor pattern, we further inactivated the external quiescence node and simulated the progression toward the attractor again.

Our simulation of the awakening process started from the LT-HSC matched attractor. This attractor is characterized by active cell cycle inhibitors (CDKN1A, CDKN1B, CDKN1C, GFI1) and inactive RAS/PI3K pathways. The impact of both external stimuli was investigated. The progression toward the attractor is takes ten-time steps (Figure 3). First, the presence of external quiescent stimuli leads to up-regulation of cell cycle inhibitors (CDKN1C, CDKN1A, CDKN1B). However, the additional presence of the external cycling stimuli leads to a stabilization of RAS and PI3K signaling (time step zero to four). Furthermore, the stabilization of RAS and AKT induces the activation of cMYC by downregulation of GSK3β. In turn, cMYC leads to loss of cell cycle inhibitor CDKN1A (time step three to seven).

On the other hand, AKT activity induces mTORC1 signaling and represses FOXO3A (time steps three to seven). Thus, activation of mTORC1 directly activates mitochondria and the production of ROS. ROS activation is further sustained by the inhibition of FOXO3A and, consequently, ATM (time steps three to seven). Loss of ATM in the absence of cyclin-dependent kinase inhibitor 2D (CDKN2D) stabilizes MDM2 that, in turn, downregulates TP53 and its downstream target GFI1 (time steps six to ten). Stabilization of active MDM2 is further maintained by activation of myeloid elf-1-like factor (MEF) in response to RAS activation (from time step two). The finally reached attractor is the one that was previously connected to an ST-HSC. This cascade, including both external niche factors, indicates a destabilization of TP53 based on the activity of RAS/AKT and their downstream targets. Finally, starting from the ST-HSC attractor, we simulated our system by removing external quiescence stimuli. This time the system reaches a cycling HSC phenotype in five-time steps. Due to lack of external quiescence input, the phenotype switches toward further evolution of states leading to entry in the cell cycle. In fact, the lack of active cell cycle inhibitors leads to regulation of Retinoblastoma protein (RB), Cyclin D (CCND1) and Cyclin E (CCNE1), and finally to activation of the S-phase entry (time steps one to four) and thus proliferation.

Further, we considered the possibility that a highly quiescent HSC can be directly driven to enter the cell cycle. Hence we considered activation of external cycling stimulation starting from the LT-HSC attractor. The progression toward the cycling attractor presents a similar cascade, as shown in Figure 3, and is reported and briefly discussed in the Supplementary Section 2.

Summarizing these simulations, our model is not only able to recapitulate the different phenotypes of an HSC (Figure 2) but also shows that they depend on each other (Figure 3 and Supplementary Section 2). This indicates a dependency of HSC behavior from niche factors. However, we do not want to underestimate the impact of intrinsic rewiring regulations. The switching cascade between HSC entities that mTORC1 signaling and ROS regulation play a significant role in reasserting the final phenotypes. Besides, we observed that different mechanisms could govern the quiescence status of HSC, making them more or less reactive to external stimuli (Figure 3). Starting from an ST-HSC phenotype, the progression toward a cycling HSC happens in fewer time steps than considering an LT-HSC (Figure 3 and Supplementary Section 2). The results of our progressions are in accordance with the fact that ST-HSCs are thought to be more active HSCs: they preserve a quiescent state but can enter the cell cycle (Wilson et al., 2004, 2007, 2009; Foudi et al., 2009).

### 3.3. Balancing Quiescence: A Novel Mechanism for TP53 Regulation in HSC

TP53 activity is known to be downregulated by MDM2. Furthermore, MDM2 expression is triggered by TP53 itself, setting a negative feedback loop in its own regulation (Jones et al., 1995; Montes de Oca Luna et al., 1995; Ringshausen et al., 2006). However, we assume that this general mechanism is somehow uneven in HSCs to allow TP53 activity and maintenance of quiescence.

In the previous section, we showed progression toward different HSC phenotypes (Figure 3 and Supplementary Section 2). In our simulation we observed alterations in the TP53 regulation during the cascades leading to the different attractors. In fact, in LT-HSC the activity of TP53 is maintained through a FOXO3A-ATM axis that destabilizes MDM2 activity. In ST-HSC, instead, external cycling signals cause an up regulation of the RAS/PI3K axis. This activation has two major effects that down regulate TP53. First, AKT activation causes a down regulation of FOXO3A and ATM (Figure 3, time steps three to seven). Second, RAS activation upregulates MEF, a transcriptional activator of MDM2. As a result, MDM2 and TP53 as well as its downstream target the cell cycle inhibitor GFI1 are downregulated. Hence, we believe that TP53 activity in steady state conditions is balanced by a crosstalk of intrinsic and extrinsic factors, finely tuning its activity in regulating HSC quiescence. This general regulatory hypothesis will be further discussed in the following section.

### 3.4. *In silico* Interventions on the Model

The hypothesized mechanism of TP53 regulation was further investigated by performing *in silico* knockouts. In the following, we compare our interventions to phenotypes of mouse models. To verify our model's prediction power, we assessed general long-term behavior and its impact on TP53.

#### 3.4.1. FOXO3A and ATM Knockouts: Guardians of ROS Regulation

**Figure 4:**
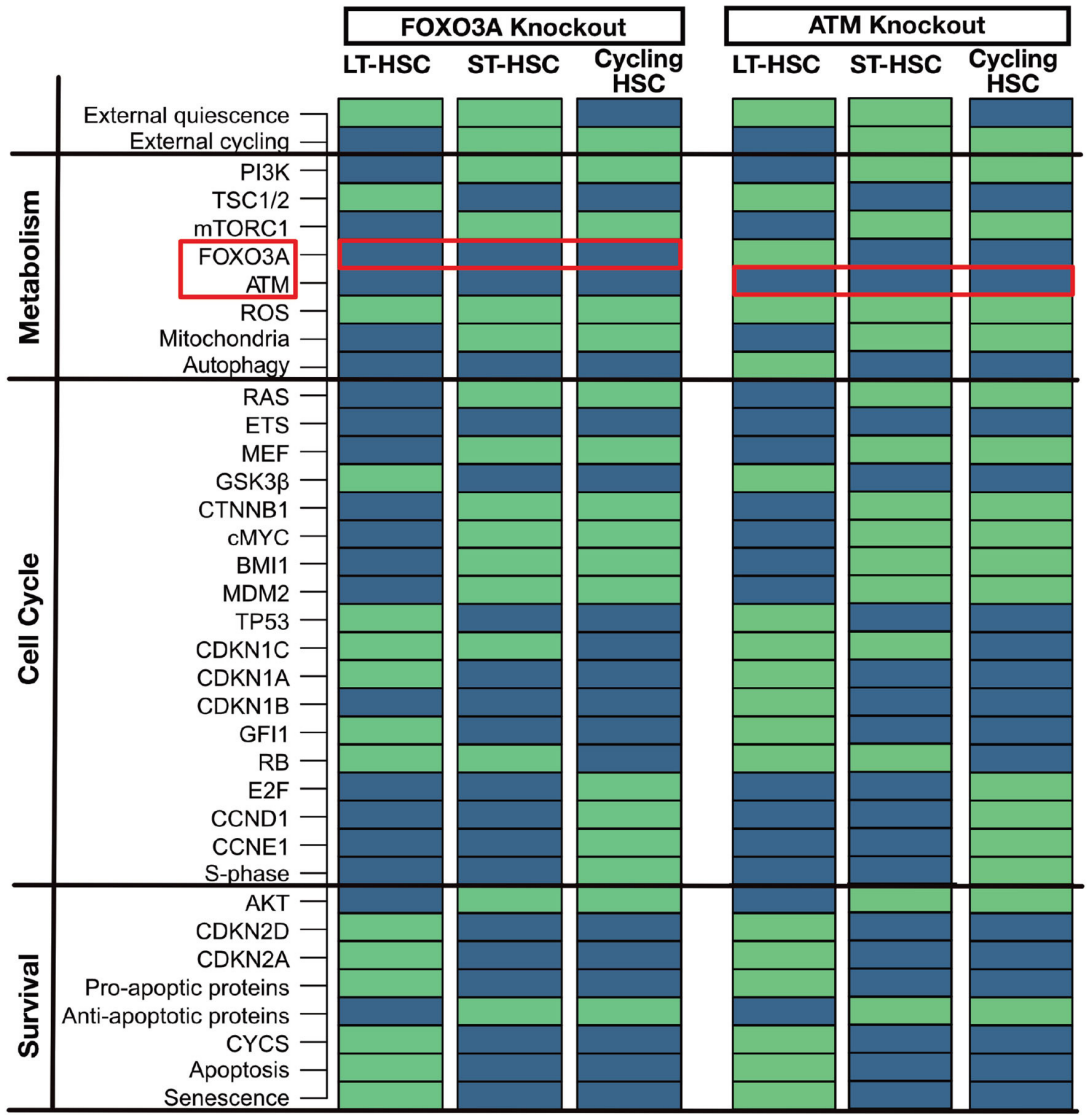
Similar phenotypes of ATM and FOXO3A knockout. An *in silico* knockout of FOXO3A or ATM increases ROS levels but only induces apoptosis and senescence in the LT-HSC population. Proteins are listed on the left with their pathway affiliations. The state of each protein is represented by colored rectangles. Activity is represented in light green and inhibition in dark blue. For simplicity due to the highly similar phenotype of the two *in silico* knockouts only FOXO3A is reported.

FOXO3A and ATM are two significant regulators of ROS in quiescent HSCs (Bakker and Passegué, 2013; Ludin et al., 2014). Mouse models for FOXO3A knockout (Tothova and Gilliland, 2007; Tothova et al., 2007; Yalcin et al., 2008; Maryanovich et al., 2012) and ATM knockout (Ito et al., 2004, 2006; Maryanovich et al., 2012) show increased ROS, CDKN2D, and CDKN2A levels in the HSC compartment leading to senescence and impaired survival. This leads impaired self-renewal and repopulation ability of the LT-HSCs. However, it is still possible to observe a normal differentiation in hematopoiesis (Ito et al., 2004, 2006; Tothova and Gilliland, 2007; Tothova et al., 2007; Yalcin et al., 2008; Maryanovich et al., 2012). The observed phenotypes within the mouse models could be matched to our attractors (Figure 4). LT-HSC shows senescence and apoptosis activation and increased ROS levels due to the activation of CDKN2A and CDKN2D. In particular, activation of CDKN2D sustains TP53 activation. The latter, in the presence of ROS, can trigger apoptosis and senescence. ST-HSCs are less affected and do not undergo apoptosis due to the activation of anti-apoptotic proteins and already inactive FOXO3A and ATM. Also, the cycling HSC attractor is not altered. This result is also in line with our cascades showed in Figure 3. There it is shown that FOXO3A and ATM are already inactivated in ST-HSC after activation of PI3K/AKT signaling. To summarize, in the FOXO3A and ATM knockouts, our attractors show significant perturbations in the LT-HSC population. Here, activation of ROS, CDKN2A, CDKN2D leads depletion of the LT-HSC population. However, the other two attractors, namely the one representing ST-HSC and cycling HSC are not affected by the perturbation. This might explain the normal hematopoiesis seen in the FOXO3A and ATM mouse models (Ito et al., 2004; Yalcin et al., 2008). Moreover, in the FOXO3A depleted mice, it was shown that the depletion of the HSCs comes from the LT-HSC population (Yalcin et al., 2008), supporting our population-based results.

#### 3.4.2. BMI1 Knockout: Effect and Rescue of the Gatekeeper of Self-Renewal

**Figure 5:**
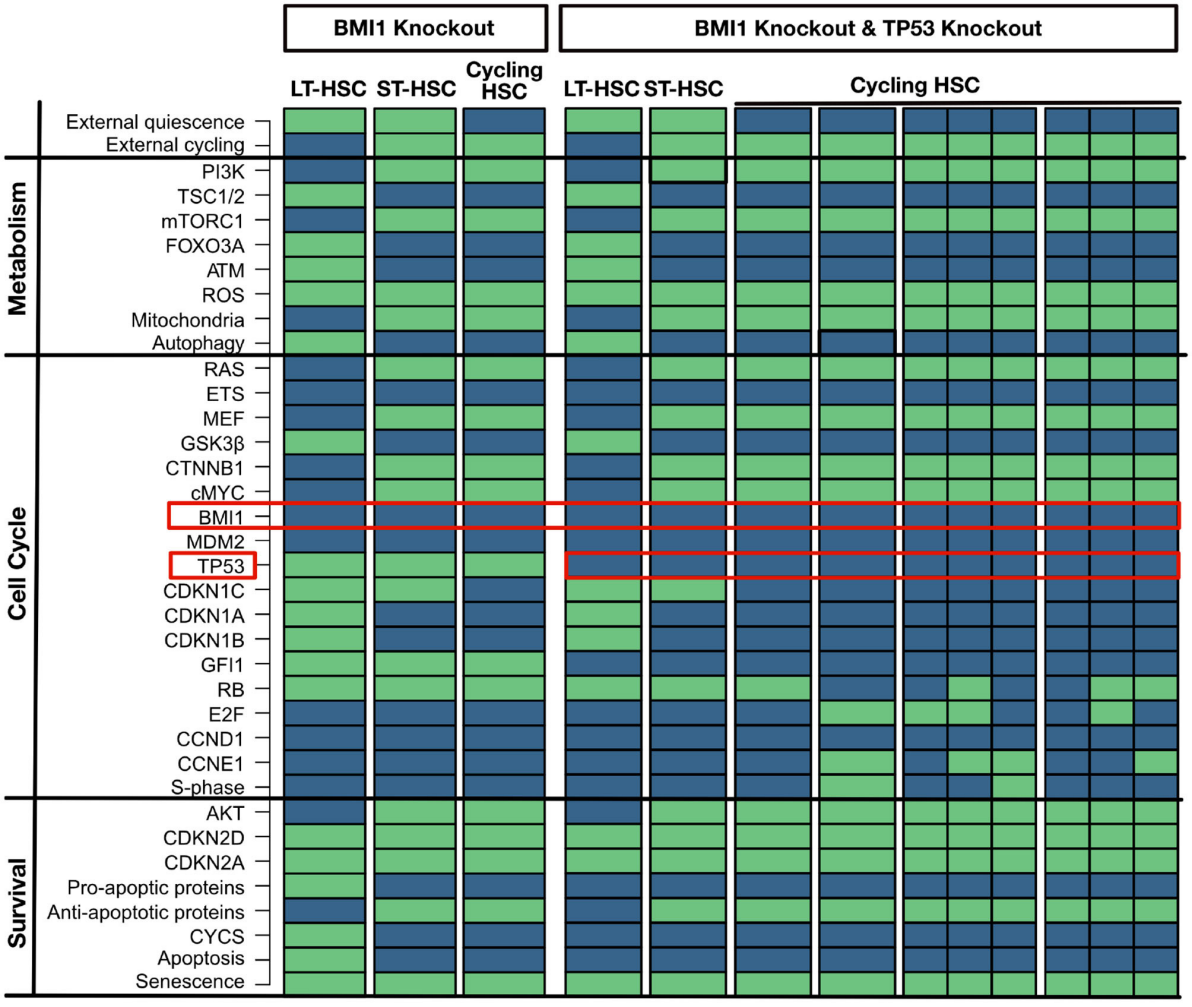
BMI1 loss impairs self-renewal (left part). An *in silico* knockout of BMI1 impairs cell cycle entry for all HSC populations. Proteins are listed on the left with their pathway affiliations. TP53 partially rescue a BMI1 knockout (right part). An *in silico* knockout of BMI1 and TP53 rescues a the impact of a loss of BMI1 by impairing apoptosis. Proteins are listed on the left with their pathway affiliations. The state of each protein is represented by colored rectangles. Activity is represented in light green and inhibition in dark blue.

BMI1 is a crucial regulator of self-renewal and is widely expressed in the HSC compartment. Therefore, the BMI1 knockout simulation shows a huge effect on the phenotype in mice leading to defect in self-renewal, depletion of the HSC compartment, and shortened life-span (Park et al., 2003; Liu J. et al., 2009; Rizo et al., 2009). This effect is accompanied by increased ROS levels and increased CDKN2A and CDKN2D expression, which finally induces apoptosis and senescence (Park et al., 2003; Liu J. et al., 2009; Rizo et al., 2009). These huge implications are also observable in the attractor pattern. Here, BMI1 loss induces a substantial impairment of cell cycle entry for the HSC in all obtained attractors (Figure 5). All attractors show senescence, and the LT-HSC attractor shows apoptosis additionally. The attractor for the cycling HSC does not show activation of S-phase. Based on our cascades shown in Figure 3, loss of BMI1 upregulates CDKN2A and CDKN2D, causing impaired quiescence and activation maintenance among the three phenotypes.

Various possibilities to partially rescue the phenotype of BMI1 loss have been suggested. One of these suggestions is a loss of TP53 which impairs apoptosis (Akala et al., 2008; Warr et al., 2011). This behavior can also be seen in the attractor when TP53 is perturbed together with BMI1 (Figure 5). Again, these simulations show that an increase of ROS and CDKN2D activates TP53 and guide its activity toward pro-apoptotic behavior.

#### 3.4.3. TP53 and MEF Knockouts: Perturbation of Quiescence

**Figure 6:**
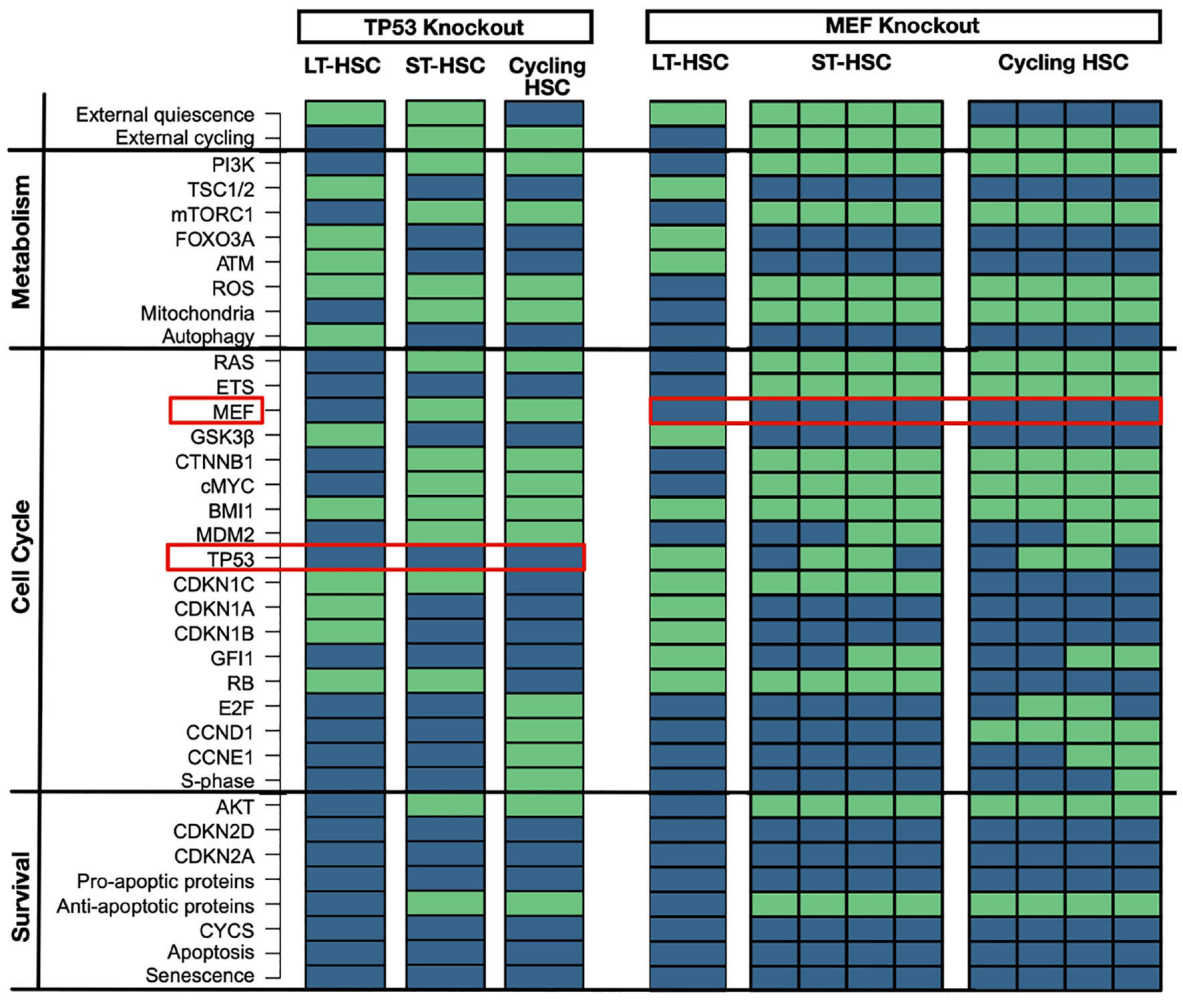
TP53 and MEF knockouts. TP53 knockout has minor impact on HSC populations. An *in silico* knockout of TP53 causes loss of GFI1 in LT-HSCs and increased ROS levels. ST-HSC and cycling ones are not impacted. Proteins are listed on the left with their pathway affiliations. The state of each protein is represented by colored rectangles. Activity is represented in light green and inhibition in dark blue. MEF knockout stabilizes TP53. An *in silico* knockout of MEF stabilized TP53 and enhanced quiescence. Here, ST-HSC and cycling HSCs show activity of GFI1 and cycling HSCs are impaired in entering S-phase. Proteins are listed on the left with their pathway affiliations. The state of each protein is represented by colored rectangles. Activity is represented in light green and inhibition in dark blue.

TP53 knockout has no major implications on hematopoiesis in mice (TeKippe et al., 2003; Liu Y. et al., 2009; Abbas et al., 2011; Pant et al., 2012). However, loss of GFI1 gives an advantage in competitive assay due to less regulated quiescent state (TeKippe et al., 2003; Liu Y. et al., 2009; Abbas et al., 2011; Pant et al., 2012). A matching attractor pattern is observable in the *in-silico* simulation with no significant induction of apoptosis (Figure 6). Moreover, even in our LT-HSC attractor GFI1 gets inactivated. The inactivation of GFI after TP53 was observed in our previous results in the transition between LT and ST-HSC. Hence, our LT-HSC TP53 knockout attractor matches now partially with the ST-HSC (Figure 2). Our attractors show also activation of ROS in line with literature results (Liu et al., 2008). Activation of ROS in HSCs has also been connected to a more active state, prone to enter the cell cycle (Bakker and Passegué, 2013; Ludin et al., 2014). Altogether, our results are following experimental observations on TP53 knockout models. After accessing the loss of TP53, we further analyzed the effects of its over stabilization in HSCs. For this reason, we selected another mouse model causing MDM2 downregulation via MEF deletion. MEF knockout mice show a stabilization of TP53 connected to an enhancement of quiescence (Lacorazza et al., 2006; Liu Y. et al., 2009). Likewise, attractor for cycling HSC showed enhanced quiescence due to loss of S-phase. In accordance also the ST-HSC attractor shows now TP53 and GFI1. The stabilization of TP53 affects the transition from one HSC phenotype to another, mainly by the maintenance of GFI1 activity. The unperturbed cascade shows that loss of GFI1 activity happens during the transition between LT- and ST-HSC (Figure 3). Hence, in our *in silico* simulations, stabilization of TP53 causes an enhanced quiescent phenotype (Figure 6).

**Figure 7:**
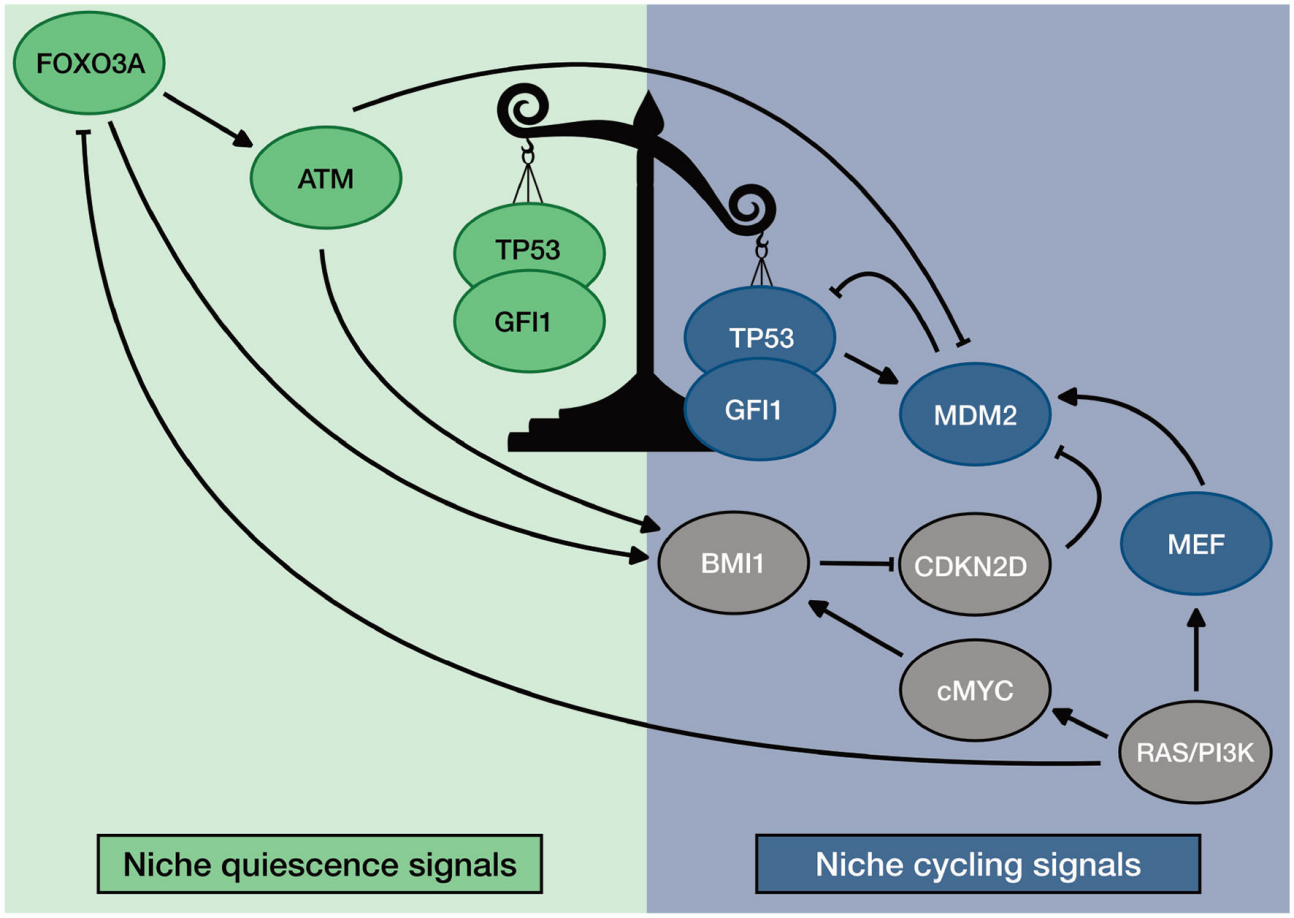
General description of TP53 regulation in HSC. Homeostatic activity of TP53 is promoted by ROS regulators ATM and FOXO3A. Unbalance of TP53 activity is promoted by MEF that stabilizes the transcription of MDM2. Furthermore, the unbalanced activity of TP53 is maintained by niche regulation: quiescence promoting environment favors ROS regulators; Cycling promoting environment promotes MEF expression.

Finally, we showed that our model could recapitulate the phenotype of published mouse models. Moreover, perturbation results sustain our hypothesis concerning the general mechanism of regulation of TP53 in HSCs. When ATM, FOXO3A, or BMI1 are depleted, increased ROS and CDKN2D lead to activation of TP53. This effect switches its activity toward the induction of apoptosis and senescence. In homeostatic conditions, instead, they guarantee TP53 activity toward quiescence by downregulating ROS and CDKN2D. Our simulations also showed the relevance of the RAS/PI3K axis in down regulating TP53 when HSCs are activated. The loss of MEF down regulates MDM2 in ST- and cycling HSCs. This loss causes an impaired entry in the S-phase despite the presence of external cycling stimuli. Altogether, our results suggest a general mechanism of regulation of TP53 activity in HSCs. The described mechanism is summarized in Figure 7.

### 3.5. Robustness Analysis

We applied stochastic noise to our Boolean network model to evaluate the robustness—and thus the significance—of our model and the applied simulation. First, the robustness of the network has been computed based on its attractors. Their robustness is measured by the addition of noise by random bit flips in their basin of attraction. We ran the simulation repeatedly, flipping 1, 2, and 3 nodes of each random state. For each number of bit flips, we repeated the simulation three times. Results showed for equal attractors for perturbed and unperturbed states with a mean and standard deviation of 95% (±0.0003)/89% (±0.0001)/85% (±0.0005) for the simulation with 1/2/3 random bit flips per initial node respectively.

**Figure 8:**
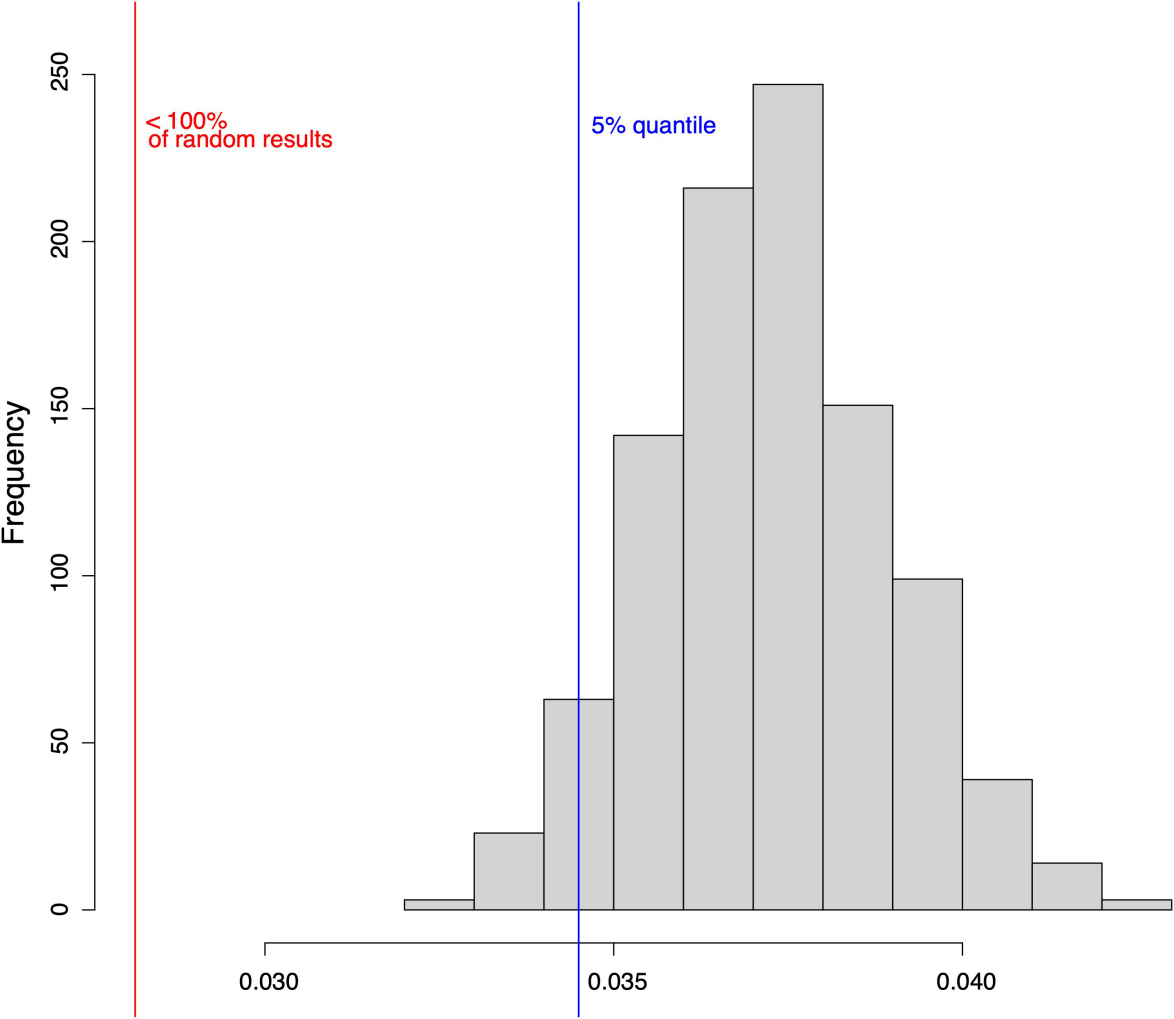
One thousand randomly drawn states of the HSC model were mutated by bit flip and their successor states were computed. The successor states of the mutated and the original states were then compared using the normalized Hamming distance (red line). The same analysis was performed for 1,000 randomly generated networks of the same size (histogram). The blue line shows the 5% quantile.

As a second measure for robustness, we analyzed the impact of noise on the state transitions in the model and compared it to randomly generated Boolean networks. As before, noise is simulated using random bit flips. We calculate the mean normalized Hamming distance between the successor states of the original state and perturbed copy. Smaller Hamming distance can be interpreted as a better ability to compensate noise. The aim of this analysis is to evaluate the transition robustness of the constructed Boolean network model compared to random networks. Results show a mean Hamming distance of 0.028 for the constructed HSC model. In contrast, the mean normalized Hamming distance over all random networks is 0.034. The calculated *p*-value is below 0.0001. Thus, we conclude that our established model is significantly more robust to noise than a set of randomly generated networks of the same size (Figure 8).

## 4. Discussion

In the present work, we proposed an *in silico* Boolean network model depicting the unique wiring that guarantees the maintenance of HSCs in their niche. Our model is able to describe the quiescence mechanism of LT-HSC and ST-HSC. Moreover, we showed the progression toward entry in the cell cycle with respect to changes in external conditions. Additionally, our model strikingly recovered several phenotypes observed in HSCs of knockout mouse models for major intrinsic regulators. This evaluation might be quite challenging under *in vitro* conditions where both isolation and maintenance of HSC require much effort. Identifying methods of isolation and maintenance of HSCs is still challenging (Passegué et al., 2005; Weissman and Shizuru, 2008; Challen et al., 2009; Rossi et al., 2011; Frisch and Calvi, 2014; Jiang et al., 2018; Kobayashi et al., 2019). Hence, the possibility of having an *in silico* mathematical model for simulating the behavior of HSC is of great interest.

External stimuli coming from the niche were summarized as either quiescent or cycling inputs. This choice might be considered an over-simplification of the complex network of signals surrounding the HSC. However, it has been shown that, due to its crucial relevance, HSC maintenance is redundantly regulated by multiple sides. Exemplarily, CDKN1C which is considered the central gatekeeper of quiescence (Passegué et al., 2005; Umemoto et al., 2005; Matsumoto et al., 2011; Tesio and Trumpp, 2011; Zou et al., 2011), is regulated by a multiplicity of endosteal stimuli including TGF-β, TPO and hypoxia (Scandura et al., 2004; Qian et al., 2007; Eliasson et al., 2010; Blank and Karlsson, 2015). Following the idea of redundancy, the loss of hypoxic conditions will not lead to a complete lack of CDKN1C activity (Eliasson et al., 2010). Also, the loss of single developmental pathways has been shown not to affect HSC maintenance, indicating again that a multiplicity of signals conquer the task (Mancini et al., 2005; Maillard et al., 2008; Gao et al., 2009; Hofmann et al., 2009; Kabiri et al., 2015; Oostendorp, 2015). Nevertheless, dissecting too much single influence of each external signal might lead to contradictory results. For example, SCF and TPO have been associated with quiescence *in vivo* (Qian et al., 2007; Yoshihara et al., 2007; Thorén et al., 2008). However, these factors are commonly used to sustain the proliferation of HSC *in vitro* (Ema et al., 2000; Gammaitoni et al., 2003). This differential behavior suggests the possibility that cross-regulation among quiescence signals exists. It has been shown that TGF-β signaling, highly present in the endosteal niche (Yamazaki et al., 2009; Blank and Karlsson, 2015), is able to repress the cytokine-induced activation of PI3K by inhibiting the formation of lipid rafts (Yamazaki et al., 2009). Besides, other mathematical modeling approaches successfully described HSC differentiation by suggesting signaling cumulative environments (Roeder and Lorenz, 2006; Roeder et al., 2008; Glauche et al., 2011). Therefore, it is reasonable to consider an environment with predominant quiescence or cycling stimuli.

Besides single predominant stimuli, we also considered the co-presence of both quiescence and cycling inducing stimuli as sort of gray zones with a balance between the two signals. This consideration is actually in line with different niche models that suggest activated HSCs to be found in regions of the niche that are boundaries between two different environments (Murphy et al., 2005; Wilson and Trumpp, 2006). In this direction Murphy et al., describe HSCs able to self-renew and proliferate to be found in the connection between endosteal and stromal area (Murphy et al., 2005). These HSCs would then sense both quiescence signals coming from the endosteal niche and proliferation stimuli coming from the stroma (Murphy et al., 2005). Also, Trumpp et al. hypothezed a similar regulation, describing the presence of a reservoir quiescent compartment with specialized cells maintaining LT-HSC (Wilson and Trumpp, 2006; Trumpp et al., 2010). Again they characterize a “self-renewing endosteal niche” as the one present at the boundaries of this region when HSCs can get activated and more responsive (Wilson and Trumpp, 2006; Trumpp et al., 2010).

Nevertheless, our results suggest a balance of external signals that sets the instructions for the intrinsic rewiring of the HSC. This means that potentially an LT-HSC could be found in any region having quiescence inducing signals. Thereby expanding the possibility of finding niches for quiescence also in different regions of the bone marrow. This consideration is in accordance to further observations that describe the presence of quiescent HSCs for example in lowly oxygenized regions of sinusoids (Itkin et al., 2016; Pinho and Frenette, 2019).

Different phenotypic characterizations of LT- and ST-HSC describing differential expression patterns have been published (Wilson et al., 2004; Forsberg et al., 2005; Passegué et al., 2005; Jang and Sharkis, 2007). Our attractors could match the primary expression and activity features that identify the two HSC phenotypes. Moreover, we also suggested two different mechanisms of maintaining quiescence in these two subpopulations. Our model indicates that LT-HSC quiescence is preserved by tight regulation of ROS levels from a combination of intrinsic factors induced by external stimuli. It has been observed that keeping oxidative stress low in quiescent HSC is fundamental for preserving their integrity and survival (Jang and Sharkis, 2007; Suda et al., 2011; Ludin et al., 2014). In fact, despite the activity of TP53 and lack of survival stimuli, we do not detect activation of apoptosis due to the highly hampering of cellular stress.

On the other hand, it has been shown that HSCs have a high expression of induced myeloid leukemia cell differentiation protein (MCL1), an anti-apoptotic protein activated in the presence of survival stimuli (Opferman et al., 2005). However, this study was performed on the entire subset of HSC and progenitor cells. Similarly, if we consider our whole set of attractors, we also observe an expression of anti-apoptotic proteins. Sustaining our hypothesis of maintenance of LT-HSC, Kobayashi et al. recently published a study describing the ideal setting for *in vitro* maintenance of quiescent HSCs (Kobayashi et al., 2019). In their work, the authors actually show that hypoxic condition in the presence of residual cytokine stimuli sets the best environment for the maintenance of quiescence (Kobayashi et al., 2019). Our dynamic simulations also lead to a single state attractor depicting a cycling HSC. Initially, this result seems in contradiction with previously published mammalian cell cycle models (Fauré et al., 2006; Tanaka et al., 2017; Diop et al., 2019). In these studies, proliferating cells are represented by cycling attractors. However, we were interested in modeling exit from Gap phase zero (G0) and entering the S-phase. Hence, we selected cell cycle regulators involved in this switch. If we consider only this regulators, also previously published cell cycle models would show a single state attractor (Fauré et al., 2006; Tanaka et al., 2017; Diop et al., 2019).

Moreover, we inserted our starting states of cell cycle regulators from our cycling HSC attractor in the Fauré et al. model and obtained a cyclic attractor (see Supplementary Section 3). Altogether, this underlines the correctness of our approach and the extendability of our network model.

Notably, we could also show that LT-HSC and ST-HSC have different responsiveness in terms of time to enter the cell cycle once stimulated. This is also in line with the observation that actually ST-HSC is more reactive and can efficiently enter the cell cycle (Forsberg et al., 2005; Murphy et al., 2005; Wilson et al., 2007, 2008). Our hypothesis or regulatory wiring is further encouraged given the predictive power that our model has toward a variety of *in silico* knockouts that can faithfully recapitulate the phenotype of analog mouse models.

Furthermore, based on our simulation of the progression from a quiescent cell toward the entry in the cycle, we can suggest a mechanism for TP53 homeostatic regulation in HSCs. Here, we propose that the awakening of the HSC follows the initial activation of cellular metabolism. This will then induce the rewiring of the intrinsic network of ROS sensors (FOXO3A and ATM) and finally lead to a loss of cell cycle regulators connected to quiescence such as TP53, CDKN1A, and CDKN1B. In the end, with loss of quiescence signals, the HSC can rapidly enter the cell cycle. Stress sensors have been shown to be fundamental for regulating HSC quiescence (Suda et al., 2011; Bakker and Passegué, 2013; Ludin et al., 2014). The expression of FOXO3A is directly connected to the hypoxic environment, and this leads in turn to the upregulation of active ATM (Yalcin et al., 2008; Suda et al., 2011). The last, besides being an independent regulator of cellular stress, is also involved in the activation of TP53 (Yalcin et al., 2008; Suda et al., 2011; Bakker and Passegué, 2013; Ludin et al., 2014). Thus, our simulations indicate that in LT-HSC ATM is responsible for the regulation of active TP53. This activation is actually new to the HSC maintenance context. This is further enforced from the fact that ATM seems to be required for TP53 immediate regulation of irradiation in quiescent HSC (Mohrin et al., 2010; Kosan and Godman, 2016). Besides its well-defined role in DNA damage response, ATM has already been shown to regulate activation of TP53 under physiological conditions in the context of homeostatic cellular metabolism outside of the HSC context (Armata et al., 2010). Here, It has been suggested that activation of TP53 by ATM is involved in the reduction of ROS (Armata et al., 2010). When instead RAS and PI3K are activated by external stimuli, FOXO3A is downregulated by AKT/mTORC1 signaling potentially also downregulating ATM. This regulation is in accordance with the fact that already in ST-HSC, FOXO3A is excluded from the nucleus (Yalcin et al., 2008). Consequently, in this condition, TP53 is mainly regulated by the MEF/MDM2 and the BMI1/CDKN2D axis (Liu Y. et al., 2009; Abbas et al., 2010; Warr et al., 2011). These regulators set the downregulation of TP53 and GFI1 in the progression toward the ST-HSC phenotype. In accordance, TP53 is downregulated in ST-HSCs (Forsberg et al., 2005; Jang and Sharkis, 2007; Pant et al., 2012). Nevertheless, our model also maintains the hypothesis that TP53 activity is context-dependent (Asai et al., 2011) as shown by our knockout simulations. To further analyze the balanced regulation of TP53 under homeostatic conditions, we performed loss of interaction mutation simulations (see Supplementary Section 4). Here, we could show the loss of regulatory axes causing disruption of the original wildtype attractors, when removing the interaction between ATM and MDM2, MEF, and MDM2 or both. In addition, constitutive FOXO3A/ATM or RAS/PI3K in absence of external cycling signals can recapitulate the TP53 activation status (see Supplementary Section 4).

Our model of progression is also in accordance with the idea that mTORC1 signaling triggers cellular metabolism and prompts the HSC to enter the cell cycle (Chen et al., 2008, 2009; Rodgers et al., 2014; Baumgartner et al., 2018). Hence, we provide an overall mechanism of HSC awakening and a new general suggestion of TP53 regulation in homeostatic conditions. To our best knowledge, no previous models could show neither HSC maintenance nor a general mechanistic explanation of TP53 regulation able to summarize observed phenotypes in different experimental conditions.

In addition to our dynamic simulations, we also investigated the robustness of our established Boolean network. Biological networks are considered to be robust toward perturbations. This means that they can adapt to environmental changes, and their functions are resistant to random noise (Kitano, 2002; Greenbury et al., 2010; Graudenzi et al., 2011; Barabási, 2016; Schwab et al., 2020). Hence, we also evaluated the robustness of our network against noise. We both investigated the shift of attractor basin after perturbation and the normalized Hamming distance after bit flip perturbation. In the latter, we compared the performance of our models to one of randomly generated networks of the same size. Equivalent approaches to the ones we presented have been already applied to investigate the robustness of Boolean networks (Joo et al., 2018; Siegle et al., 2018). Altogether, we could show that our model is robust toward random noise.

In conclusion, we established a model able to faithfully recapitulate the regulation of HSC maintenance in the presence of external niche stimuli. Also, we simulated *in silico* the process of awakening of the HSC, giving a mechanistic and overall picture of the process. Further, we suggest a new general regulatory wiring responsible for the regulation of TP53 activity in HSCs. Moreover, we further validated our model by testing its predictive power in a variety of mouse models.

## Data Availability Statement

All datasets generated for this study are included in the article/Supplementary Material. All data are deposited in github.

## Author Contributions

HK, SK, and JS devised the project. NI and SK established the model. NI, SK, and JS performed simulations and were responsible for the visualization. All authors discussed the results and contributed to the final manuscript. HK provided funding and supervised the project.

## Conflict of Interest

The authors declare that the research was conducted in the absence of any commercial or financial relationships that could be construed as a potential conflict of interest.
