# Supplementary material for: Awakening the HSC: Dynamic Modeling of HSC Maintenance Unravels Regulation of the TP53 Pathway and Quiescence
Source: Front Physiol. 2020 Jul 31;11:848. doi: 10.3389/fphys.2020.00848 (PMC7411231; doi:10.3389/fphys.2020.00848)
Supplement: Supplementary file 1 [file Data_Sheet_1.ZIP › frontiers_SupplementaryMaterialRev2.pdf]

Supplementary Material

1 SUPPLEMENTARY TABLES

1.1 Supplementary Table 1

Below, we provide the table containing our Boolean functions for the HSC model together with references for each of them.

Table S1: Boolean functions for the HSC model. Compounds are abbreviated according to accepted nomenclature. Regulatory interactions are summarized by logical connectives AND (∧), OR (∨) and NOT (¬).

Node	Boolean function	References
External quiescence	External quiescence	Blank and Karlsson (2015); Pietras et al. (2011a); Eliasson and Jönsson (2010); Piccoli et al. (2013); Scandura et al. (2004); Suda et al. (2011a)
External cycling	External cycling	Ema et al. (2000); Kaushansky (2009); Kumar and Geiger (2017); Lee et al. (2020); Martelli et al. (2010); Pietras et al. (2011a); Pinho and Frenette (2019); Varghese et al. (2017)
PI3K	RAS	Chung et al. (2011); Hemmati et al. (2019); Martelli et al. (2010)
TSC1/2	¬AKT	Martelli et al. (2010); Pietras et al. (2011a); Warr et al. (2011)
mTORC1	¬TSC1/2	Ludin et al. (2014a); Martelli et al. (2010); Pietras et al. (2011a); Warr et al. (2011)
FOXO3A	External quiescence ∧ ¬AKT	Bakker et al. (2007); Brunet et al. (1999, 2002); Martelli et al. (2010); Storz (2011); Suda et al. (2011a)
ATM	FOXO3A	Yalcin et al. (2008)
Mitochondria	mTORC1	Chen et al. (2008); Gan et al. (2008); Juntilla et al. (2010); Ludin et al. (2014a); Yilmaz et al. (2006)

Table S1: Boolean functions for the HSC model. Compounds are abbreviated according to accepted nomenclature. Regulatory interactions are summarized by logical connectives AND ($\wedge$), OR ($\vee$) and NOT ($\neg$).

ROS	Mitochondria $\vee$ $\neg$ATM $\vee$ $\neg$ FOXO3A $\vee$ $\neg$BMI1 $\vee$ $\neg$ TP53	Abbas et al. (2011); Chen et al. (2008); Gan et al. (2008); Ito et al. (2004, 2006); Juntilla et al. (2010); Kharas et al. (2010); Liu et al. (2009a); Ludin et al. (2014a); Miyamoto et al. (2007); Park et al. (2003); Rizo et al. (2009); Schuringa and Vellenga (2010); Suda et al. (2011a); Tothova and Gilliland (2007); Tothova et al. (2007); Yalcin et al. (2008); Liu et al. (2008)
Autophagy	FOXO3A $\wedge$ ROS $\wedge$ $\neg$ mTORC1	Ludin et al. (2014a); Martelli et al. (2010); Miyamoto et al. (2007); Mortensen et al. (2011); Warr et al. (2013a,b)
RAS	External cycling	Ema et al. (2000); Kaushansky (2009); Lee et al. (2020); Pietras et al. (2011a); Varghese et al. (2017)
ETS	RAS $\wedge$ $\neg$ MEF	Lacorazza et al. (2006); Liu et al. (2009b); Sashida et al. (2009)
MEF	RAS	Lacorazza et al. (2006); Liu et al. (2009b); Sashida et al. (2009)
GSK3β	$\neg$AKT	Martelli et al. (2010); Massagué (2004); Pietras et al. (2011a); Warr et al. (2011)
CTNNB1	$\neg$GSK3β	Dolnikov et al. (2014); Holmes et al. (2008); Huang et al. (2009); McCubrey et al. (2014); Robertson et al. (2018)
cMYC	CTNNB1 $\wedge$ $\neg$GSK3β	Maurer et al. (2014); McCubrey et al. (2014); Murphy et al. (2005); Robertson et al. (2018); Wilson et al. (2004a); Xu et al. (2009)
BMI1	cMYC $\vee$ (FOXO3A $\wedge$ ATM)	Guney et al. (2006); Ito et al. (2004, 2006); Jung et al. (2010); Kim et al. (2011); Park et al. (2003); Passegué et al. (2005); Rayess et al. (2012); Rizo et al. (2006); Suda et al. (2011a); Tothova et al. (2007); Yalcin et al. (2008)

Table S1: Boolean functions for the HSC model. Compounds are abbreviated according to accepted nomenclature. Regulatory interactions are summarized by logical connectives AND (∧), OR (∨) and NOT (¬).

MDM2	(TP53 ∨ MEF) ∧ ¬CDKN2D ∧ ¬ATM	Harris and Levine (2005); Haupt et al. (1997); Honda et al. (1997); Kastan et al. (2000); Kubbutat et al. (1997); Lacorazza et al. (2006); Liu et al. (2009b); Lowe and Sherr (2003); Maya et al. (2001); Meulmeester et al. (2005); Momand et al. (1992); Pant et al. (2012a); Perry (2010); Sashida et al. (2009); Sherr and Weber (2000); Sherr (2001); Shvarts et al. (1996); Suda et al. (2011a); Yalcin et al. (2008)
TP53	¬MDM2	Abbas et al. (2010, 2011); Haupt et al. (1997); Honda et al. (1997); Kubbutat et al. (1997); Liu et al. (2009b); Momand et al. (1992); Pant et al. (2012a); Perry (2010); Sherr (2001); Shvarts et al. (1996)
CDKN1C	External quiescence ∨ FOXO3A	Blank and Karlsson (2015); Miyamoto et al. (2007); Pietras et al. (2011a); Scandura et al. (2004); Yamazaki et al. (2006, 2007)
CDKN1A	(TP53 ∨ FOXO3A ∨ External quiescence ∨ GFI1) ∧ ¬cMYC	Asai et al. (2011); Baena et al. (2007); El-Deiry (1998); Eliasson et al. (2010); Hock et al. (2004); Massagué (2004); Murphy et al. (2005); Pietras et al. (2011a); Rizo et al. (2006); Tran et al. (2003); Vivanco and Sawyers (2002); Zeng et al. (2004)
CDKN1B	FOXO3A	Massagué (2004); Miyamoto et al. (2007); Tran et al. (2003); Vivanco and Sawyers (2002)
GFI1	TP53	Abbas et al. (2011); Liu et al. (2009b).
RB	¬CCND1 ∧ ¬CCNE1	Pietras et al. (2011a); Sherr and Roberts (1999); Tesio and Trumpp (2011a)
E2F	¬RB ∧ ¬ GFI1	Giacinti and Giordano (2006); Hock et al. (2004); Pietras et al. (2011a); Sherr and Roberts (1999); Tesio and Trumpp (2011a); Zeng et al. (2004)

Table S1: Boolean functions for the HSC model. Compounds are abbreviated according to accepted nomenclature. Regulatory interactions are summarized by logical connectives AND ($\wedge$), OR ($\vee$) and NOT ($\neg$).

CCND1	$\neg$CDKN2A $\wedge$ $\neg$ CDKN1C $\wedge$ cMYC	Bowie et al. (2007); Matsumoto et al. (2011a); Murphy et al. (2005); Passegué et al. (2005); Pietras et al. (2011a); Satoh et al. (2004); Sherr and Roberts (1999); Tesio and Trumpp (2011a); Zou et al. (2011a)
CCNE1	$\neg$CDKN1C $\wedge$ (($\neg$CDKN1A $\wedge$ $\neg$CDKN1B) $\vee$ CCND1) $\wedge$ E2F	Bowie et al. (2007); Cheng et al. (2000a,b, 2001); Foudi et al. (2009); Matsumoto et al. (2011a); Passegué et al. (2005); Pietras et al. (2011a); Scandura et al. (2004); Sherr and Roberts (1999); Sherr (2001); Tesio and Trumpp (2011a); van Os et al. (2007); Zou et al. (2011a)
S-phase	E2F $\wedge$ CCNE1	Hao et al. (2016); Pietras et al. (2011a); Sherr (2001)
AKT	PI3K	Martelli et al. (2010); Pietras et al. (2011a); Warr et al. (2011)
CDKN2A	(ETS $\vee$ ROS) $\wedge$ $\neg$BMI1	Ito et al. (2004, 2006); Jacobs et al. (1999); Lessard and Sauvageau (2003); Ludin et al. (2014a); Macleod (2008); Muller (2009); Park et al. (2003); Rizo et al. (2009); Sashida et al. (2009); Schuringa and Vellenga (2010); Serrano et al. (1997); Suda et al. (2011a)
CDKN2D	(E2F $\vee$ ROS) $\wedge$ $\neg$BMI1	Ito et al. (2004, 2006); Jacobs et al. (1999); Lessard and Sauvageau (2003); Lowe and Sherr (2003); Ludin et al. (2014a); Macleod (2008); Muller (2009); Park et al. (2003); Schuringa and Vellenga (2010); Serrano et al. (1997); Sherr (2001); Suda et al. (2011a); Zhu et al. (1999)
Pro-apoptotic proteins	ROS $\wedge$ TP53 $\wedge$ $\neg$AKT	Asai et al. (2011); Juntilla et al. (2010); Kennedy et al. (1999); Nii et al. (2012); Pant et al. (2012a); Redza-Dutordoir and Averill-Bates (2016); Song et al. (2005)

Table S1: Boolean functions for the HSC model. Compounds are abbreviated according to accepted nomenclature. Regulatory interactions are summarized by logical connectives AND ($\wedge$), OR ($\vee$) and NOT ($\neg$).

Anti-apoptotic proteins	(RAS $\vee$ External quiescence) $\wedge$ $\neg$GSK3β	Butler et al. (2010); Gerber et al. (2002); Hannum et al. (1994); Kollek et al. (2016); Maurer et al. (2014); McCubrey et al. (2014); Mojsa et al. (2014); Opferman et al. (2005); Qian et al. (2007); Varnum-Finney et al. (2000); Wang et al. (1998, 2013); Yoshimoto et al. (2009)
CYCS	Pro-apoptotic proteins $\wedge$ $\neg$Anti-apoptotic proteins	Domen (2000); Oguro and Iwama (2007); Orelio and Dzierzak (2007)
Apoptosis	CYCS $\wedge$ $\neg$AKT	Domen (2000); Juntilla et al. (2010); Kennedy et al. (1999); Oguro and Iwama (2007); Orelio and Dzierzak (2007); Song et al. (2005)
Senescence	(CDKN2A $\wedge$ ROS) $\vee$ (TP53 $\wedge$ ROS $\wedge$ CDKN1A)	Oguro and Iwama (2007); Shao et al. (2011); Warr et al. (2011)

1.2 Supplementary Table 2

Below, we provide the table containing the interpretation of our attractors with references for each compund described .

Table S2: Summary of analyzed attractors. Main nodes discussed in the text, final biological interpretation and references are reported for each obtained attractor.

**Attractor**	**Process**	**Phenotypical description**	**Associated HSC phenotype**	**References**
LT-HCS	Metabolism	inactive ROS	Quiescent	(Ludin et al., 2014b; Chen et al., 2008, 2009; Jang and Sharkis, 2007; Cabezas-Wallscheid et al., 2017)
	Metabolism	inactive mTORC1		(Ludin et al., 2014b; Chen et al., 2008, 2009; Jang and Sharkis, 2007; Baumgartner et al., 2018; Rodgers et al., 2014; Cabezas-Wallscheid et al., 2017)
	Metabolism	active FOXO3A		(Ludin et al., 2014b; Suda et al., 2011b; Miyamoto et al., 2007)

Table S2: Summary of analyzed attractors. Main nodes discussed in the text, final biological interpretation and references are reported for each obtained attractor.

	Cell cycle	inactive MYC		(Wilson et al., 2004b; Forsberg et al., 2005; Cabezas-Wallscheid et al., 2017)
	Cell cycle	active TP53		(Forsberg et al., 2005; Liu et al., 2009b; Jang and Sharkis, 2007; Pant et al., 2012b)
	Cell cycle	active CDKN1C		(Matsumoto et al., 2011b; Zou et al., 2011b; Tesio and Trumpp, 2011b; Umemoto et al., 2005; Chabanon et al., 2008)
	Cell cycle	active CDKN1A		(Forsberg et al., 2005; Pietras et al., 2011b)
	Cell cycle	active CDKN1B		(Forsberg et al., 2005; Pietras et al., 2011b)
	Cell cycle	active GFI1		(Liu et al., 2009b; Pant et al., 2012b)
	Cell cycle	inactive S-phase		(Pietras et al., 2011b; Orford and Scadden, 2008)
ST-HSC	Metabolism	active ROS	Activated HSC	(Ludin et al., 2014b; Chen et al., 2008, 2009; Jang and Sharkis, 2007; Cabezas-Wallscheid et al., 2017)
	Metabolism	active mTORC1		(Ludin et al., 2014b; Chen et al., 2008, 2009; Jang and Sharkis, 2007; Baumgartner et al., 2018; Rodgers et al., 2014; Cabezas-Wallscheid et al., 2017)
	Metabolism	inactive FOXO3A		(Ludin et al., 2014b; Suda et al., 2011b; Miyamoto et al., 2007)
	Cell cycle	active MYC		(Wilson et al., 2004b; Forsberg et al., 2005; Cabezas-Wallscheid et al., 2017)
	Cell cycle	inactive TP53		(Forsberg et al., 2005; Liu et al., 2009b; Jang and Sharkis, 2007; Pant et al., 2012b)
	Cell cycle	active CDKN1C		(Matsumoto et al., 2011b; Zou et al., 2011b; Tesio and Trumpp, 2011b; Umemoto et al., 2005; Chabanon et al., 2008)
	Cell cycle	inactive S-phase		(Pietras et al., 2011b; Orford and Scadden, 2008)
Cycling HSC	Cell cycle	active CCND1	Proliferating HSC	(Pietras et al., 2011b; Orford and Scadden, 2008)

Table S2: Summary of analyzed attractors. Main nodes discussed in the text, final biological interpretation and references are reported for each obtained attractor.

	Cell cycle	active CCNE1		(Pietras et al., 2011b; Orford and Scadden, 2008)
	Cell cycle	active S-phase		(Pietras et al., 2011b; Orford and Scadden, 2008)
Unstimulated	External stimuli	–	–	–

2 PROGRESSION FROM LT TO CYCLING HSC

In conclusion, we established a model able to faithfully recapitulate the regulation of HSC maintenance in the presence of external niche stimuli. We also simulated *in silico* the process of awakening of the HSC, giving a mechanistic and overall picture of the process. Further, we suggest a new general regulatory. We also considered the possibility that a highly quiescent HSC is directly driven to enter the cell cycle. Hence, it studied the impact of an external proliferative stimulation starting from the LT-HSC (Figure S1). This time the cascade required 13 time steps until an attractor was reached. The progression towards the attractor follows a similar cascade compared to the presence of both external factors (See main, Figure 3). Likewise, RAS/ATK signaling is stabilized (time step 1-9). Then, following the progression of the cascade of Figure 3 when external quiescence is removed, in the last three time steps, the phenotype switches towards further evolution of states leading to entry in the cell cycle. The lack of active cell cycle inhibitors leads to regulation of RB, CCND1, CCNE1, and finally to activation of the S-phase entry (time steps 10 to 14), thus, proliferation. Wiring responsible for the regulation of TP53 activity in HSCs. Moreover, we further validated our model by testing its predictive power on a variety of mouse models.

3 EVALUATION OF CYCLING BEHAVIOR FOR THE HSC PHENOTYPE

We evaluated if also our model could lead to a cycling attractor when considering a proliferating HSC. To test this hypothesis, we used the activation states of our nodes in the HSC cycling attractor to simulate the attractor search of the (Fauré et al., 2006) model. Here, we considered the status of CCND1 (CycD in the (Fauré et al., 2006) model, active) , RB (inactive), CCNE1 (CycD in the (Fauré et al., 2006) model, active), E2F (active), and CDKN1B (p27 in the (Fauré et al., 2006), inactive). The remaining nodes not considered in our model are set to zero. The cascade in Figure S4 shows that the activation states lead to a cyclic attractor.

4 FURTHER SIMULATIONS ON THE TP53 REGULATORY MECHANISM

We further investigated the validity of our general mechanism of TP53 regulation. First, we simulated if in the absence of external cycling signals, we can still obtain the same TP53 regulation by either constitutive activation of FOXO3A/ATM axis or RAS/PI3K. Results are presented in Figure S2. Here, in LT-HSC in absence of external cycling stimuli, constitutive active FOXO3A/ATM activates TP53. On the other hand, again in absence of external cycling stimuli, constitutive RAS/PI3K downregulates TP53 in ST-HSC.

Once tested that these two axes are responsible for TP53 regulation, we also investigated the effect of loss of regulation mutants. We modified the original interactions of the HSC network by: excluding the destabilization of ATM on MDM2, excluding the stabilization of MEF on MDM2, or both. Then, we performed once more the exhaustive attractor search. The results are shown in Figure S3. We could show that loss of interactions on the TP53 regulation causes a general destabilization of the attractors that assume cyclic behavior. Furthermore, the effects are additive. Loss of ATM interaction causes perturbation of the LT-HSC attractor, MEF loss of interaction causes perturbation of the ST and cycling HSC attractors, and the combined loss perturbs all attractors.

5 FIGURES

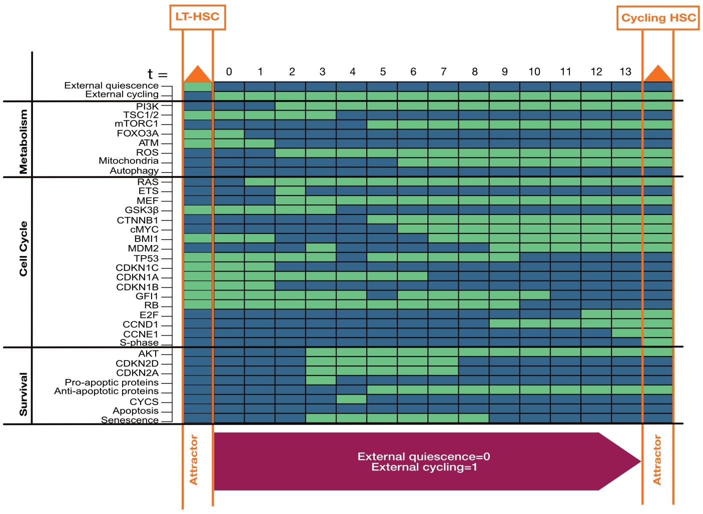

Figure S1: Proliferative external stimulation on LT-HSCs. The figure shows progression from LT to cycling HSC depending on activation of external cycling stimulation. Attractors are highlighted in orange in the progression. Activated external cycling stimulation and absence of external quiescence stimulation are depicted in magenta box below. Components of the model are listed on the left separated into assigned pathways. The state of each component is depicted by colored rectangles. Green indicates active components; blue indicates inactive ones.

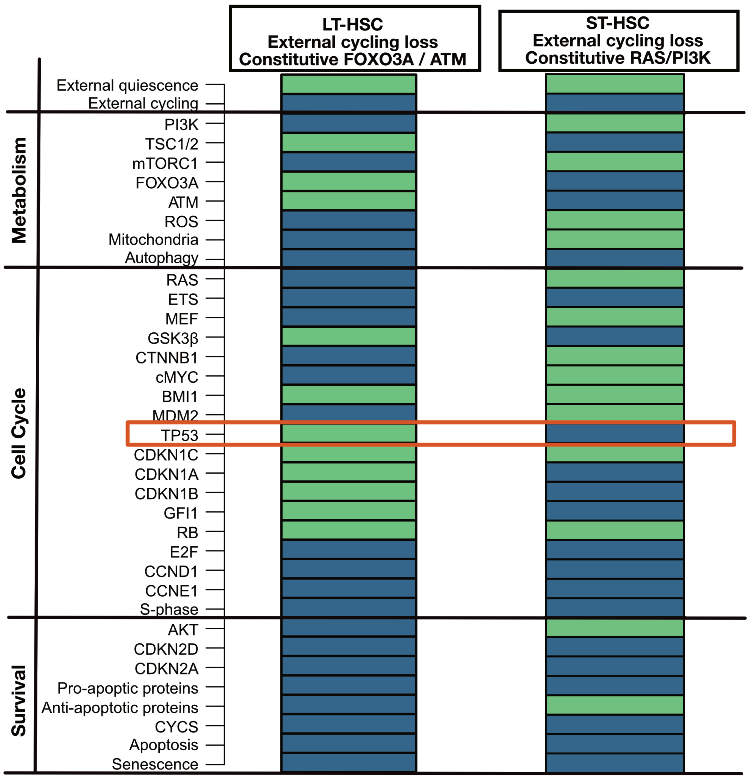

Figure S2: Loss of external cycling stimuli and constitutive FOXO3A/ATM or RAS/PI3K maintains TP53 regulation. LT-HSC (on the left) and ST-HSC (on the right) are presented. In both cases external cycling stimuli have been downregulated. On the LT-HSC attractor, constitutive activation of FOXO3A/ATM still causes an upregulation of TP53. On the ST-HSC attractor, constitutive activation of RAS/PI3K causes downregulation of TP53. Components of the model are listed on the left separated into assigned pathways. Green indicates active components; blue indicates inactive ones.

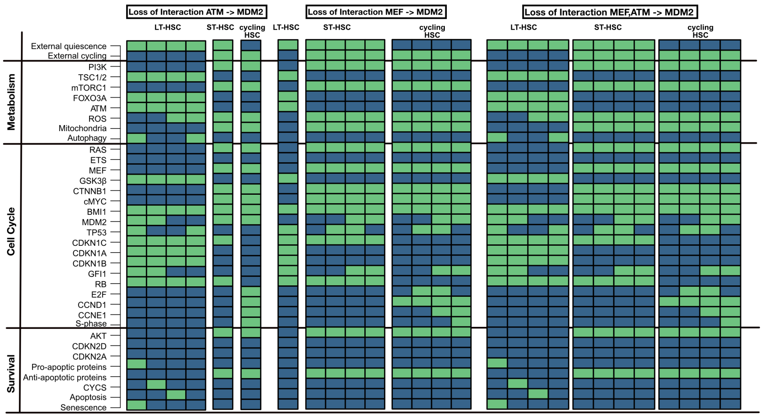

Figure S3: Loss of interaction mutants show altered attractors for the HSC model. Three loss of interaction networks have been simulated for attractor search. On the left, mutant for loss of ATM regulation causes cyclic LT-HSC attractor with perturbed TP53 regulation. On the central panel, mutant for loss of regulation of MEF causes cycling ST-HSC and cycling HSC attractors. On the right, mutant for both loss of interaction of ATM and MEF causes perturbation of all attractors in an additive way. Components of the model are listed on the left separated into assigned pathways. Green indicates active components; blue indicates inactive ones.

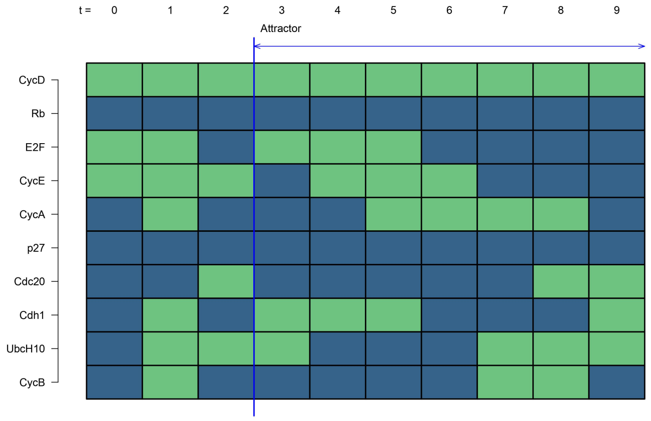

Figure S4: The the cycling HSC phenotype can lead to a cyclic behavior. The activity of CCND1 (CycD),Rb, E2F, CCNE1 (CycE), and P27 were used as start states for attractor search in the cell cycle model from (Fauré et al., 2006). It is shown that the starting states lead to a cyclic attractor. The state of each component is depicted by colored rectangles. Green indicates active components; blue indicates inactive ones.

5.0.1 Single-Node Perturbation Experiments

In the following, we simulated each of the 84 possible single-node interventions which is possible over all 37 nodes in the proposed network.

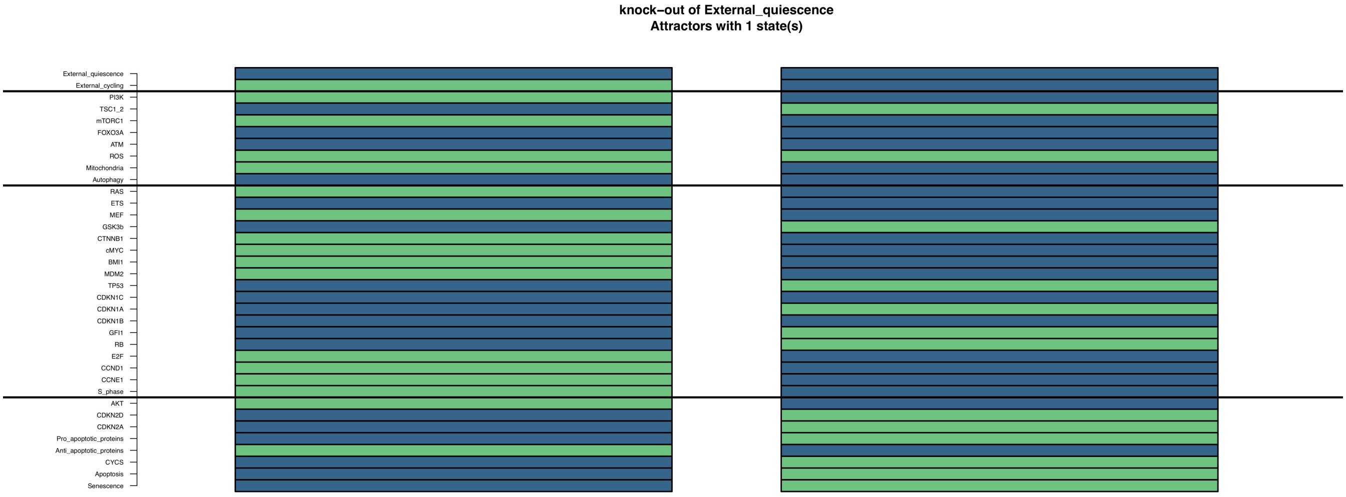

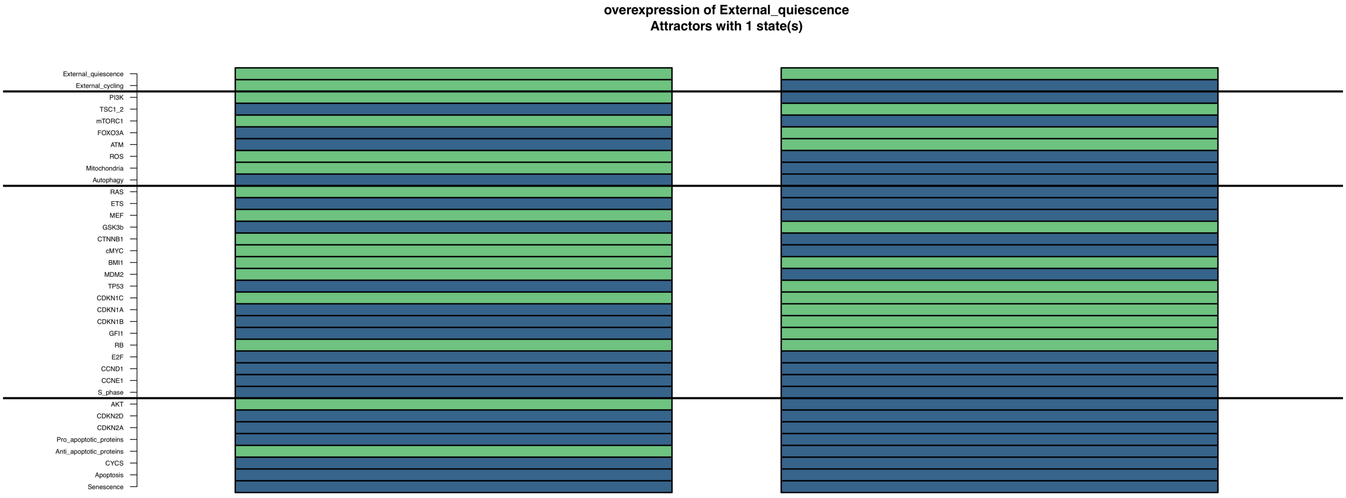

knock–out of External_cycling
Attractors with 1 state(s)

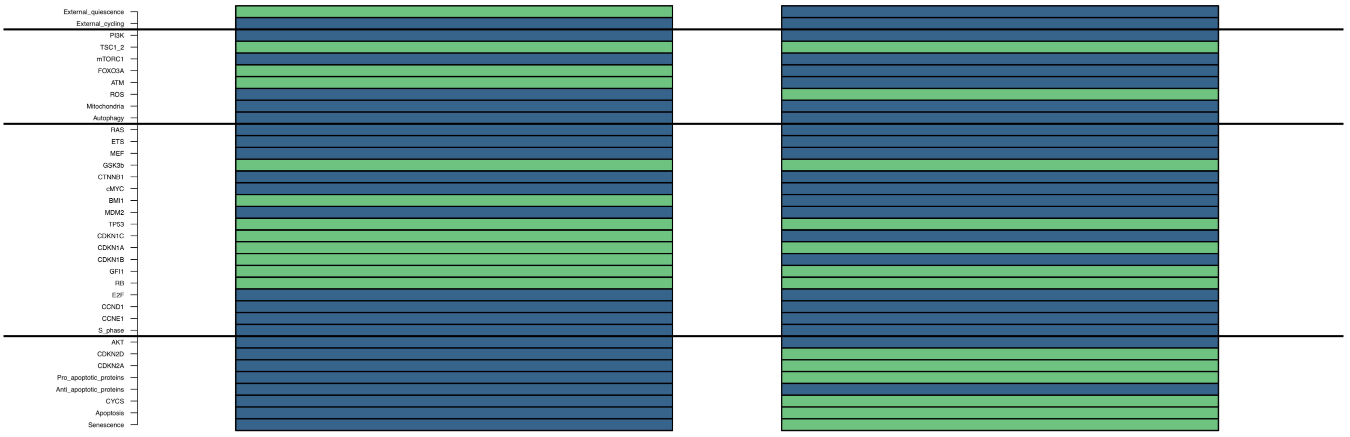

overexpression of External_cycling
Attractors with 1 state(s)

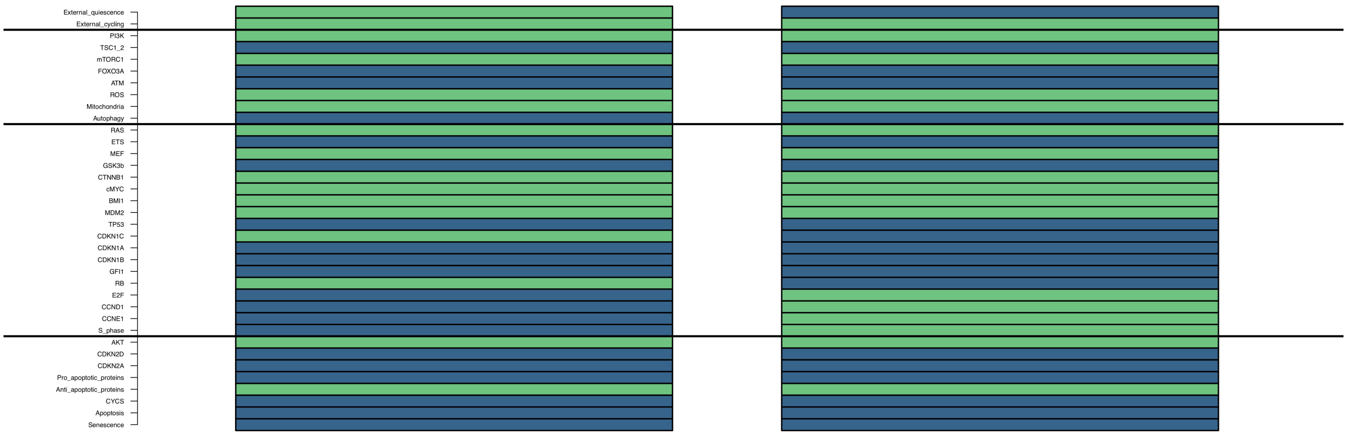

knock–out of PI3K
Attractors with 1 state(s)

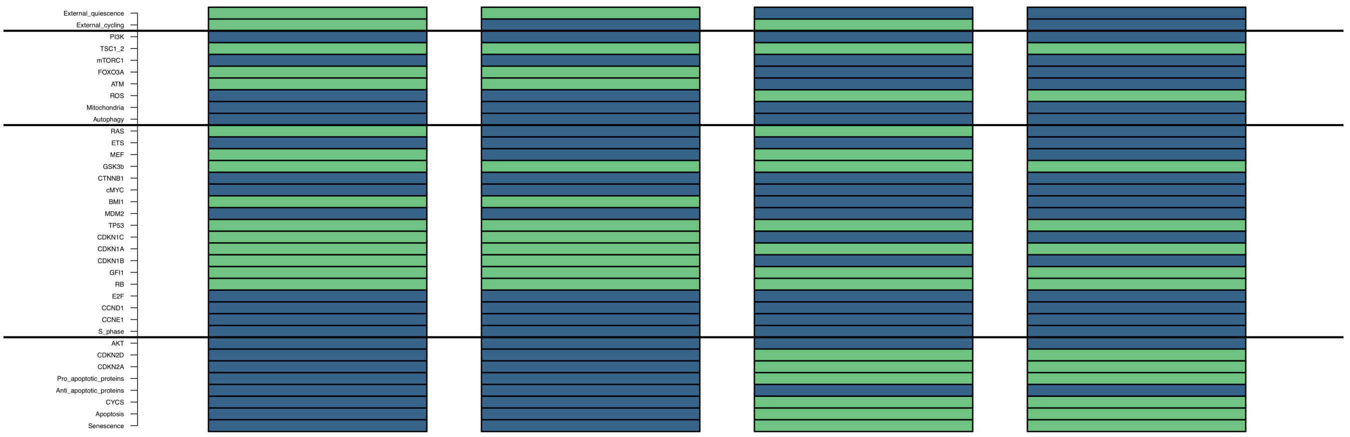

overexpression of PI3K
Attractors with 1 state(s)

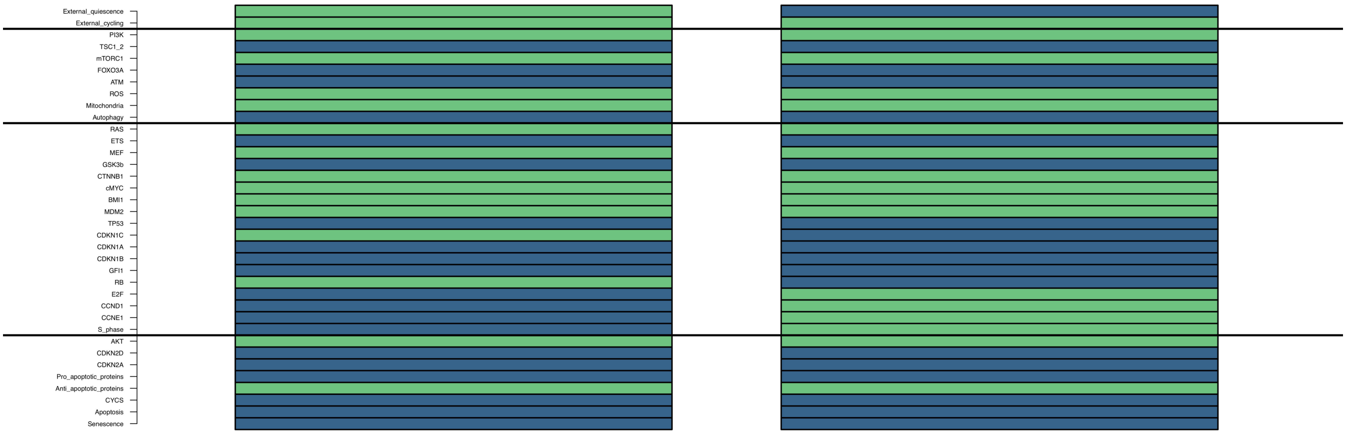

overexpression of PI3K
Attractors with 4 state(s)

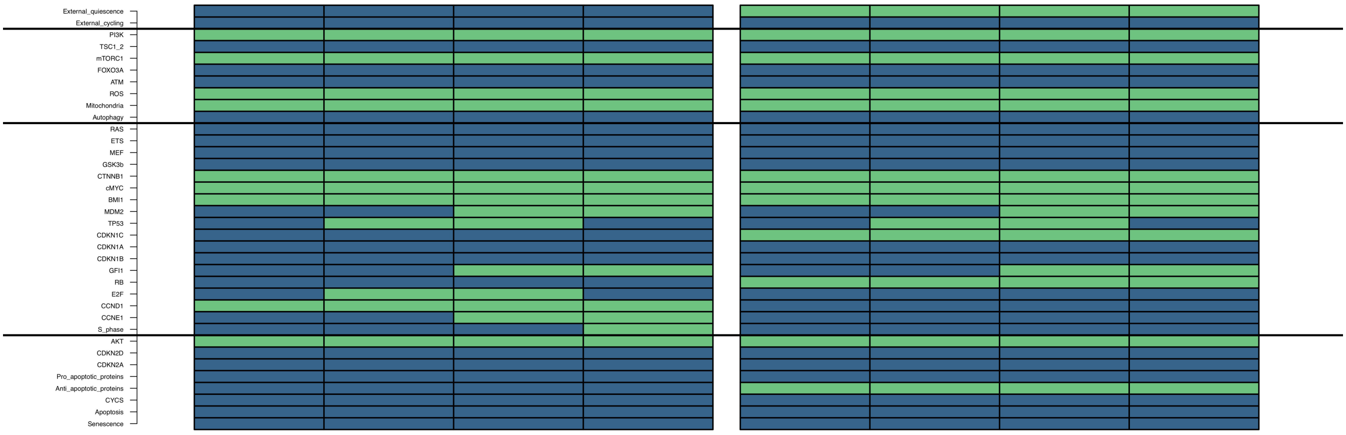

knock–out of TSC1_2
Attractors with 1 state(s)

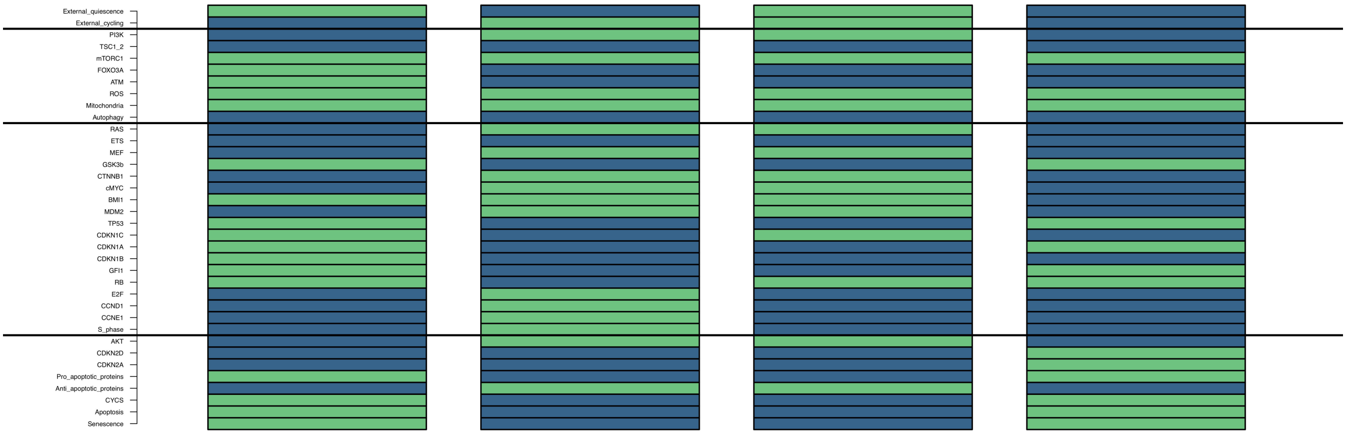

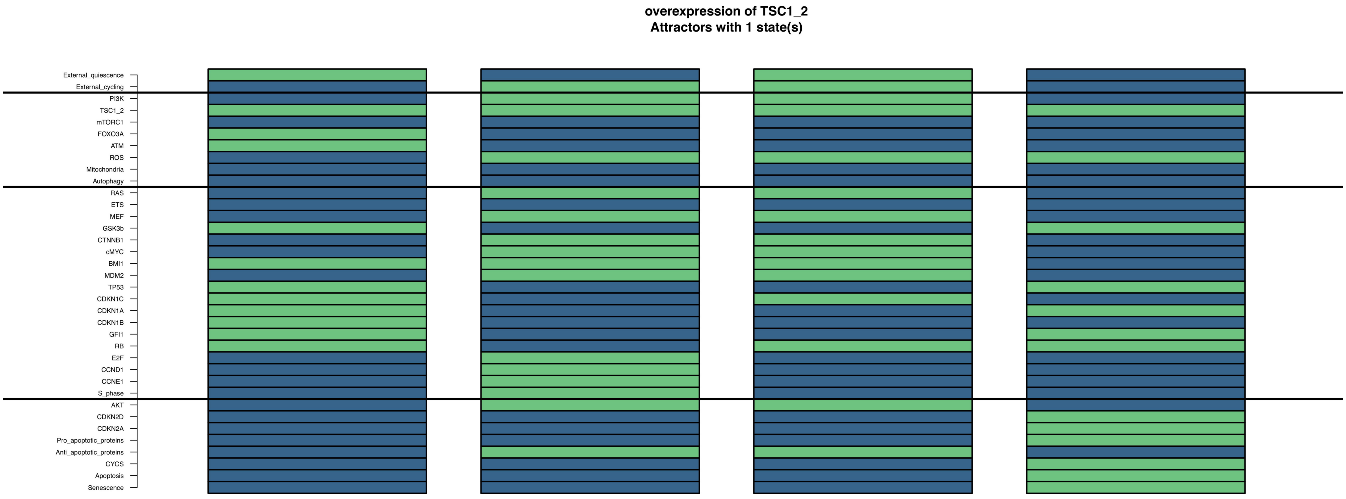
overexpression of TSC1_2
Attractors with 1 state(s)
External_quiescence
External_cycling
PI3K
TSC1_2
mTORC1
FOXO3A
ATM
ROS
Mitochondria
Autophagy
RAS
ETS
MEF
GSK3b
CTNNB1
cMYC
BMI1
MDM2
TP53
CDKN1C
CDKN1A
CDKN1B
GFI1
RB
E2F
CCND1
CCNE1
S_phase
AKT
CDKN2D
CDKN2A
Pro_apoptotic_proteins
Anti_apoptotic_proteins
CYCS
Apoptosis
Senescence

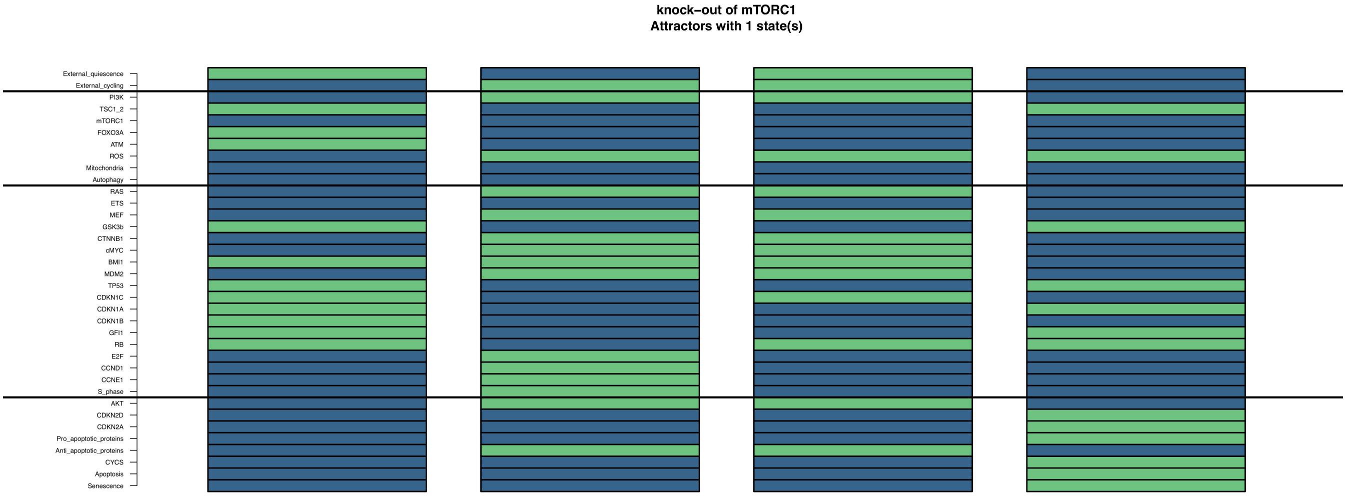
knock–out of mTORC1
Attractors with 1 state(s)
External_quiescence
External_cycling
PI3K
TSC1_2
mTORC1
FOXO3A
ATM
ROS
Mitochondria
Autophagy
RAS
ETS
MEF
GSK3b
CTNNB1
cMYC
BMI1
MDM2
TP53
CDKN1C
CDKN1A
CDKN1B
GFI1
RB
E2F
CCND1
CCNE1
S_phase
AKT
CDKN2D
CDKN2A
Pro_apoptotic_proteins
Anti_apoptotic_proteins
CYCS
Apoptosis
Senescence

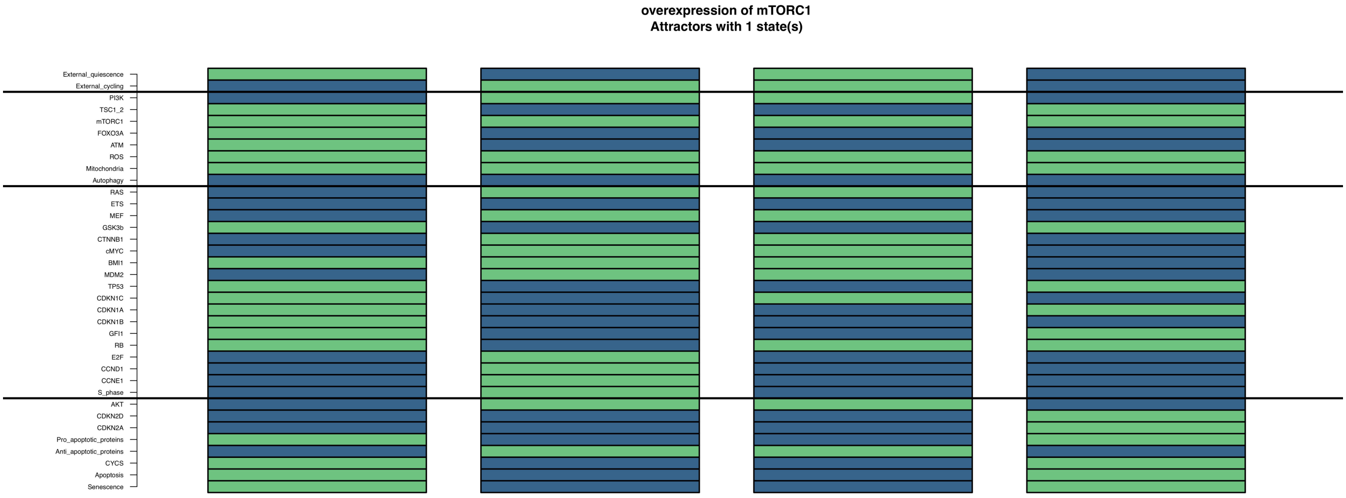
overexpression of mTORC1
Attractors with 1 state(s)
External_quiescence
External_cycling
PI3K
TSC1_2
mTORC1
FOXO3A
ATM
ROS
Mitochondria
Autophagy
RAS
ETS
MEF
GSK3b
CTNNB1
cMYC
BMI1
MDM2
TP53
CDKN1C
CDKN1A
CDKN1B
GFI1
RB
E2F
CCND1
CCNE1
S_phase
AKT
CDKN2D
CDKN2A
Pro_apoptotic_proteins
Anti_apoptotic_proteins
CYCS
Apoptosis
Senescence

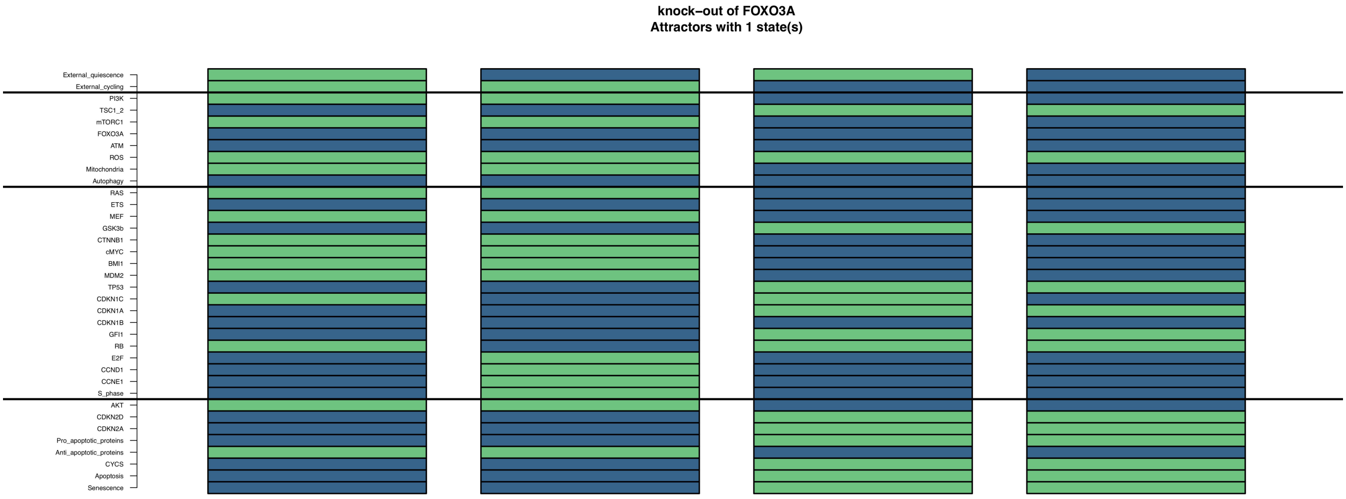
knock–out of FOXO3A
Attractors with 1 state(s)
External_quiescence
External_cycling
PI3K
TSC1_2
mTORC1
FOXO3A
ATM
ROS
Mitochondria
Autophagy
RAS
ETS
MEF
GSK3b
CTNNB1
cMYC
BMI1
MDM2
TP53
CDKN1C
CDKN1A
CDKN1B
GFI1
RB
E2F
CCND1
CCNE1
S_phase
AKT
CDKN2D
CDKN2A
Pro_apoptotic_proteins
Anti_apoptotic_proteins
CYCS
Apoptosis
Senescence

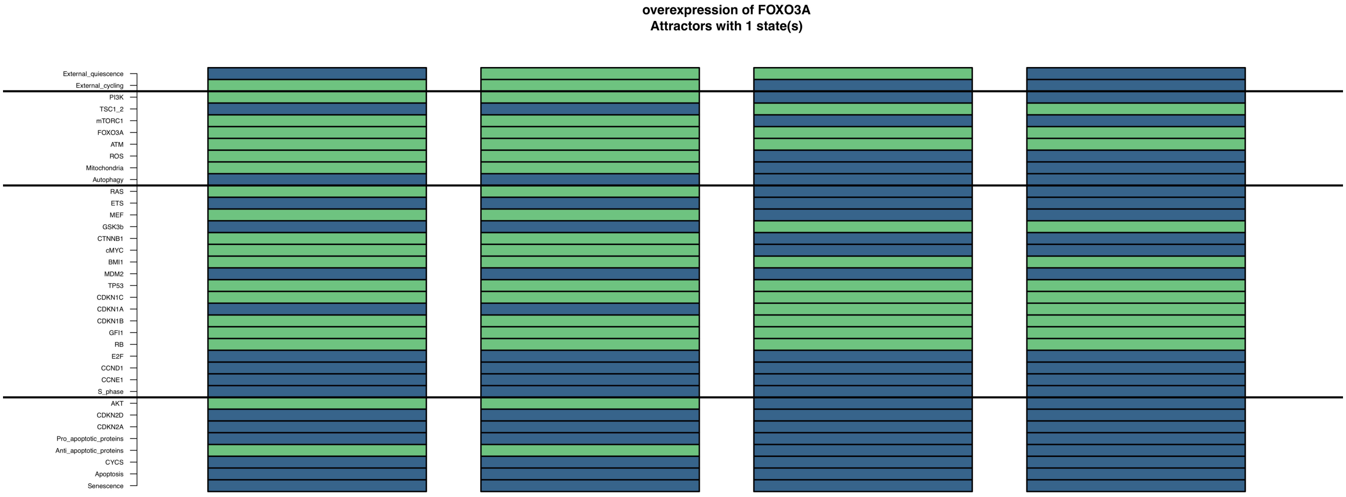
overexpression of FOXO3A
Attractors with 1 state(s)
External_quiescence
External_cycling
PI3K
TSC1_2
mTORC1
FOXO3A
ATM
ROS
Mitochondria
Autophagy
RAS
ETS
MEF
GSK3b
CTNNB1
cMYC
BMI1
MDM2
TP53
CDKN1C
CDKN1A
CDKN1B
GFI1
RB
E2F
CCND1
CCNE1
S_phase
AKT
CDKN2D
CDKN2A
Pro_apoptotic_proteins
Anti_apoptotic_proteins
CYCS
Apoptosis
Senescence

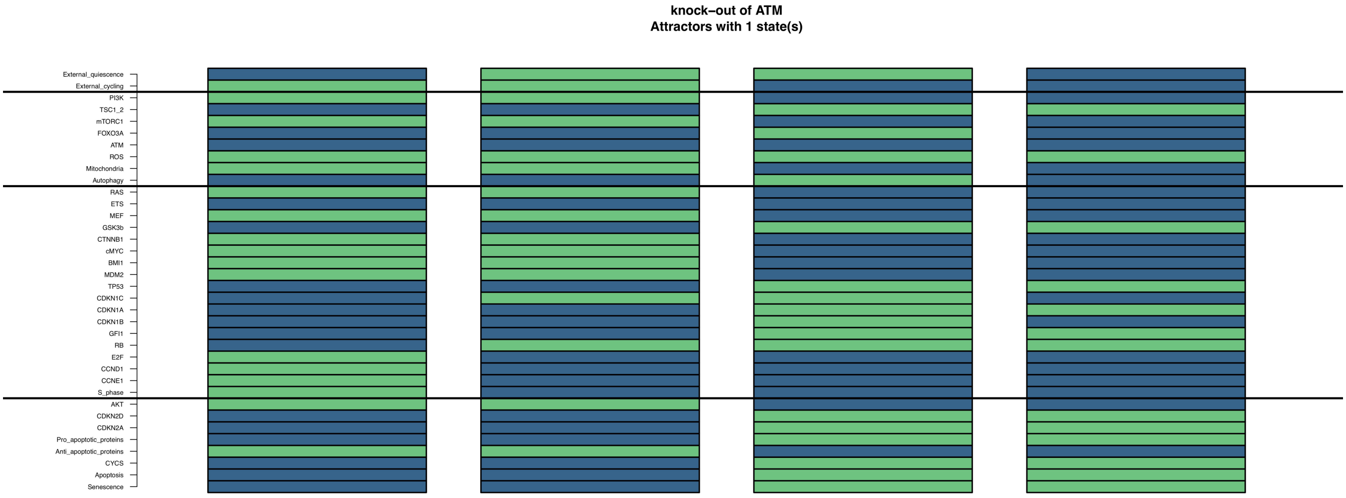
knock–out of ATM
Attractors with 1 state(s)
External_quiescence
External_cycling
PI3K
TSC1_2
mTORC1
FOXO3A
ATM
ROS
Mitochondria
Autophagy
RAS
ETS
MEF
GSK3b
CTNNB1
cMYC
BMI1
MDM2
TP53
CDKN1C
CDKN1A
CDKN1B
GFI1
RB
E2F
CCND1
CCNE1
S_phase
AKT
CDKN2D
CDKN2A
Pro_apoptotic_proteins
Anti_apoptotic_proteins
CYCS
Apoptosis
Senescence

overexpression of ATM
Attractors with 1 state(s)

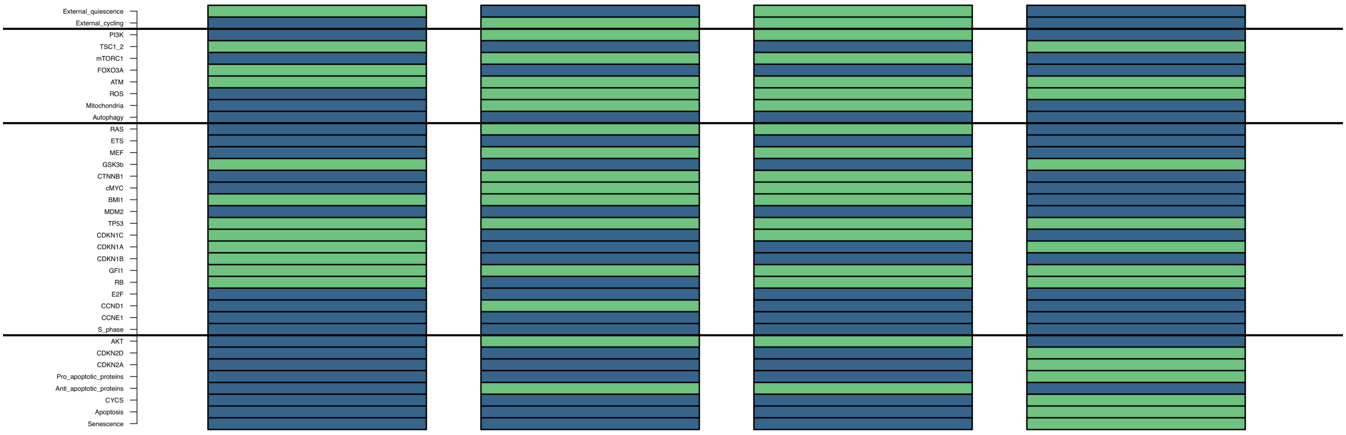

knock–out of ROS
Attractors with 1 state(s)

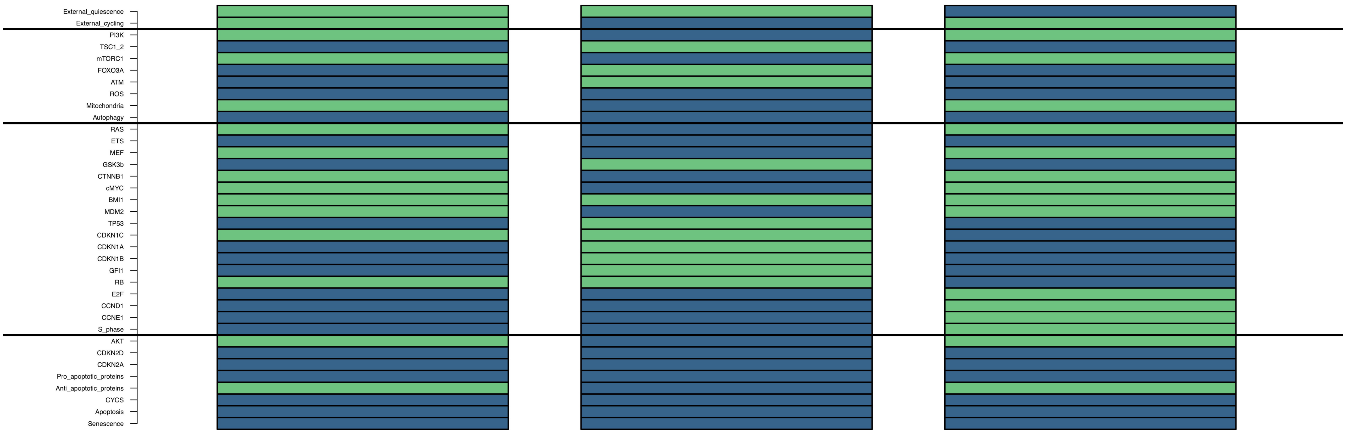

knock–out of ROS
Attractors with 4 state(s)

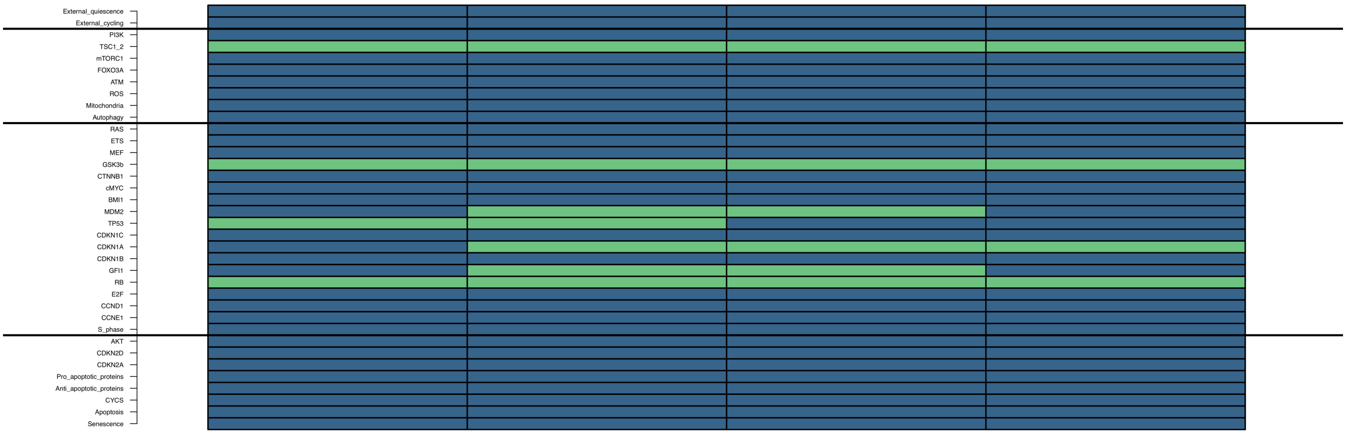

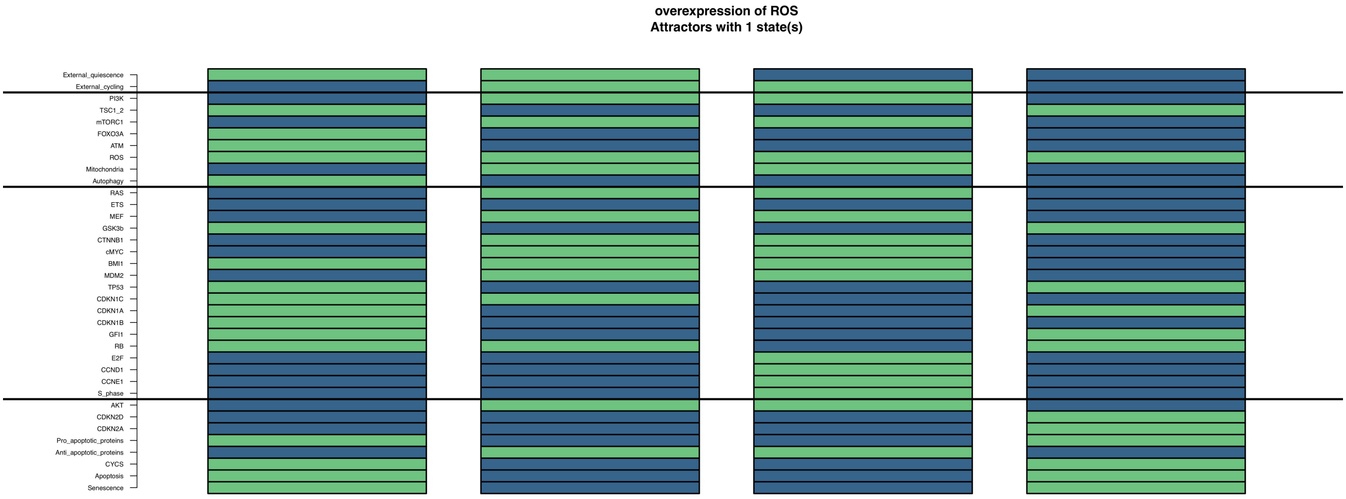
overexpression of ROS
Attractors with 1 state(s)
External_quiescence
External_cycling
PI3K
TSC1_2
mTORC1
FOXO3A
ATM
ROS
Mitochondria
Autophagy
RAS
ETS
MEF
GSK3b
CTNNB1
cMYC
BMI1
MDM2
TP53
CDKN1C
CDKN1A
CDKN1B
GFI1
RB
E2F
CCND1
CCNE1
S_phase
AKT
CDKN2D
CDKN2A
Pro_apoptotic_proteins
Anti_apoptotic_proteins
CYCS
Apoptosis
Senescence

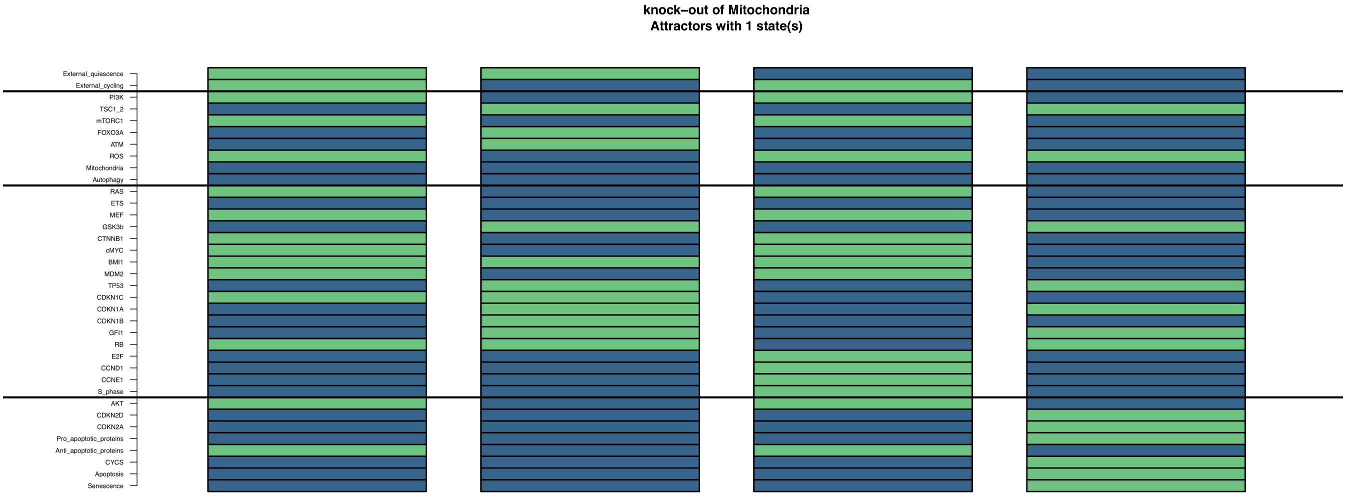
knock–out of Mitochondria
Attractors with 1 state(s)
External_quiescence
External_cycling
PI3K
TSC1_2
mTORC1
FOXO3A
ATM
ROS
Mitochondria
Autophagy
RAS
ETS
MEF
GSK3b
CTNNB1
cMYC
BMI1
MDM2
TP53
CDKN1C
CDKN1A
CDKN1B
GFI1
RB
E2F
CCND1
CCNE1
S_phase
AKT
CDKN2D
CDKN2A
Pro_apoptotic_proteins
Anti_apoptotic_proteins
CYCS
Apoptosis
Senescence

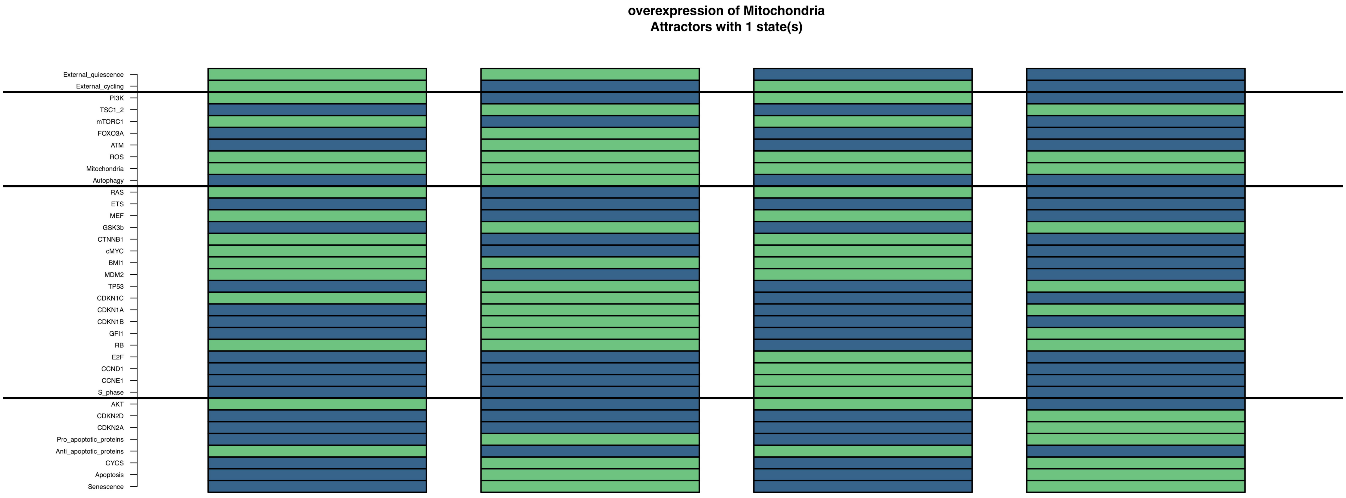
overexpression of Mitochondria
Attractors with 1 state(s)
External_quiescence
External_cycling
PI3K
TSC1_2
mTORC1
FOXO3A
ATM
ROS
Mitochondria
Autophagy
RAS
ETS
MEF
GSK3b
CTNNB1
cMYC
BMI1
MDM2
TP53
CDKN1C
CDKN1A
CDKN1B
GFI1
RB
E2F
CCND1
CCNE1
S_phase
AKT
CDKN2D
CDKN2A
Pro_apoptotic_proteins
Anti_apoptotic_proteins
CYCS
Apoptosis
Senescence

knock–out of Autophagy
Attractors with 1 state(s)

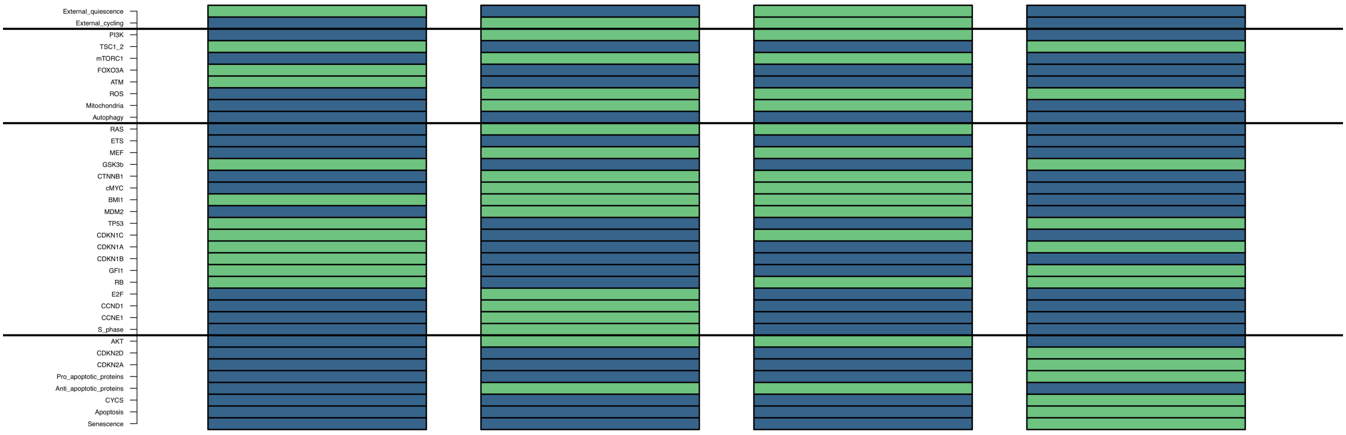

overexpression of Autophagy
Attractors with 1 state(s)

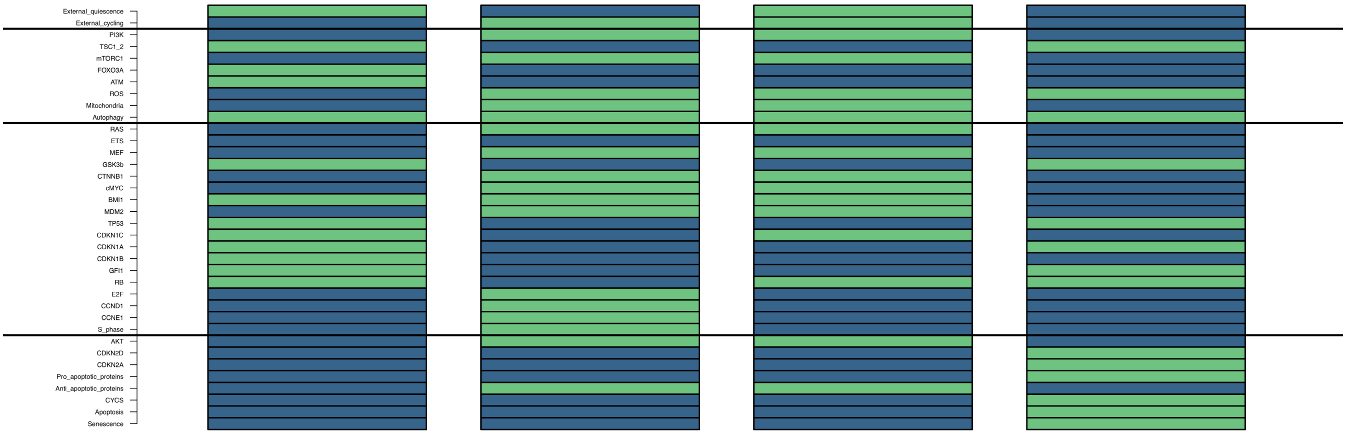

knock–out of RAS
Attractors with 1 state(s)

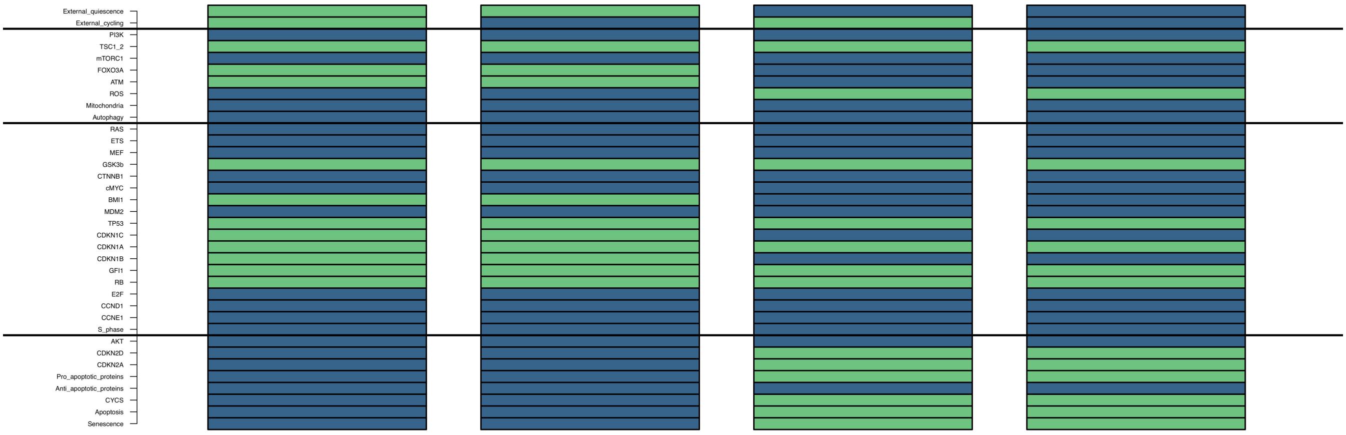

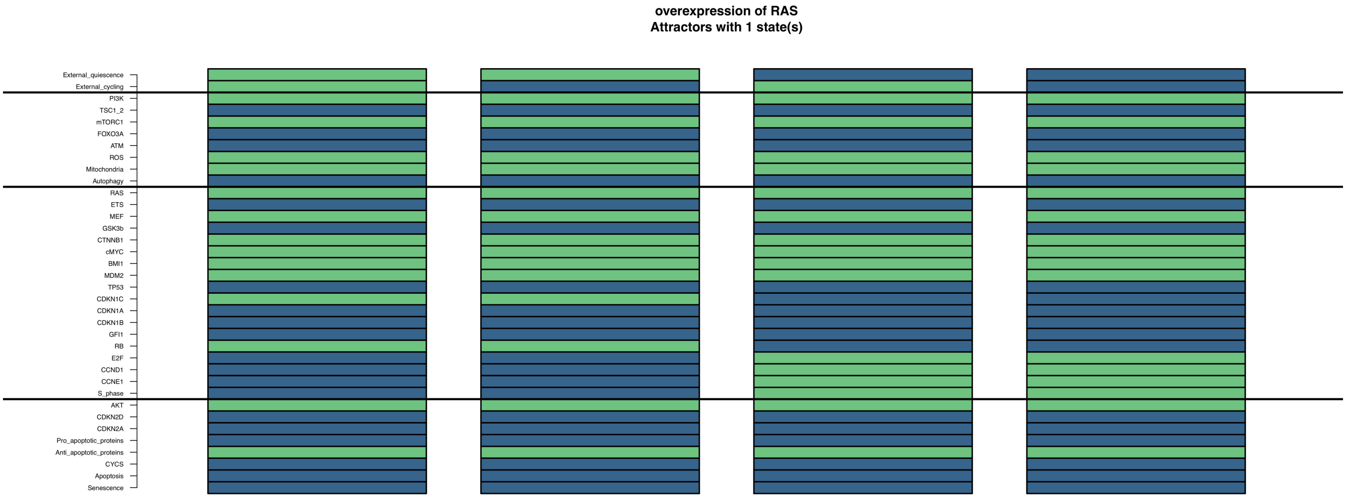
overexpression of RAS
Attractors with 1 state(s)
External_quiescence
External_cycling
PI3K
TSC1_2
mTORC1
FOXO3A
ATM
ROS
Mitochondria
Autophagy
RAS
ETS
MEF
GSK3b
CTNNB1
cMYC
BMI1
MDM2
TP53
CDKN1C
CDKN1A
CDKN1B
GFI1
RB
E2F
CCND1
CCNE1
S_phase
AKT
CDKN2D
CDKN2A
Pro_apoptotic_proteins
Anti_apoptotic_proteins
CYCS
Apoptosis
Senescence

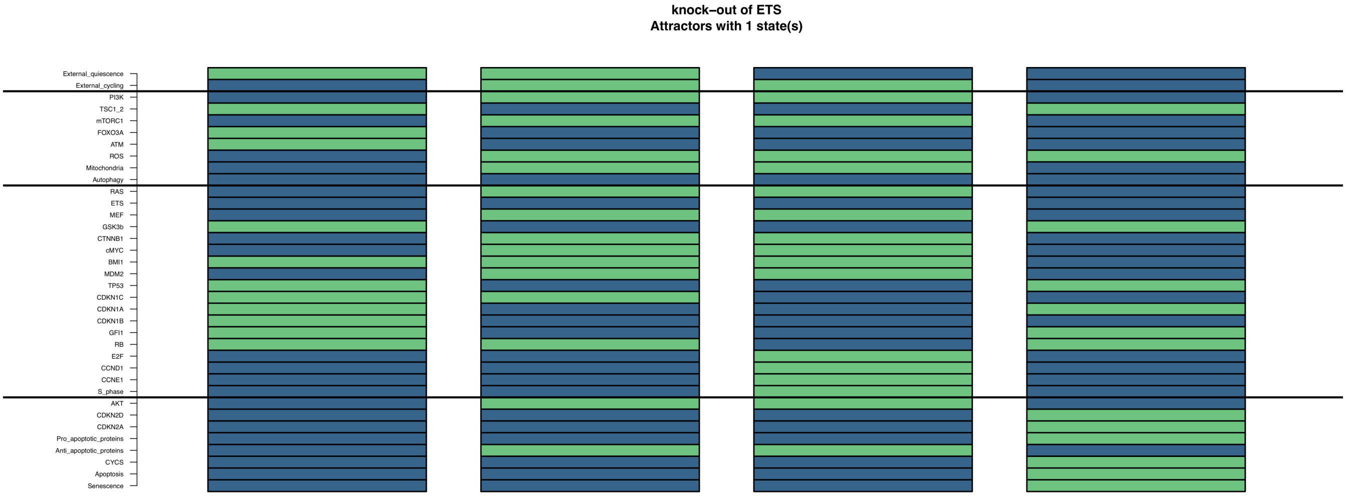
knock–out of ETS
Attractors with 1 state(s)
External_quiescence
External_cycling
PI3K
TSC1_2
mTORC1
FOXO3A
ATM
ROS
Mitochondria
Autophagy
RAS
ETS
MEF
GSK3b
CTNNB1
cMYC
BMI1
MDM2
TP53
CDKN1C
CDKN1A
CDKN1B
GFI1
RB
E2F
CCND1
CCNE1
S_phase
AKT
CDKN2D
CDKN2A
Pro_apoptotic_proteins
Anti_apoptotic_proteins
CYCS
Apoptosis
Senescence

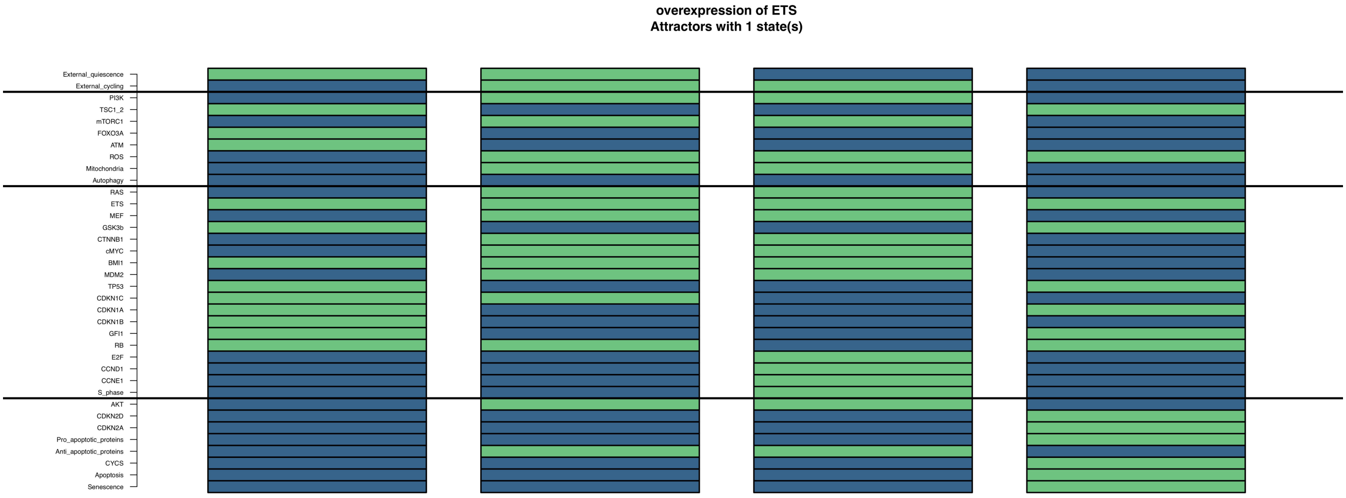
overexpression of ETS
Attractors with 1 state(s)
External_quiescence
External_cycling
PI3K
TSC1_2
mTORC1
FOXO3A
ATM
ROS
Mitochondria
Autophagy
RAS
ETS
MEF
GSK3b
CTNNB1
cMYC
BMI1
MDM2
TP53
CDKN1C
CDKN1A
CDKN1B
GFI1
RB
E2F
CCND1
CCNE1
S_phase
AKT
CDKN2D
CDKN2A
Pro_apoptotic_proteins
Anti_apoptotic_proteins
CYCS
Apoptosis
Senescence

knock–out of MEF
Attractors with 1 state(s)

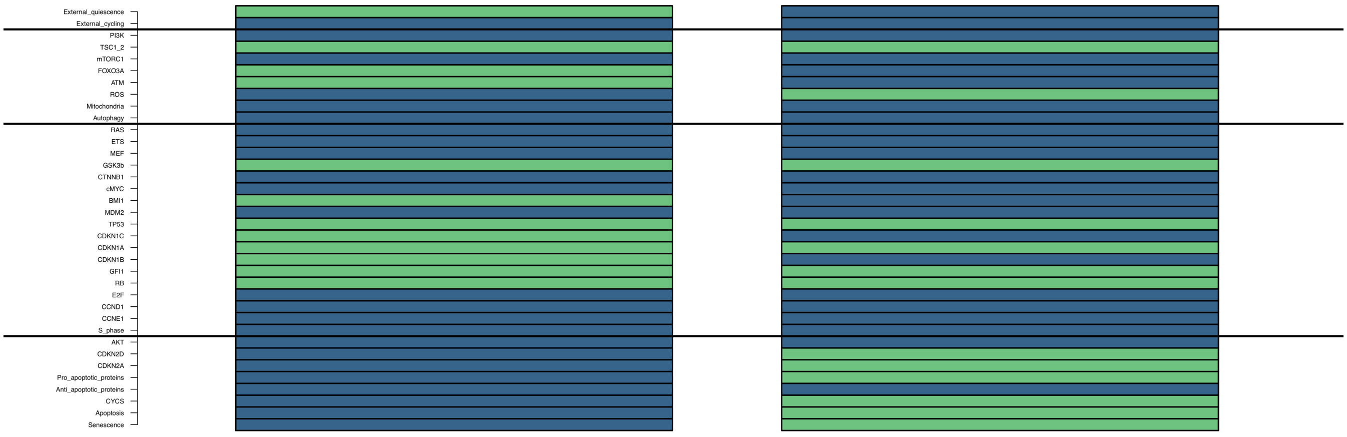

knock–out of MEF
Attractors with 4 state(s)

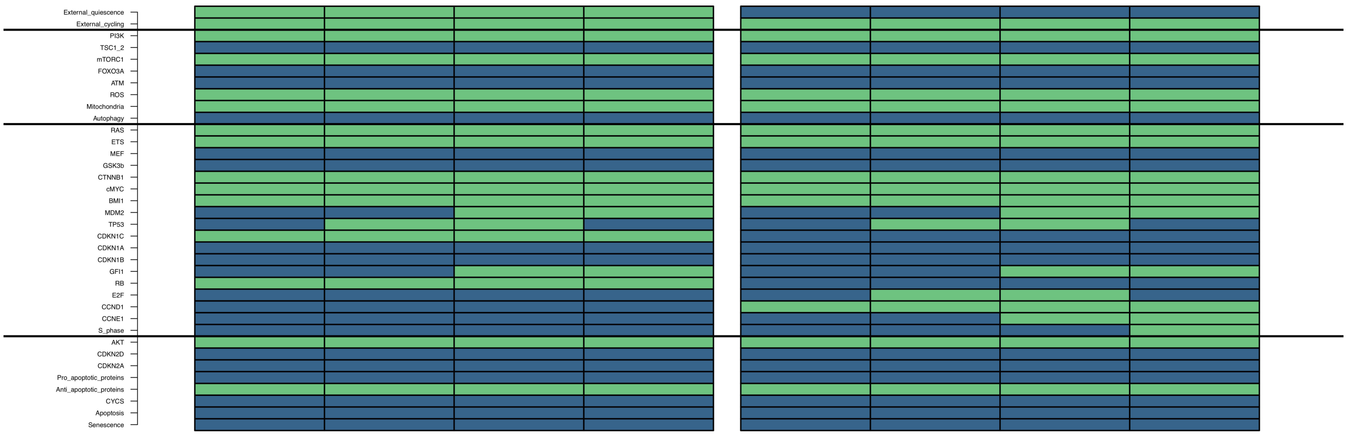

overexpression of MEF
Attractors with 1 state(s)

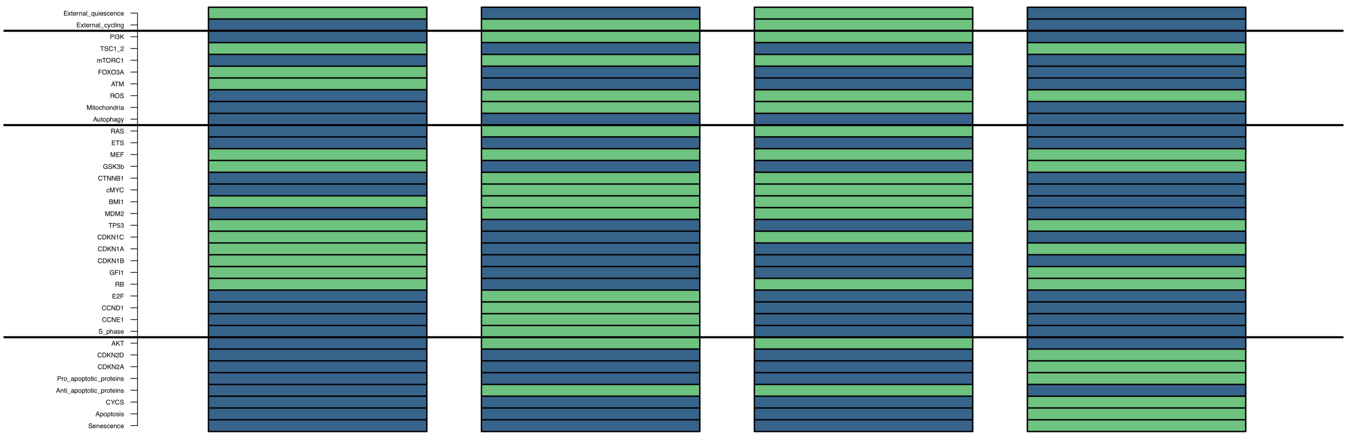

knock–out of GSK3b
Attractors with 1 state(s)

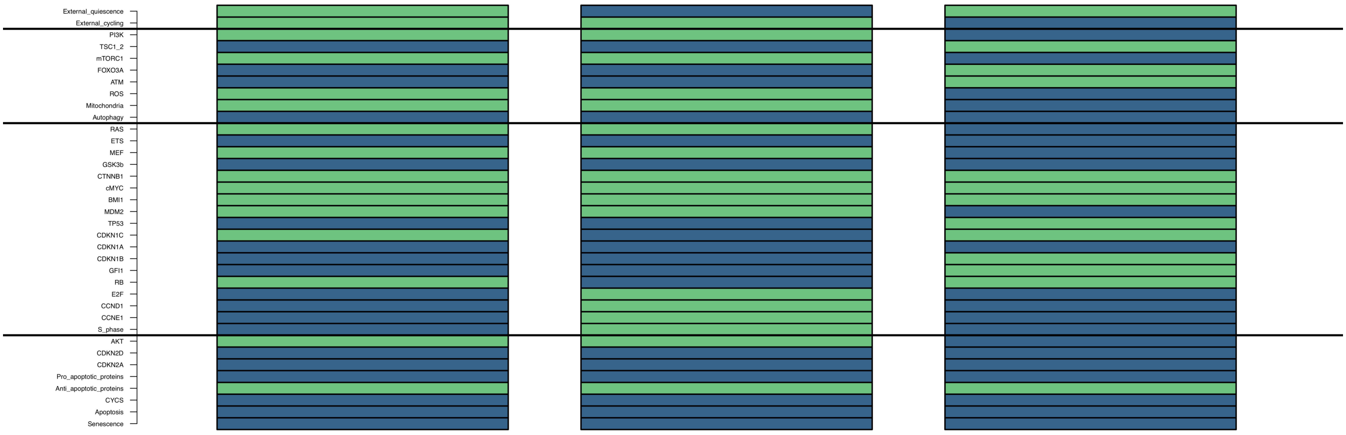

knock–out of GSK3b
Attractors with 4 state(s)

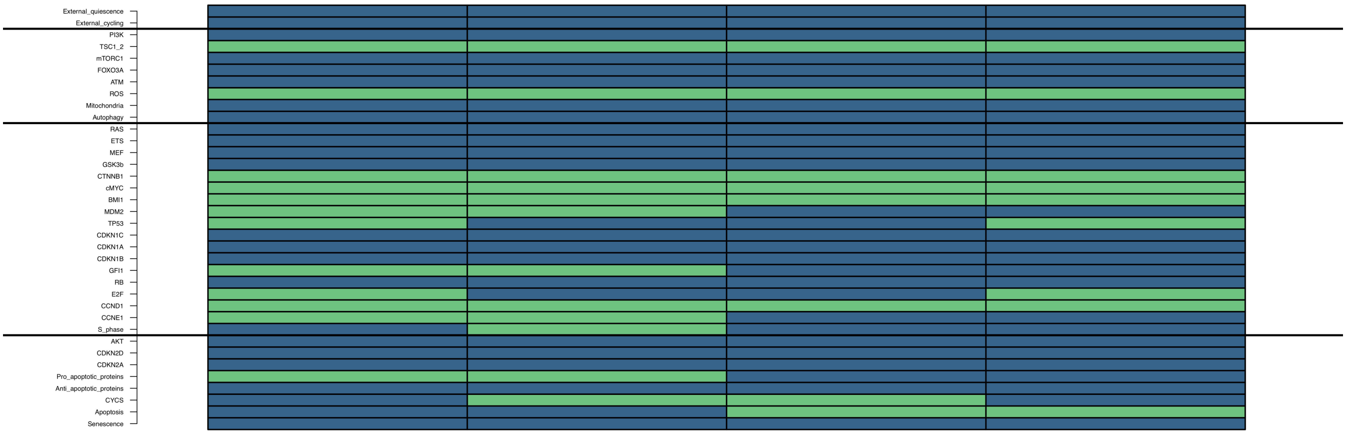

overexpression of GSK3b
Attractors with 1 state(s)

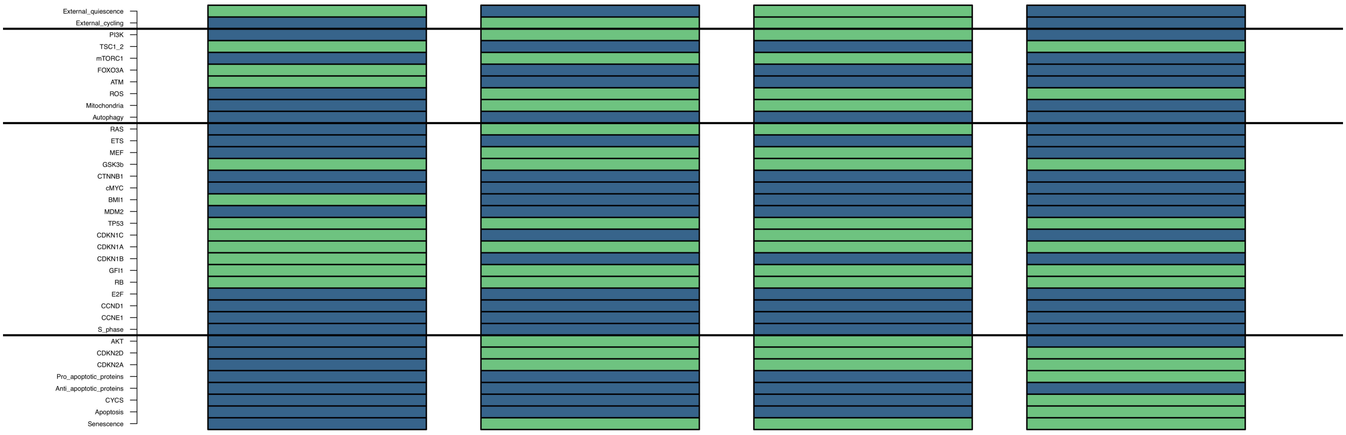

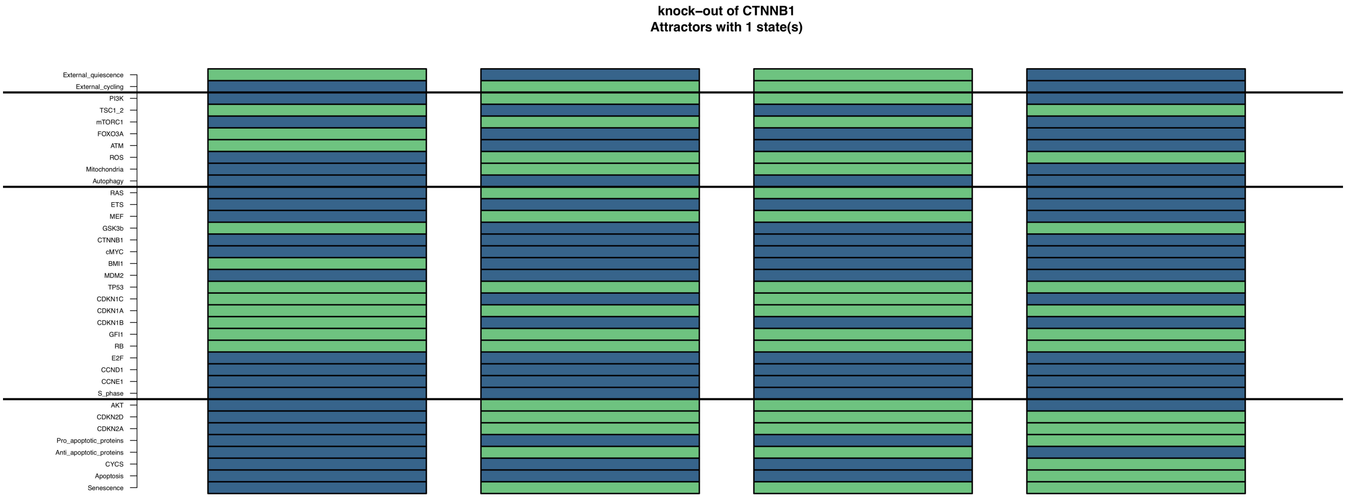
knock–out of CTNNB1
Attractors with 1 state(s)
External_quiescence
External_cycling
PI3K
TSC1_2
mTORC1
FOXO3A
ATM
ROS
Mitochondria
Autophagy
RAS
ETS
MEF
GSK3b
CTNNB1
cMYC
BMI1
MDM2
TP53
CDKN1C
CDKN1A
CDKN1B
GFI1
RB
E2F
CCND1
CCNE1
S_phase
AKT
CDKN2D
CDKN2A
Pro_apoptotic_proteins
Anti_apoptotic_proteins
CYCS
Apoptosis
Senescence

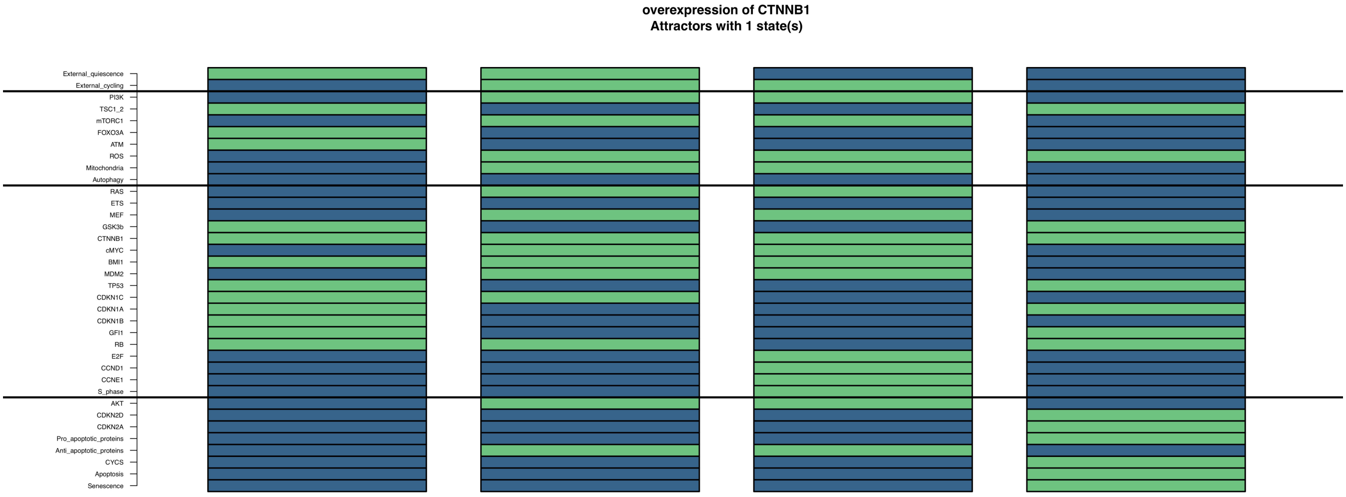
overexpression of CTNNB1
Attractors with 1 state(s)
External_quiescence
External_cycling
PI3K
TSC1_2
mTORC1
FOXO3A
ATM
ROS
Mitochondria
Autophagy
RAS
ETS
MEF
GSK3b
CTNNB1
cMYC
BMI1
MDM2
TP53
CDKN1C
CDKN1A
CDKN1B
GFI1
RB
E2F
CCND1
CCNE1
S_phase
AKT
CDKN2D
CDKN2A
Pro_apoptotic_proteins
Anti_apoptotic_proteins
CYCS
Apoptosis
Senescence

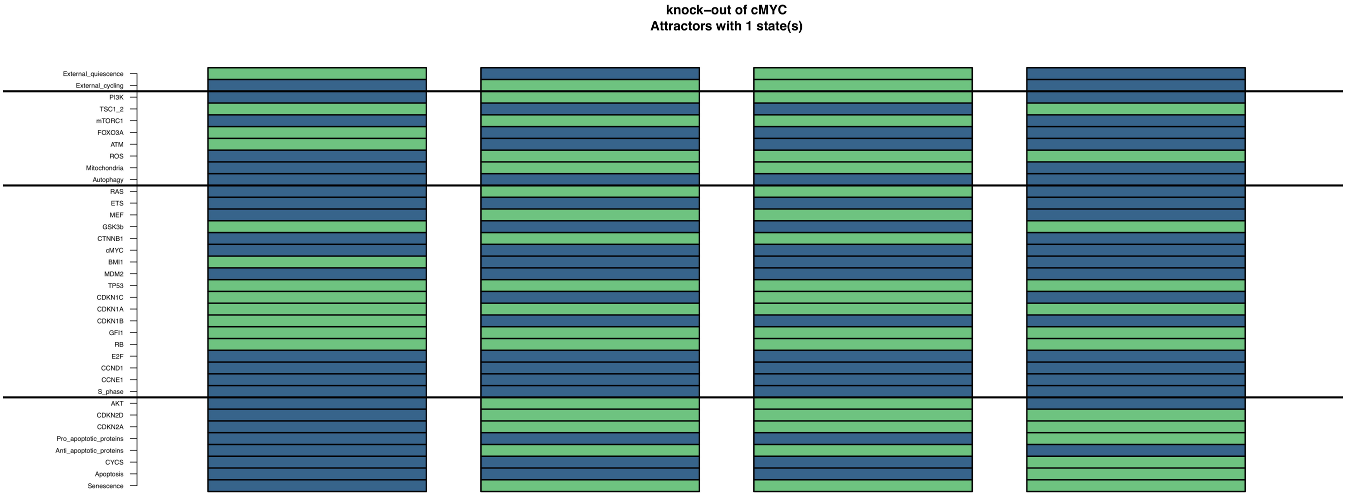
knock–out of cMYC
Attractors with 1 state(s)
External_quiescence
External_cycling
PI3K
TSC1_2
mTORC1
FOXO3A
ATM
ROS
Mitochondria
Autophagy
RAS
ETS
MEF
GSK3b
CTNNB1
cMYC
BMI1
MDM2
TP53
CDKN1C
CDKN1A
CDKN1B
GFI1
RB
E2F
CCND1
CCNE1
S_phase
AKT
CDKN2D
CDKN2A
Pro_apoptotic_proteins
Anti_apoptotic_proteins
CYCS
Apoptosis
Senescence

overexpression of cMYC
Attractors with 1 state(s)

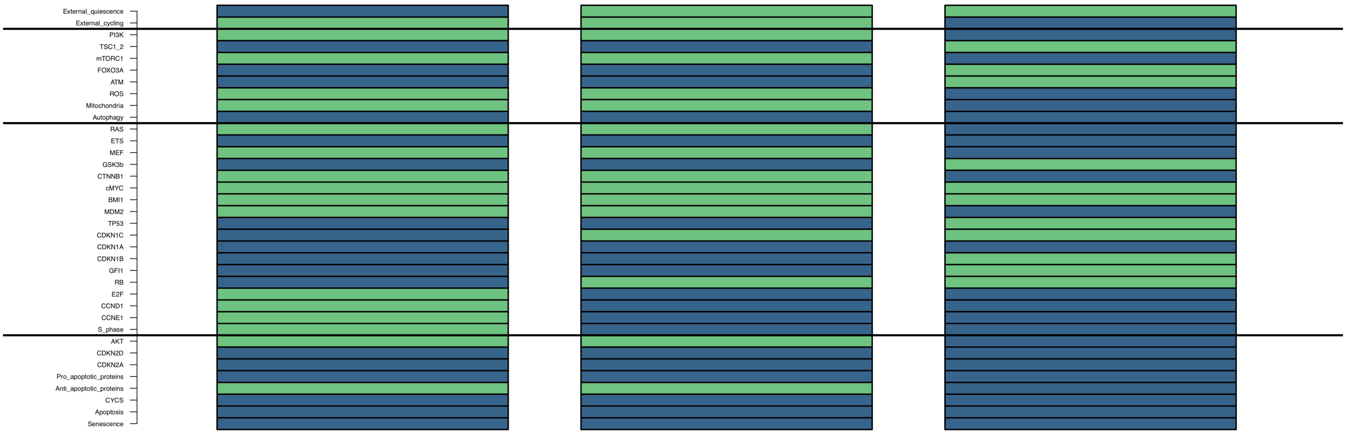

overexpression of cMYC
Attractors with 4 state(s)

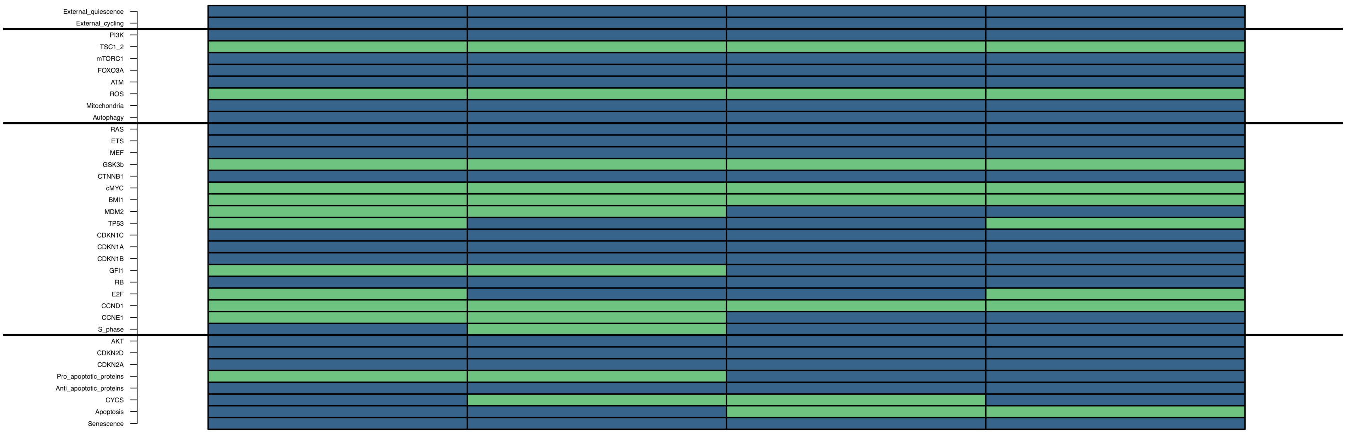

overexpression of cMYC
Attractors with 4 state(s)

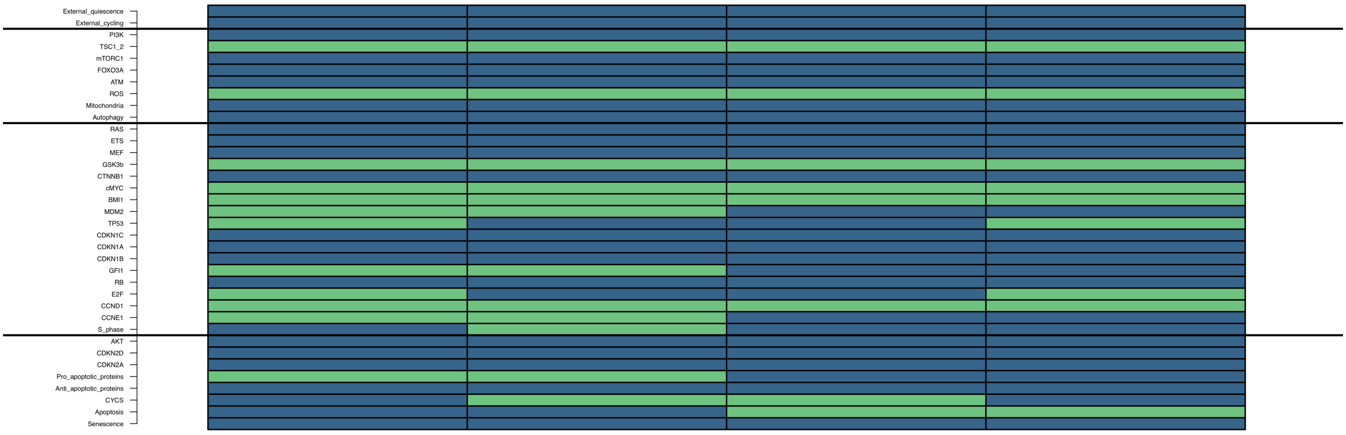

knock–out of BMI1
Attractors with 1 state(s)

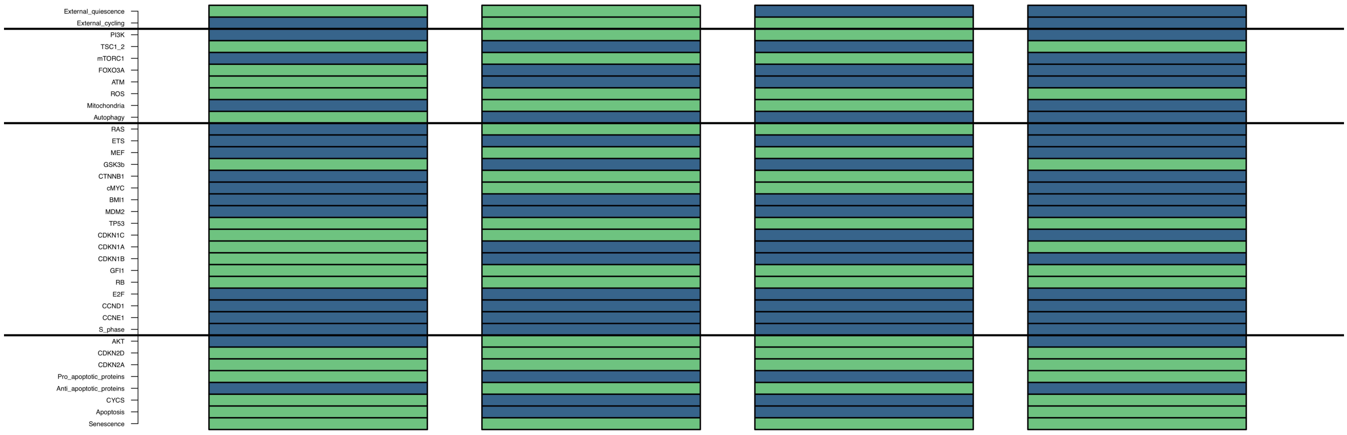

overexpression of BMI1
Attractors with 1 state(s)

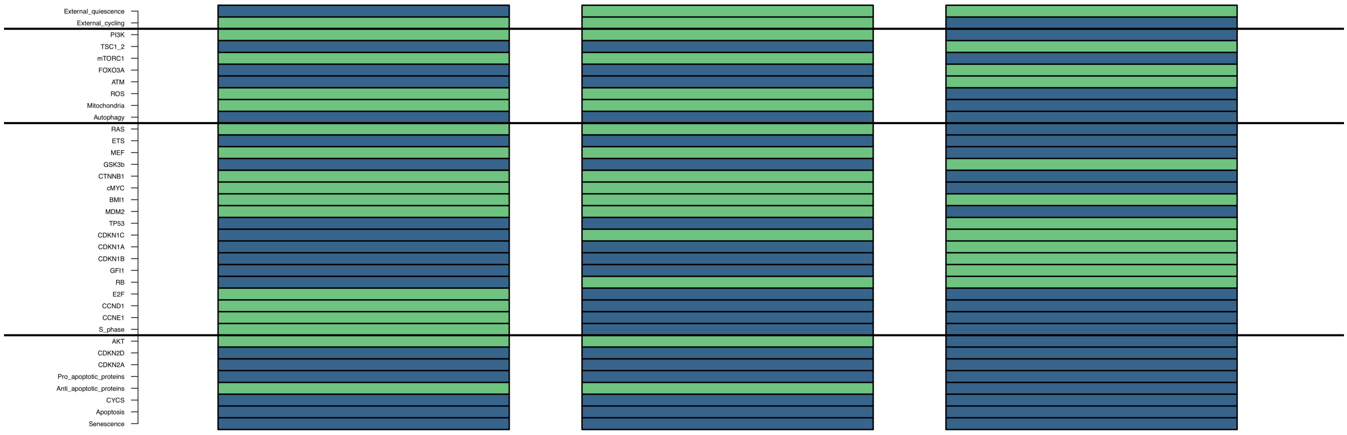

overexpression of BMI1
Attractors with 4 state(s)

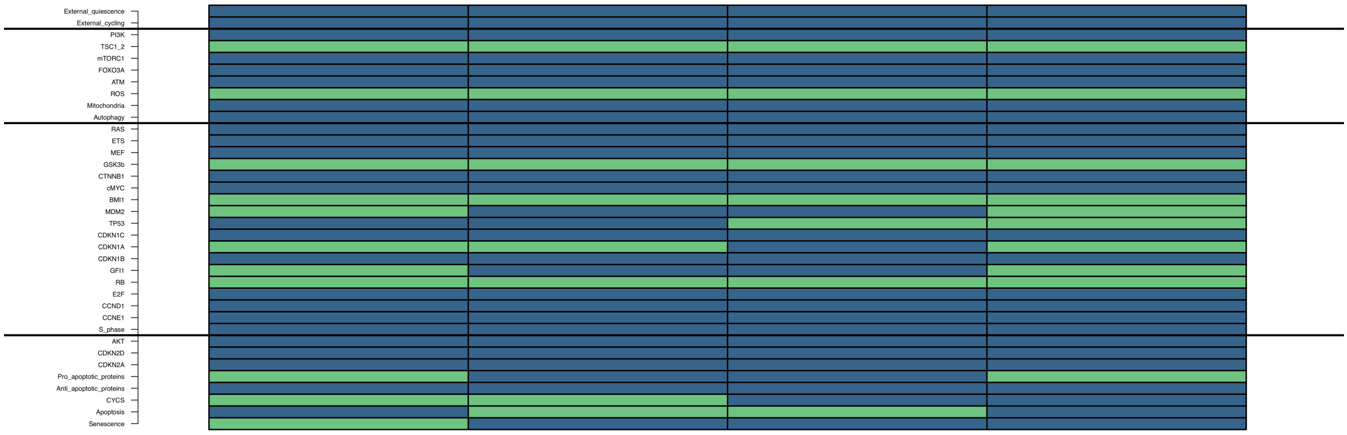

knock–out of MDM2
Attractors with 1 state(s)

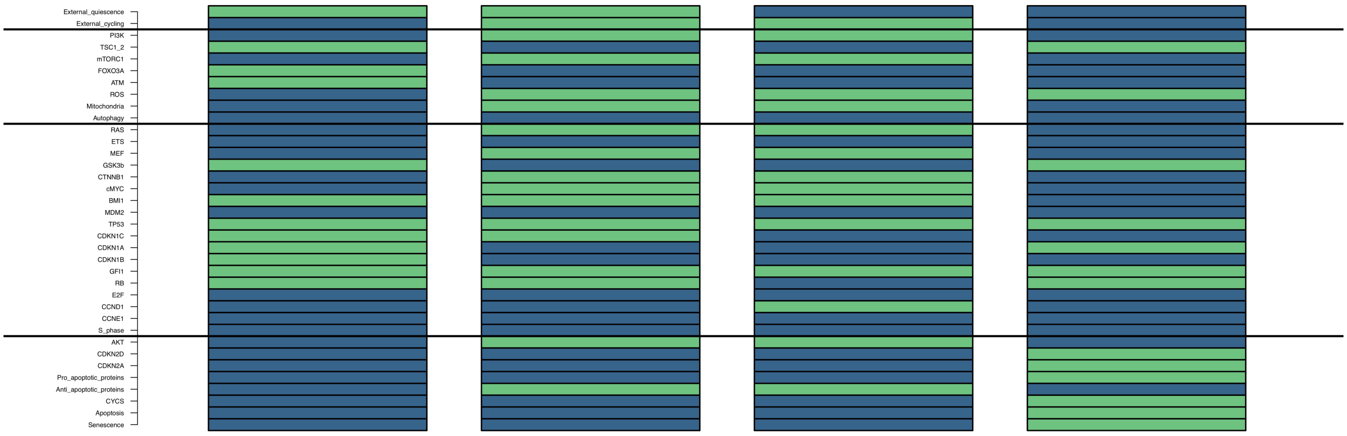

overexpression of MDM2
Attractors with 1 state(s)

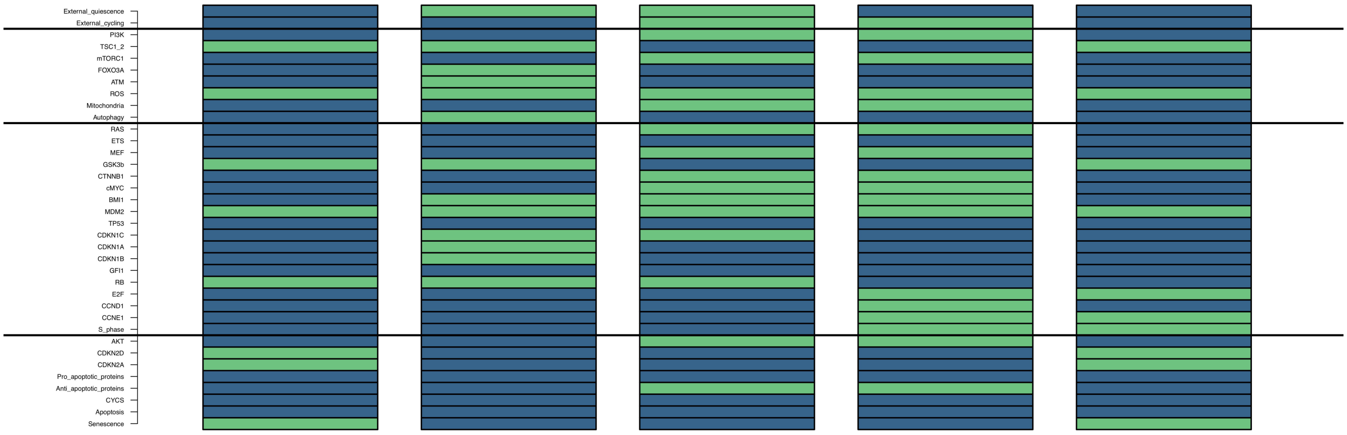

overexpression of MDM2
Attractors with 3 state(s)

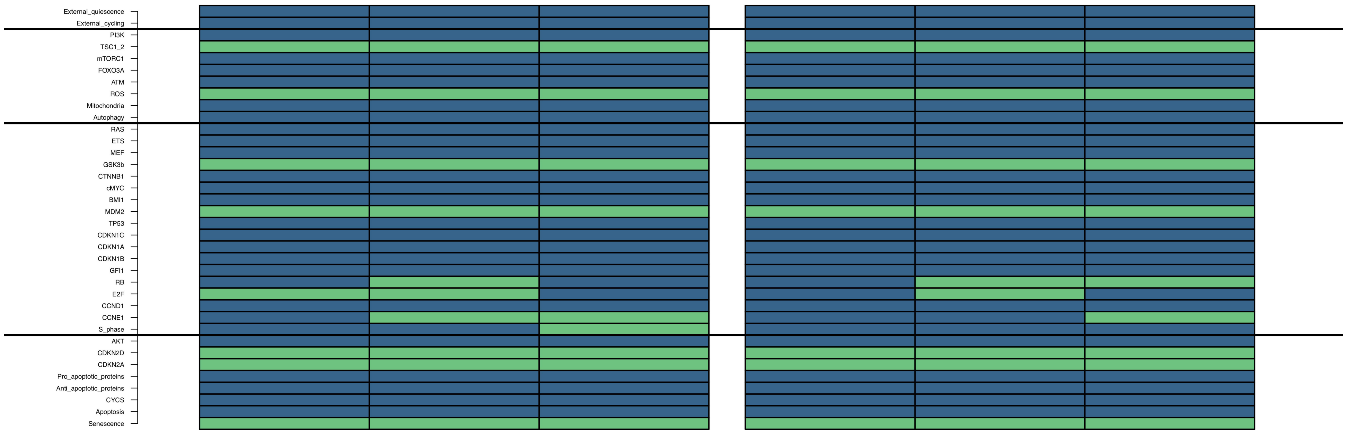

knock–out of TP53
Attractors with 1 state(s)

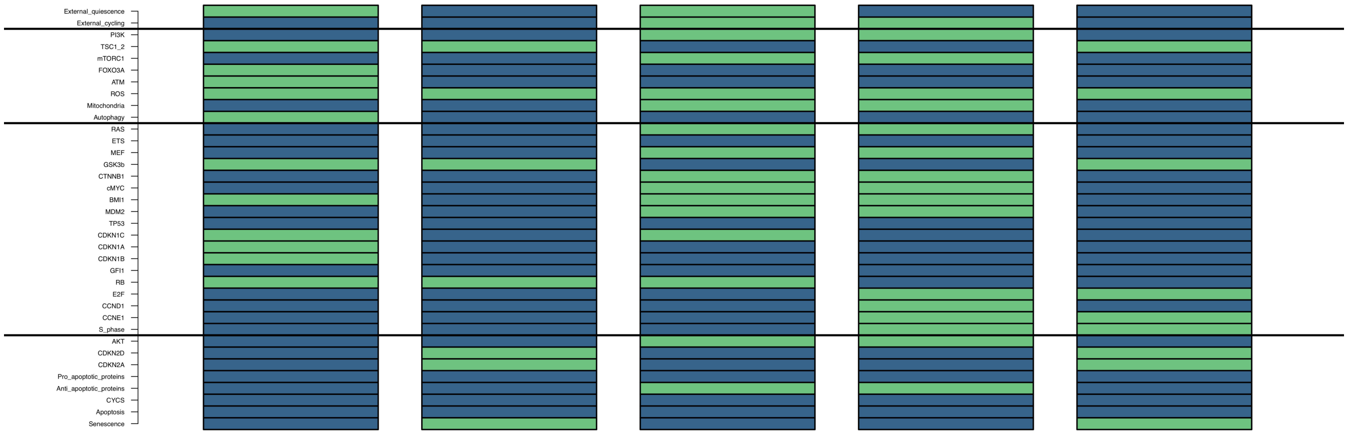

knock–out of TP53
Attractors with 3 state(s)

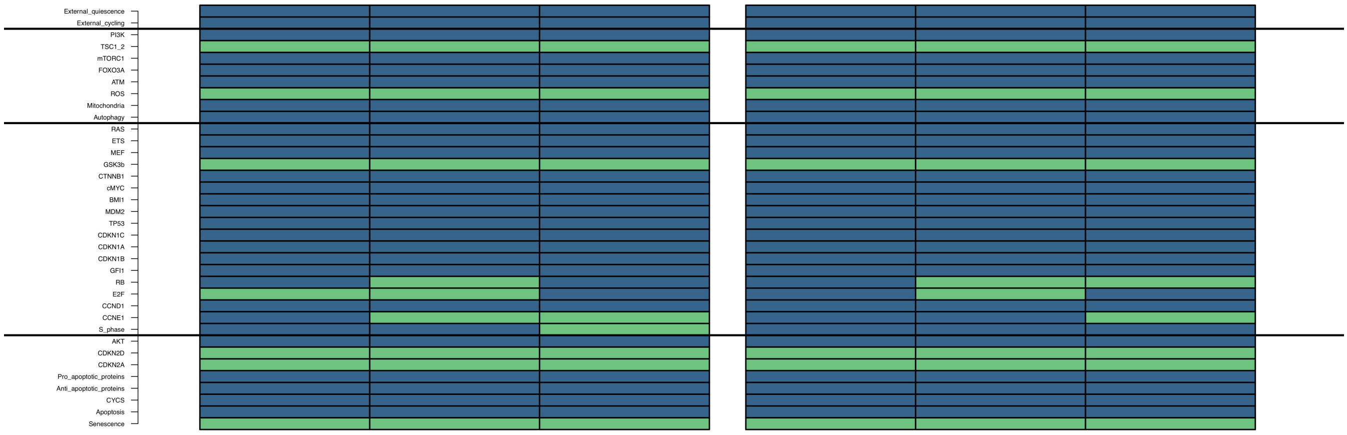

overexpression of TP53
Attractors with 1 state(s)

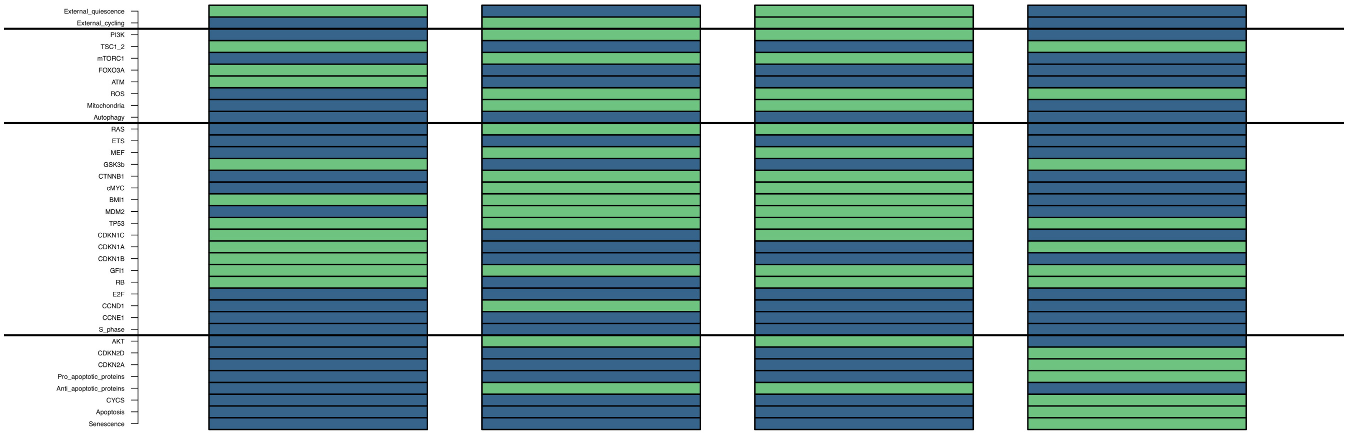

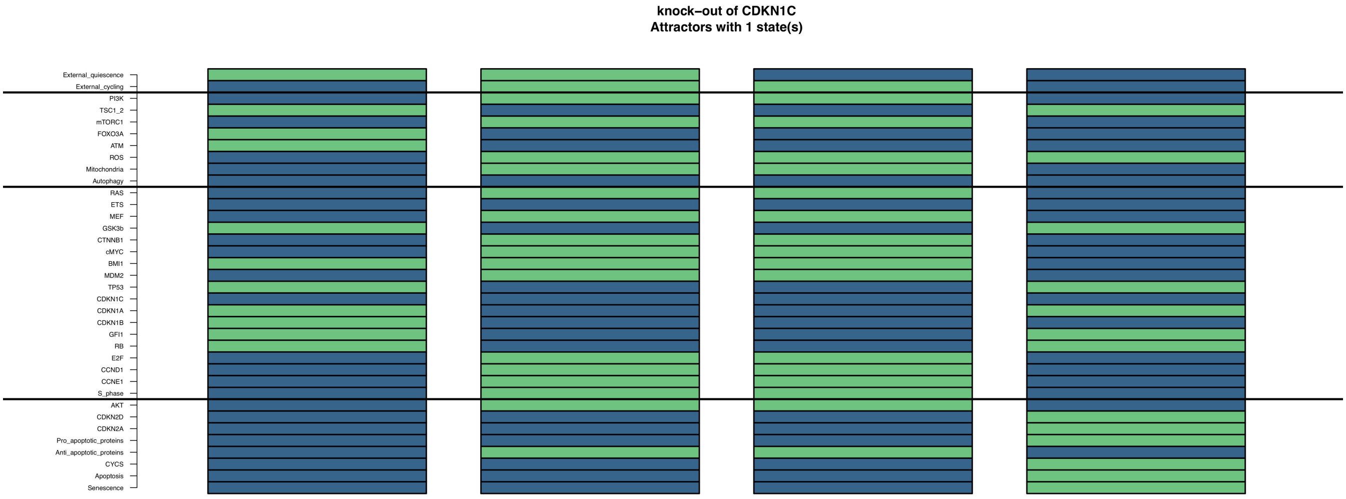
knock–out of CDKN1C
Attractors with 1 state(s)
External_quiescence
External_cycling
PI3K
TSC1_2
mTORC1
FOXO3A
ATM
ROS
Mitochondria
Autophagy
RAS
ETS
MEF
GSK3b
CTNNB1
cMYC
BMI1
MDM2
TP53
CDKN1C
CDKN1A
CDKN1B
GFI1
RB
E2F
CCND1
CCNE1
S_phase
AKT
CDKN2D
CDKN2A
Pro_apoptotic_proteins
Anti_apoptotic_proteins
CYCS
Apoptosis
Senescence

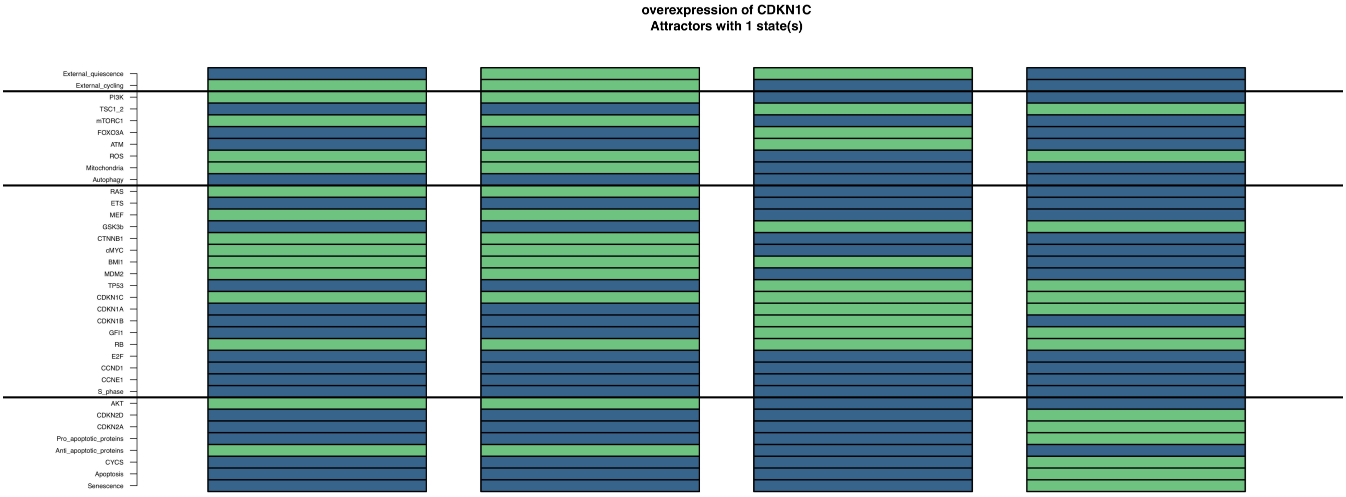
overexpression of CDKN1C
Attractors with 1 state(s)
External_quiescence
External_cycling
PI3K
TSC1_2
mTORC1
FOXO3A
ATM
ROS
Mitochondria
Autophagy
RAS
ETS
MEF
GSK3b
CTNNB1
cMYC
BMI1
MDM2
TP53
CDKN1C
CDKN1A
CDKN1B
GFI1
RB
E2F
CCND1
CCNE1
S_phase
AKT
CDKN2D
CDKN2A
Pro_apoptotic_proteins
Anti_apoptotic_proteins
CYCS
Apoptosis
Senescence

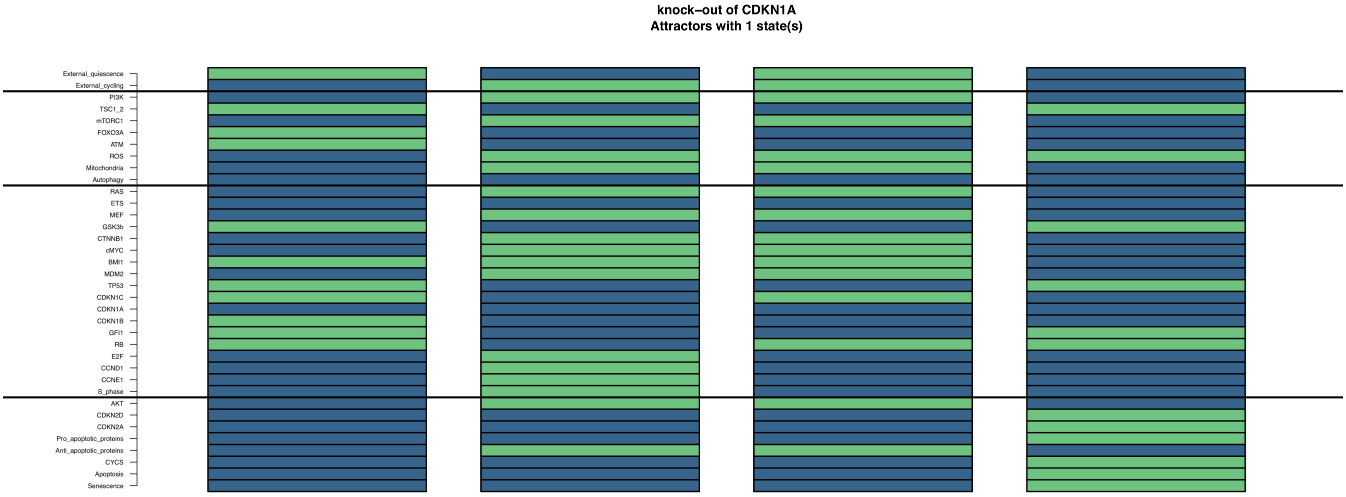
knock–out of CDKN1A
Attractors with 1 state(s)
External_quiescence
External_cycling
PI3K
TSC1_2
mTORC1
FOXO3A
ATM
ROS
Mitochondria
Autophagy
RAS
ETS
MEF
GSK3b
CTNNB1
cMYC
BMI1
MDM2
TP53
CDKN1C
CDKN1A
CDKN1B
GFI1
RB
E2F
CCND1
CCNE1
S_phase
AKT
CDKN2D
CDKN2A
Pro_apoptotic_proteins
Anti_apoptotic_proteins
CYCS
Apoptosis
Senescence

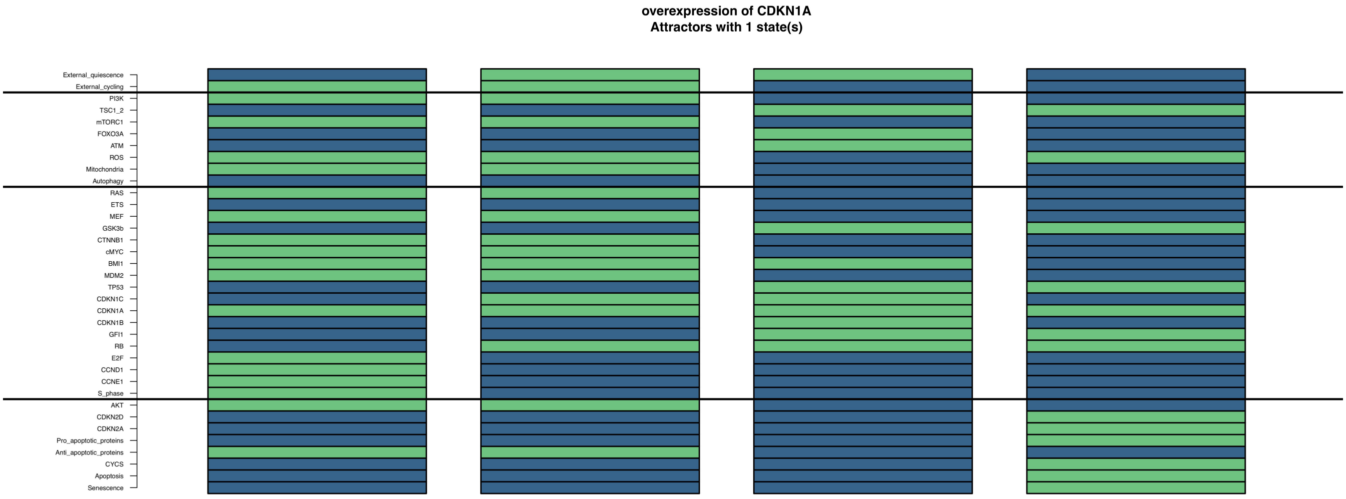
overexpression of CDKN1A
Attractors with 1 state(s)
External_quiescence
External_cycling
PI3K
TSC1_2
mTORC1
FOXO3A
ATM
ROS
Mitochondria
Autophagy
RAS
ETS
MEF
GSK3b
CTNNB1
cMYC
BMI1
MDM2
TP53
CDKN1C
CDKN1A
CDKN1B
GFI1
RB
E2F
CCND1
CCNE1
S_phase
AKT
CDKN2D
CDKN2A
Pro_apoptotic_proteins
Anti_apoptotic_proteins
CYCS
Apoptosis
Senescence

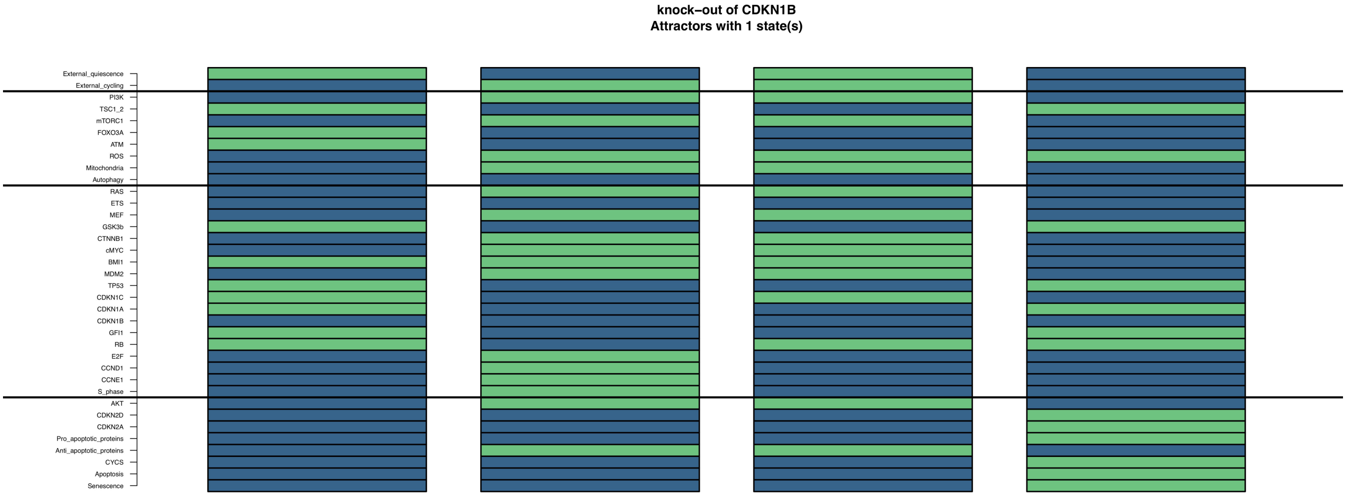
knock–out of CDKN1B
Attractors with 1 state(s)
External_quiescence
External_cycling
PI3K
TSC1_2
mTORC1
FOXO3A
ATM
ROS
Mitochondria
Autophagy
RAS
ETS
MEF
GSK3b
CTNNB1
cMYC
BMI1
MDM2
TP53
CDKN1C
CDKN1A
CDKN1B
GFI1
RB
E2F
CCND1
CCNE1
S_phase
AKT
CDKN2D
CDKN2A
Pro_apoptotic_proteins
Anti_apoptotic_proteins
CYCS
Apoptosis
Senescence

overexpression of CDKN1B
Attractors with 1 state(s)

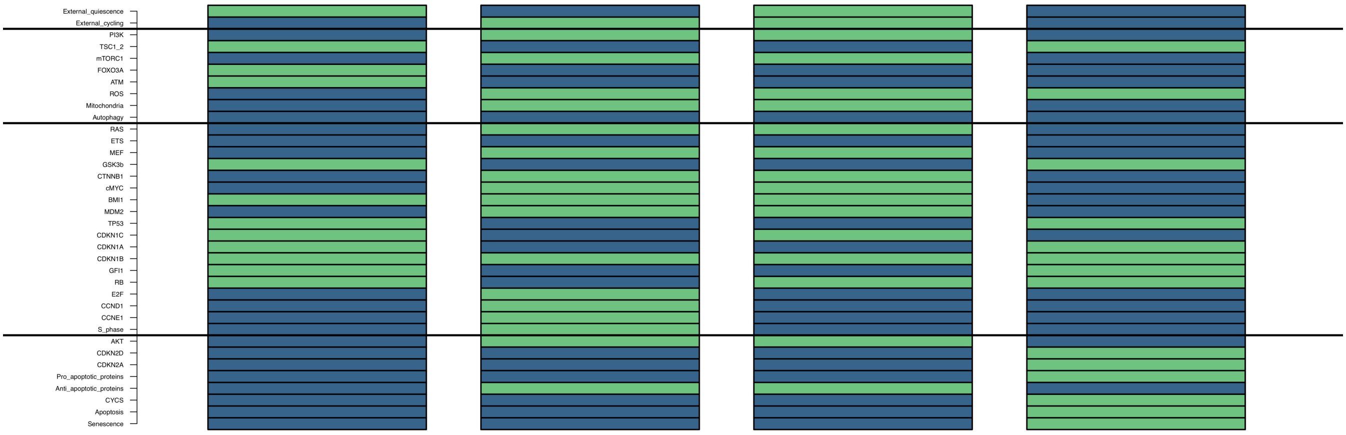

knock–out of GFI1
Attractors with 1 state(s)

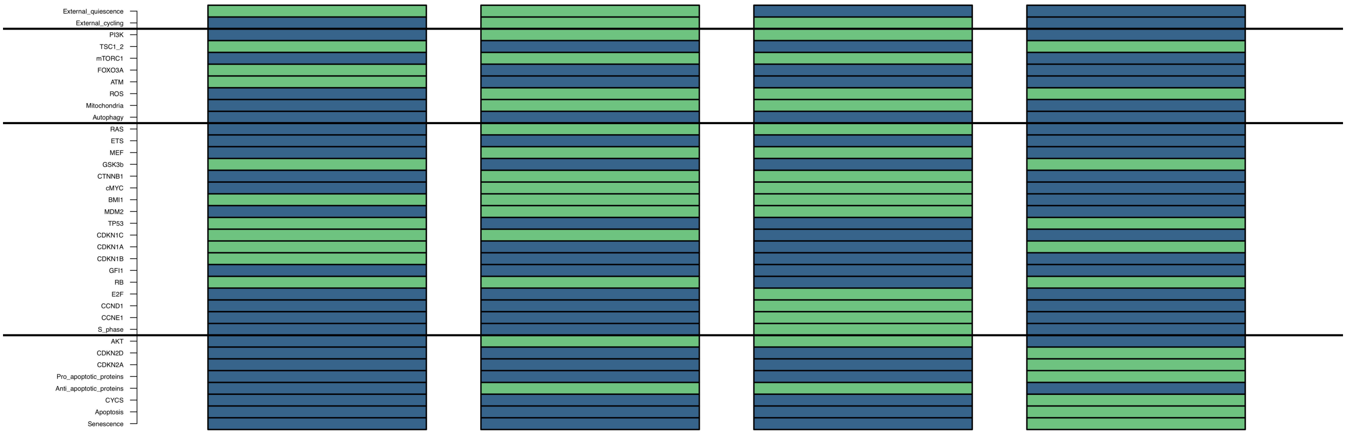

overexpression of GFI1
Attractors with 1 state(s)

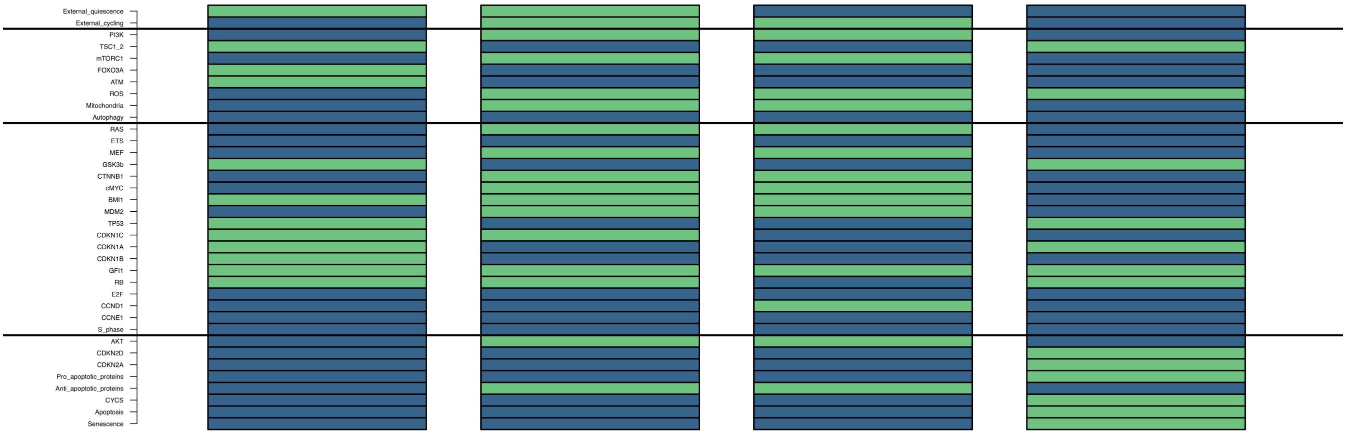

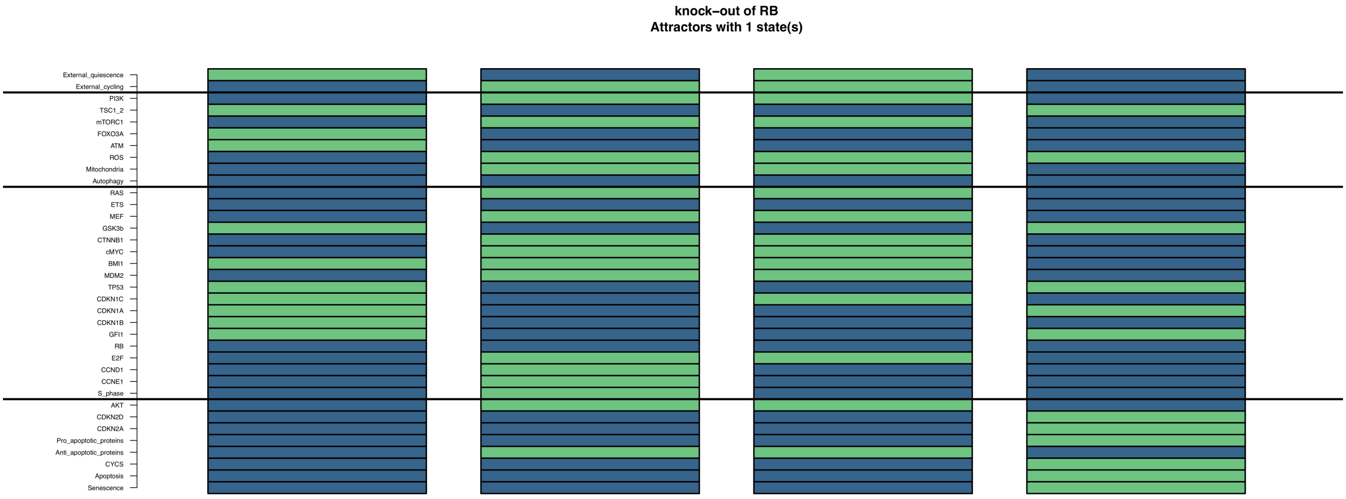
knock–out of RB
Attractors with 1 state(s)
External_quiescence
External_cycling
PI3K
TSC1_2
mTORC1
FOXO3A
ATM
ROS
Mitochondria
Autophagy
RAS
ETS
MEF
GSK3b
CTNNB1
cMYC
BMI1
MDM2
TP53
CDKN1C
CDKN1A
CDKN1B
GFI1
RB
E2F
CCND1
CCNE1
S_phase
AKT
CDKN2D
CDKN2A
Pro_apoptotic_proteins
Anti_apoptotic_proteins
CYCS
Apoptosis
Senescence

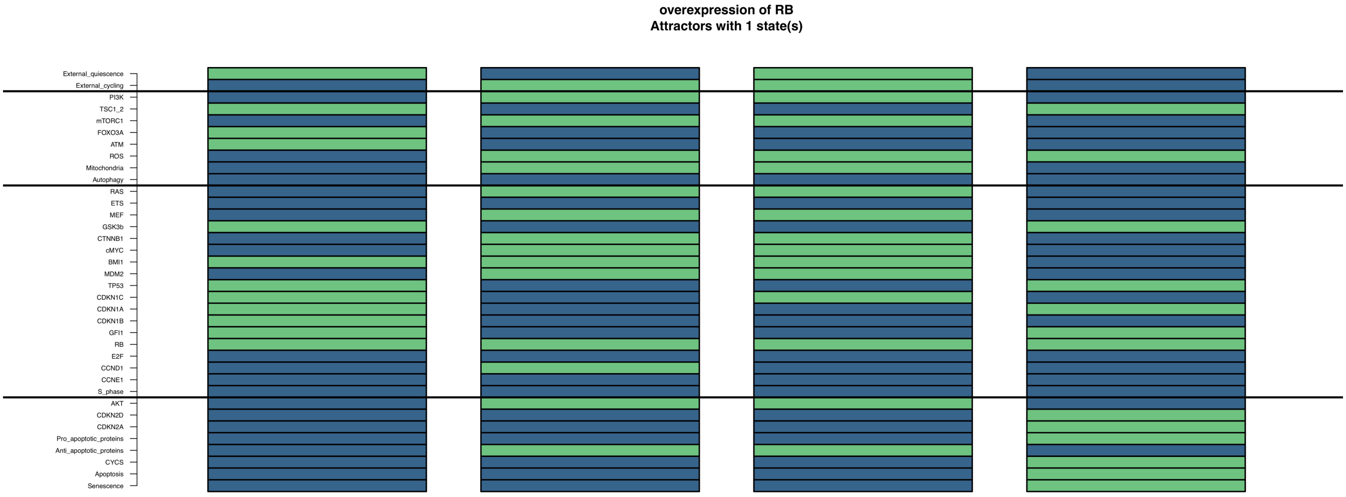
overexpression of RB
Attractors with 1 state(s)
External_quiescence
External_cycling
PI3K
TSC1_2
mTORC1
FOXO3A
ATM
ROS
Mitochondria
Autophagy
RAS
ETS
MEF
GSK3b
CTNNB1
cMYC
BMI1
MDM2
TP53
CDKN1C
CDKN1A
CDKN1B
GFI1
RB
E2F
CCND1
CCNE1
S_phase
AKT
CDKN2D
CDKN2A
Pro_apoptotic_proteins
Anti_apoptotic_proteins
CYCS
Apoptosis
Senescence

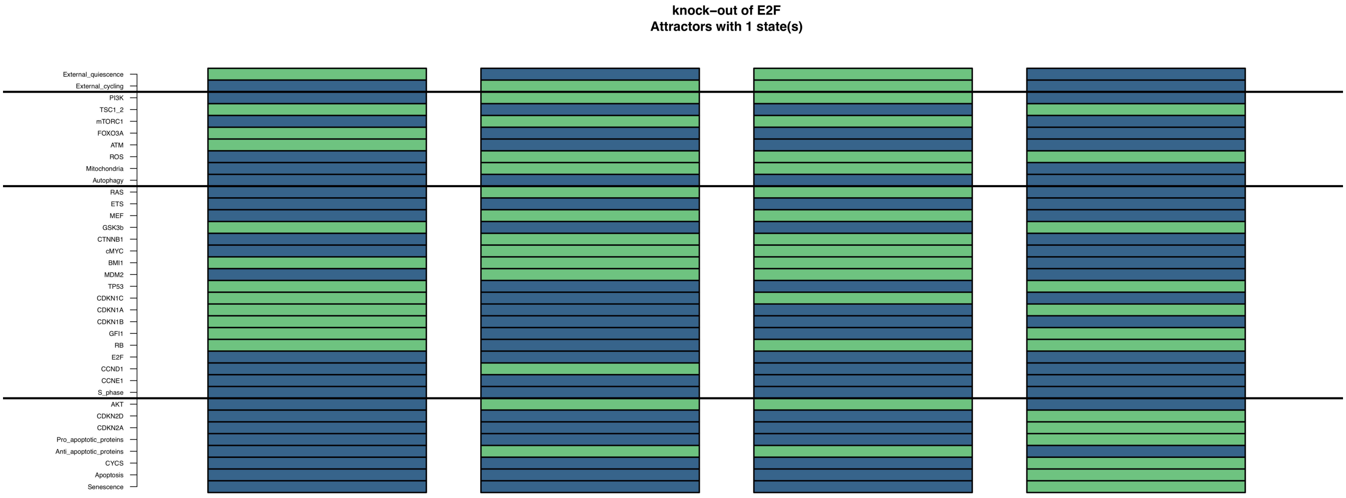
knock–out of E2F
Attractors with 1 state(s)
External_quiescence
External_cycling
PI3K
TSC1_2
mTORC1
FOXO3A
ATM
ROS
Mitochondria
Autophagy
RAS
ETS
MEF
GSK3b
CTNNB1
cMYC
BMI1
MDM2
TP53
CDKN1C
CDKN1A
CDKN1B
GFI1
RB
E2F
CCND1
CCNE1
S_phase
AKT
CDKN2D
CDKN2A
Pro_apoptotic_proteins
Anti_apoptotic_proteins
CYCS
Apoptosis
Senescence

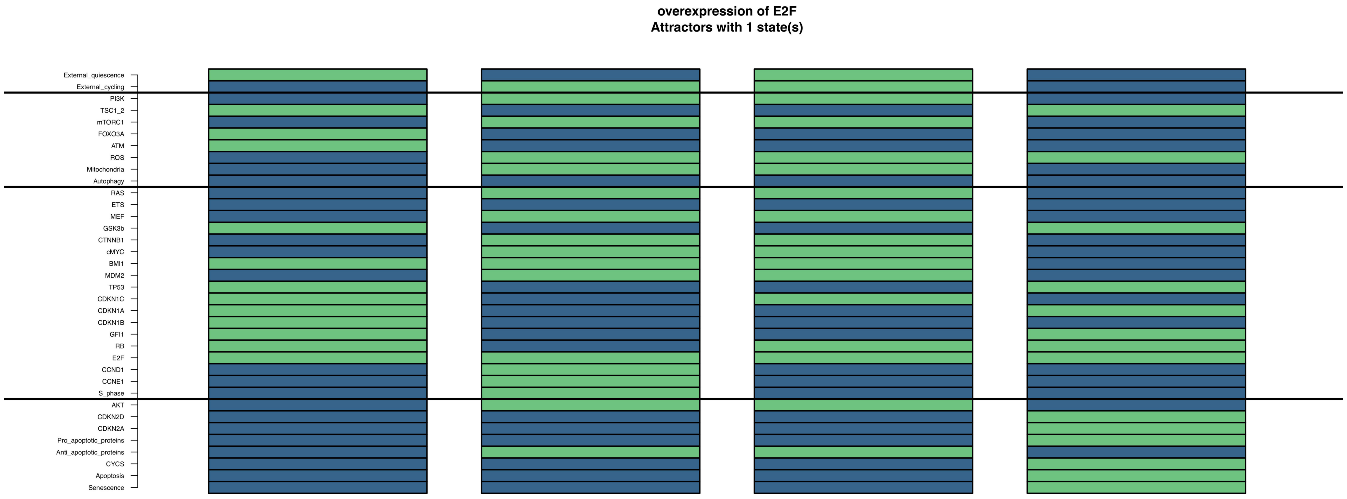
overexpression of E2F
Attractors with 1 state(s)
External_quiescence
External_cycling
PI3K
TSC1_2
mTORC1
FOXO3A
ATM
ROS
Mitochondria
Autophagy
RAS
ETS
MEF
GSK3b
CTNNB1
cMYC
BMI1
MDM2
TP53
CDKN1C
CDKN1A
CDKN1B
GFI1
RB
E2F
CCND1
CCNE1
S_phase
AKT
CDKN2D
CDKN2A
Pro_apoptotic_proteins
Anti_apoptotic_proteins
CYCS
Apoptosis
Senescence

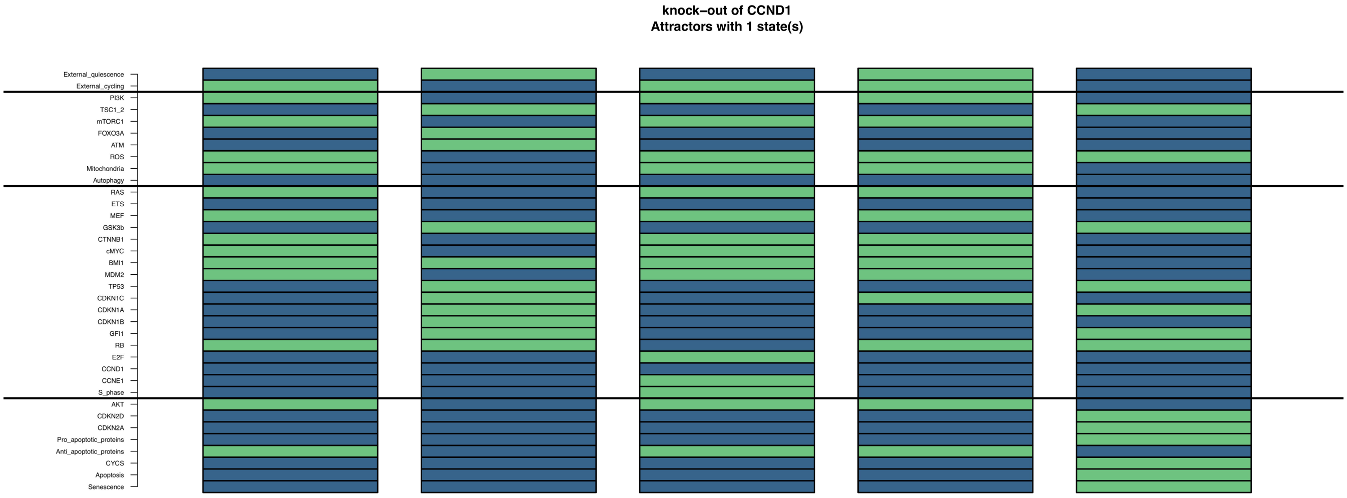
knock–out of CCND1
Attractors with 1 state(s)
External_quiescence
External_cycling
PI3K
TSC1_2
mTORC1
FOXO3A
ATM
ROS
Mitochondria
Autophagy
RAS
ETS
MEF
GSK3b
CTNNB1
cMYC
BMI1
MDM2
TP53
CDKN1C
CDKN1A
CDKN1B
GFI1
RB
E2F
CCND1
CCNE1
S_phase
AKT
CDKN2D
CDKN2A
Pro_apoptotic_proteins
Anti_apoptotic_proteins
CYCS
Apoptosis
Senescence

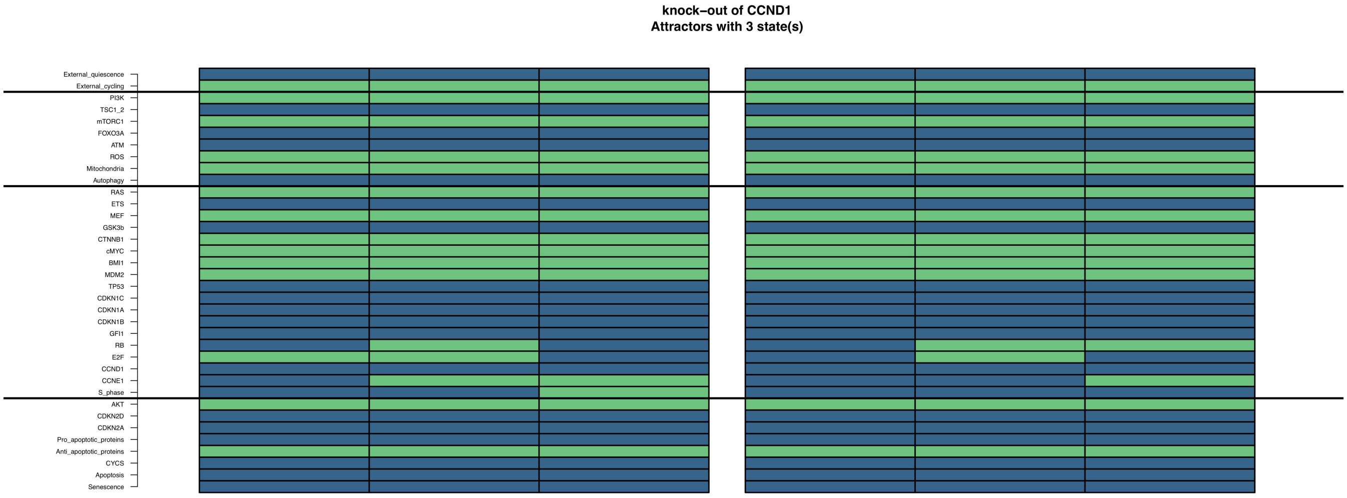
knock–out of CCND1
Attractors with 3 state(s)
External_quiescence
External_cycling
PI3K
TSC1_2
mTORC1
FOXO3A
ATM
ROS
Mitochondria
Autophagy
RAS
ETS
MEF
GSK3b
CTNNB1
cMYC
BMI1
MDM2
TP53
CDKN1C
CDKN1A
CDKN1B
GFI1
RB
E2F
CCND1
CCNE1
S_phase
AKT
CDKN2D
CDKN2A
Pro_apoptotic_proteins
Anti_apoptotic_proteins
CYCS
Apoptosis
Senescence

overexpression of CCND1
Attractors with 1 state(s)

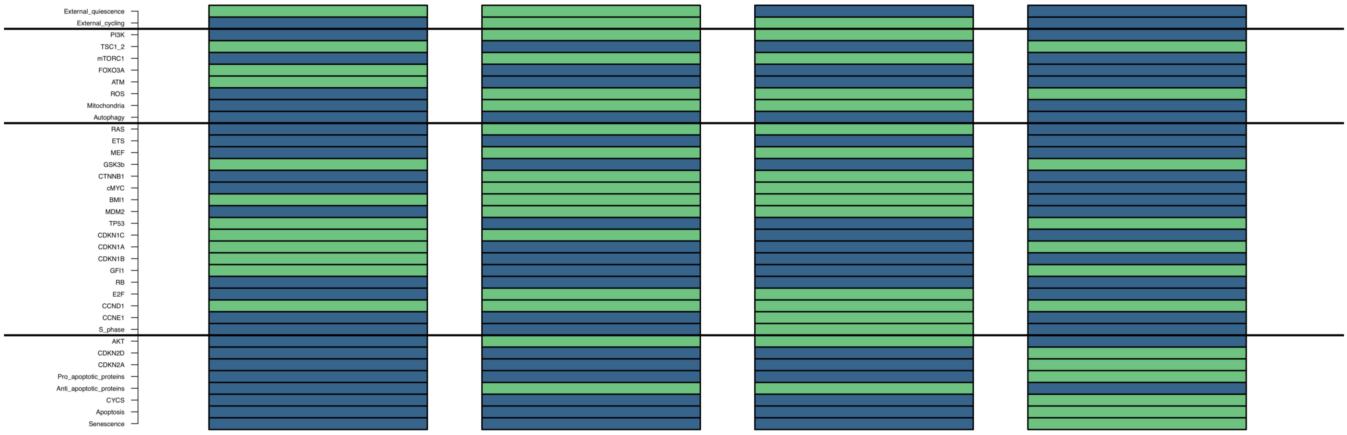

knock–out of CCNE1
Attractors with 1 state(s)

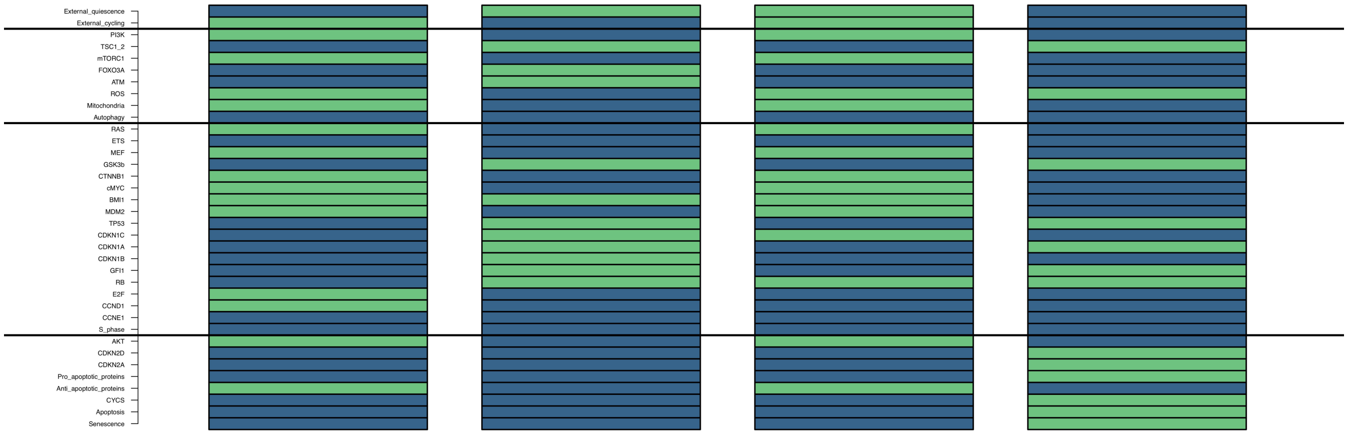

overexpression of CCNE1
Attractors with 1 state(s)

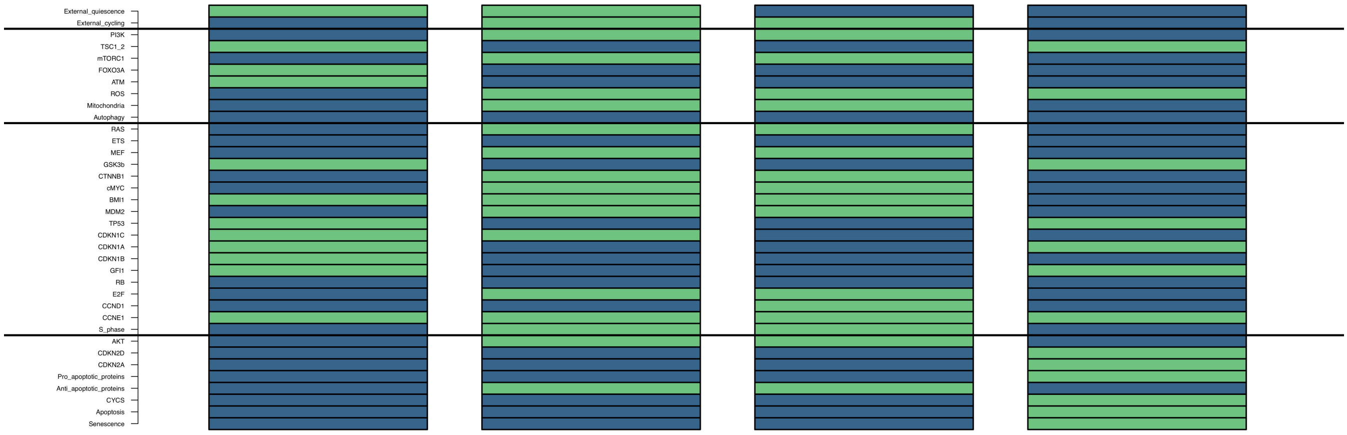

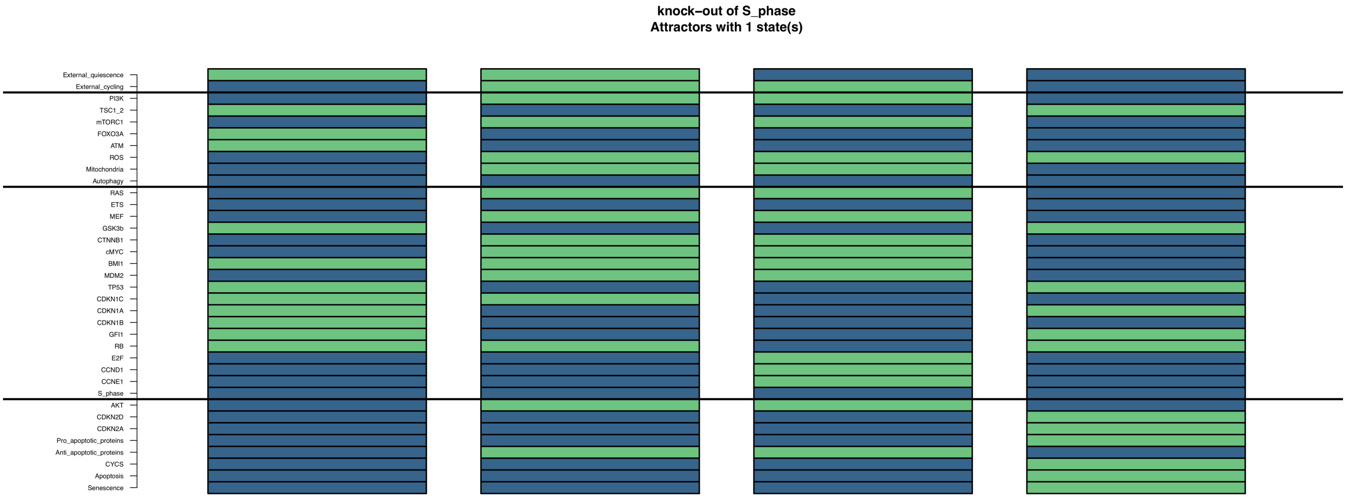
knock–out of S_phase
Attractors with 1 state(s)
External_quiescence
External_cycling
PI3K
TSC1_2
mTORC1
FOXO3A
ATM
ROS
Mitochondria
Autophagy
RAS
ETS
MEF
GSK3b
CTNNB1
cMYC
BMI1
MDM2
TP53
CDKN1C
CDKN1A
CDKN1B
GFI1
RB
E2F
CCND1
CCNE1
S_phase
AKT
CDKN2D
CDKN2A
Pro_apoptotic_proteins
Anti_apoptotic_proteins
CYCS
Apoptosis
Senescence

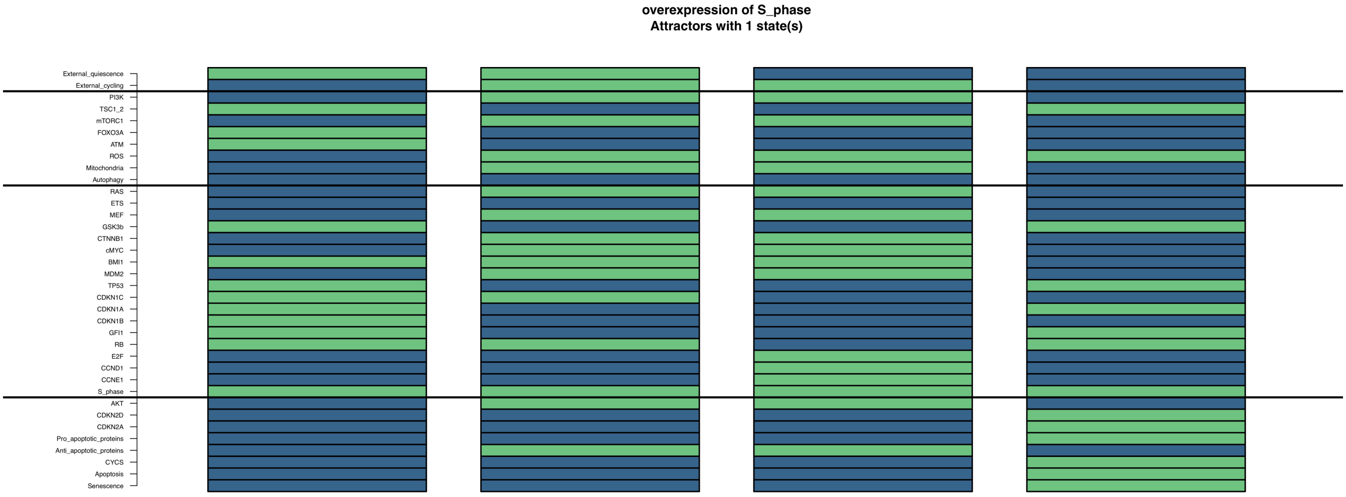
overexpression of S_phase
Attractors with 1 state(s)
External_quiescence
External_cycling
PI3K
TSC1_2
mTORC1
FOXO3A
ATM
ROS
Mitochondria
Autophagy
RAS
ETS
MEF
GSK3b
CTNNB1
cMYC
BMI1
MDM2
TP53
CDKN1C
CDKN1A
CDKN1B
GFI1
RB
E2F
CCND1
CCNE1
S_phase
AKT
CDKN2D
CDKN2A
Pro_apoptotic_proteins
Anti_apoptotic_proteins
CYCS
Apoptosis
Senescence

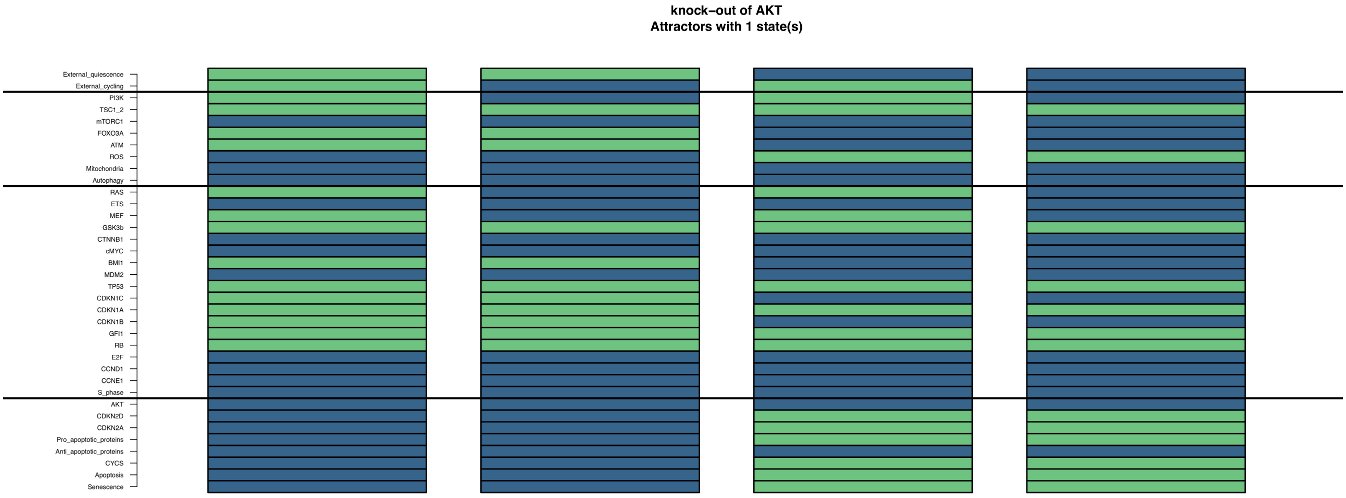
knock–out of AKT
Attractors with 1 state(s)
External_quiescence
External_cycling
PI3K
TSC1_2
mTORC1
FOXO3A
ATM
ROS
Mitochondria
Autophagy
RAS
ETS
MEF
GSK3b
CTNNB1
cMYC
BMI1
MDM2
TP53
CDKN1C
CDKN1A
CDKN1B
GFI1
RB
E2F
CCND1
CCNE1
S_phase
AKT
CDKN2D
CDKN2A
Pro_apoptotic_proteins
Anti_apoptotic_proteins
CYCS
Apoptosis
Senescence

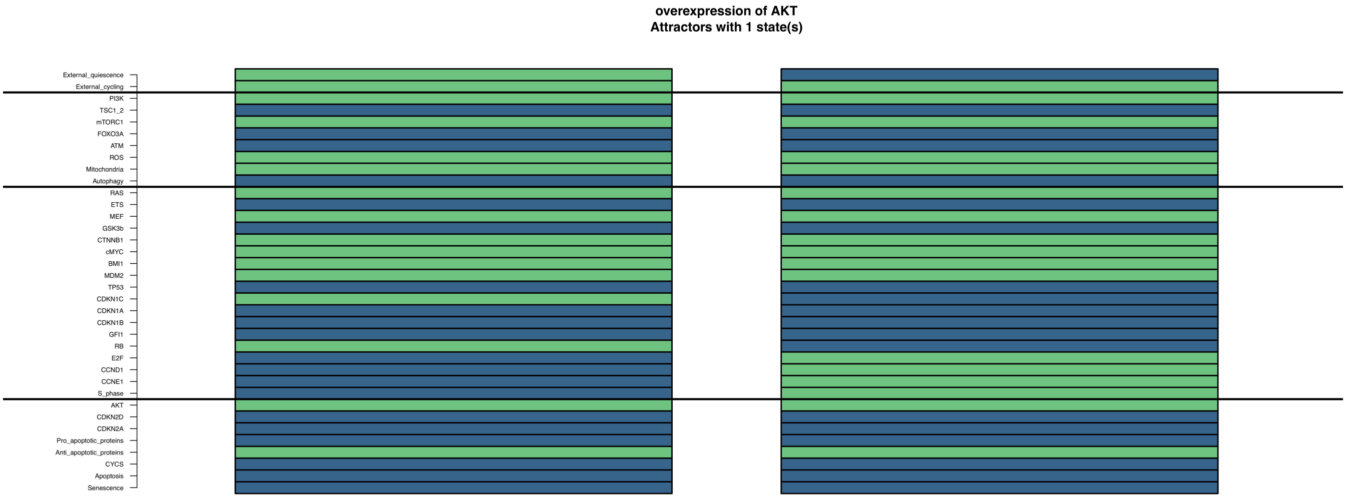
overexpression of AKT
Attractors with 1 state(s)
External_quiescence
External_cycling
PI3K
TSC1_2
mTORC1
FOXO3A
ATM
ROS
Mitochondria
Autophagy
RAS
ETS
MEF
GSK3b
CTNNB1
cMYC
BMI1
MDM2
TP53
CDKN1C
CDKN1A
CDKN1B
GFI1
RB
E2F
CCND1
CCNE1
S_phase
AKT
CDKN2D
CDKN2A
Pro_apoptotic_proteins
Anti_apoptotic_proteins
CYCS
Apoptosis
Senescence

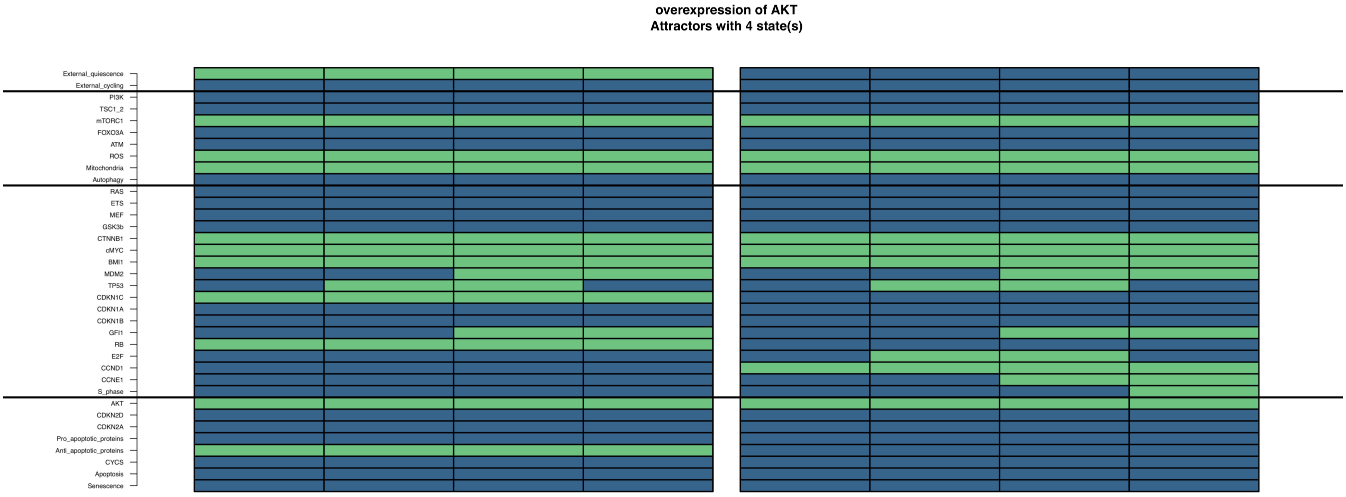
overexpression of AKT
Attractors with 4 state(s)
External_quiescence
External_cycling
PI3K
TSC1_2
mTORC1
FOXO3A
ATM
ROS
Mitochondria
Autophagy
RAS
ETS
MEF
GSK3b
CTNNB1
cMYC
BMI1
MDM2
TP53
CDKN1C
CDKN1A
CDKN1B
GFI1
RB
E2F
CCND1
CCNE1
S_phase
AKT
CDKN2D
CDKN2A
Pro_apoptotic_proteins
Anti_apoptotic_proteins
CYCS
Apoptosis
Senescence

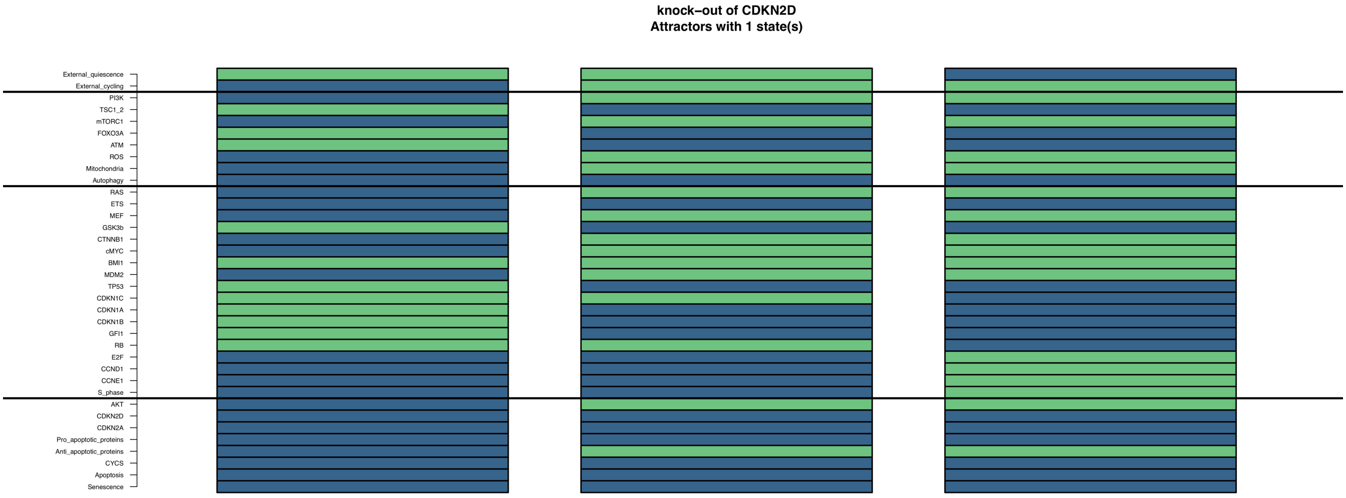
knock–out of CDKN2D
Attractors with 1 state(s)
External_quiescence
External_cycling
PI3K
TSC1_2
mTORC1
FOXO3A
ATM
ROS
Mitochondria
Autophagy
RAS
ETS
MEF
GSK3b
CTNNB1
cMYC
BMI1
MDM2
TP53
CDKN1C
CDKN1A
CDKN1B
GFI1
RB
E2F
CCND1
CCNE1
S_phase
AKT
CDKN2D
CDKN2A
Pro_apoptotic_proteins
Anti_apoptotic_proteins
CYCS
Apoptosis
Senescence

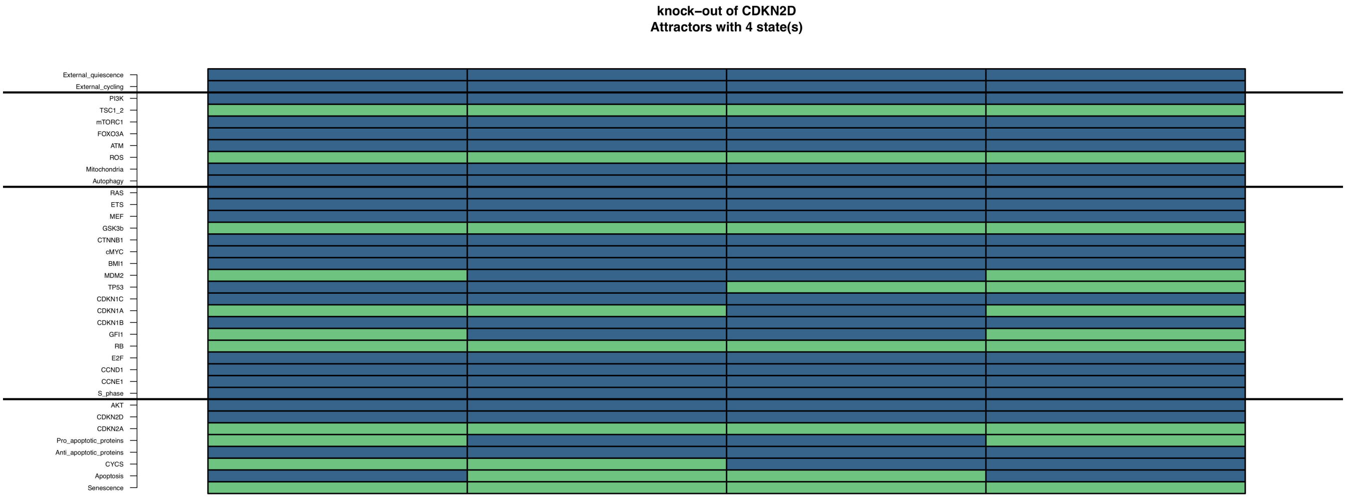
knock–out of CDKN2D
Attractors with 4 state(s)
External_quiescence
External_cycling
PI3K
TSC1_2
mTORC1
FOXO3A
ATM
ROS
Mitochondria
Autophagy
RAS
ETS
MEF
GSK3b
CTNNB1
cMYC
BMI1
MDM2
TP53
CDKN1C
CDKN1A
CDKN1B
GFI1
RB
E2F
CCND1
CCNE1
S_phase
AKT
CDKN2D
CDKN2A
Pro_apoptotic_proteins
Anti_apoptotic_proteins
CYCS
Apoptosis
Senescence

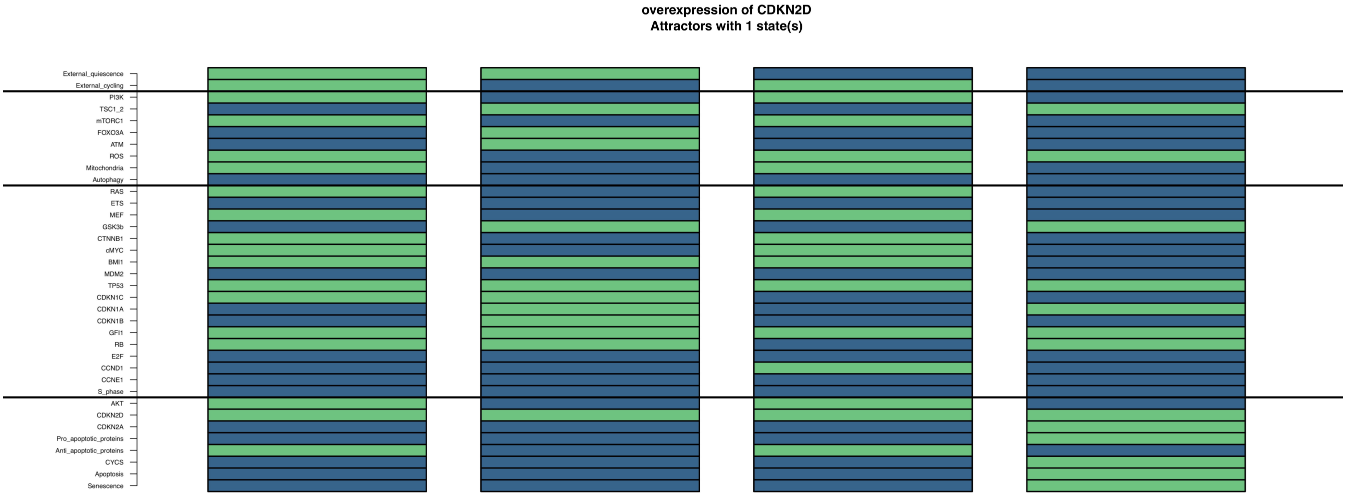
overexpression of CDKN2D
Attractors with 1 state(s)
External_quiescence
External_cycling
PI3K
TSC1_2
mTORC1
FOXO3A
ATM
ROS
Mitochondria
Autophagy
RAS
ETS
MEF
GSK3b
CTNNB1
cMYC
BMI1
MDM2
TP53
CDKN1C
CDKN1A
CDKN1B
GFI1
RB
E2F
CCND1
CCNE1
S_phase
AKT
CDKN2D
CDKN2A
Pro_apoptotic_proteins
Anti_apoptotic_proteins
CYCS
Apoptosis
Senescence

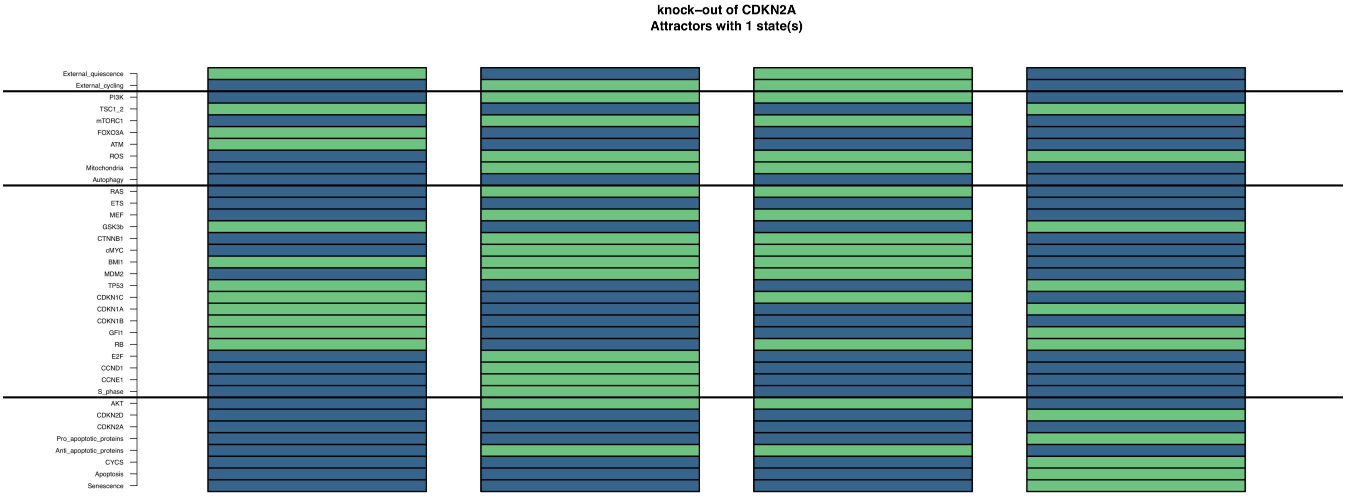
knock–out of CDKN2A
Attractors with 1 state(s)
External_quiescence
External_cycling
PI3K
TSC1_2
mTORC1
FOXO3A
ATM
ROS
Mitochondria
Autophagy
RAS
ETS
MEF
GSK3b
CTNNB1
cMYC
BMI1
MDM2
TP53
CDKN1C
CDKN1A
CDKN1B
GFI1
RB
E2F
CCND1
CCNE1
S_phase
AKT
CDKN2D
CDKN2A
Pro_apoptotic_proteins
Anti_apoptotic_proteins
CYCS
Apoptosis
Senescence

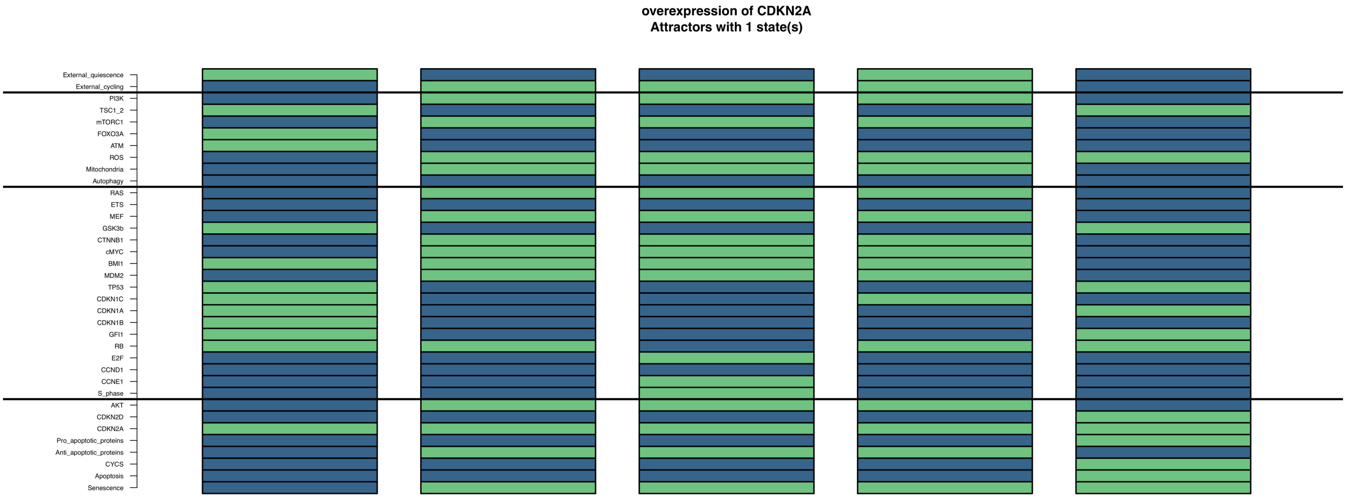
overexpression of CDKN2A
Attractors with 1 state(s)
External_quiescence
External_cycling
PI3K
TSC1_2
mTORC1
FOXO3A
ATM
ROS
Mitochondria
Autophagy
RAS
ETS
MEF
GSK3b
CTNNB1
cMYC
BMI1
MDM2
TP53
CDKN1C
CDKN1A
CDKN1B
GFI1
RB
E2F
CCND1
CCNE1
S_phase
AKT
CDKN2D
CDKN2A
Pro_apoptotic_proteins
Anti_apoptotic_proteins
CYCS
Apoptosis
Senescence

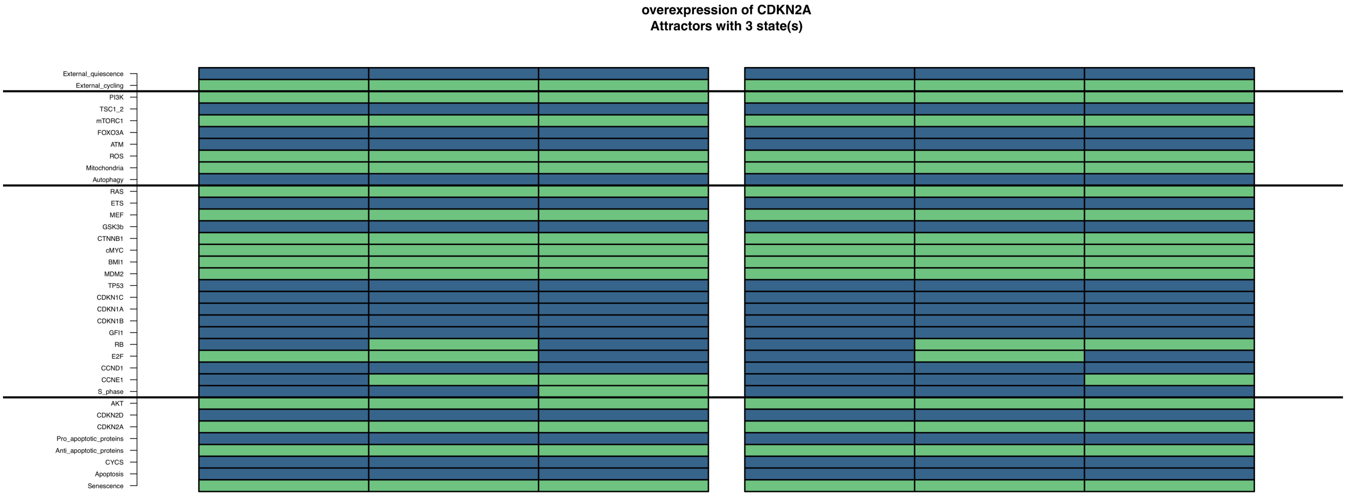
overexpression of CDKN2A
Attractors with 3 state(s)
External_quiescence
External_cycling
PI3K
TSC1_2
mTORC1
FOXO3A
ATM
ROS
Mitochondria
Autophagy
RAS
ETS
MEF
GSK3b
CTNNB1
cMYC
BMI1
MDM2
TP53
CDKN1C
CDKN1A
CDKN1B
GFI1
RB
E2F
CCND1
CCNE1
S_phase
AKT
CDKN2D
CDKN2A
Pro_apoptotic_proteins
Anti_apoptotic_proteins
CYCS
Apoptosis
Senescence

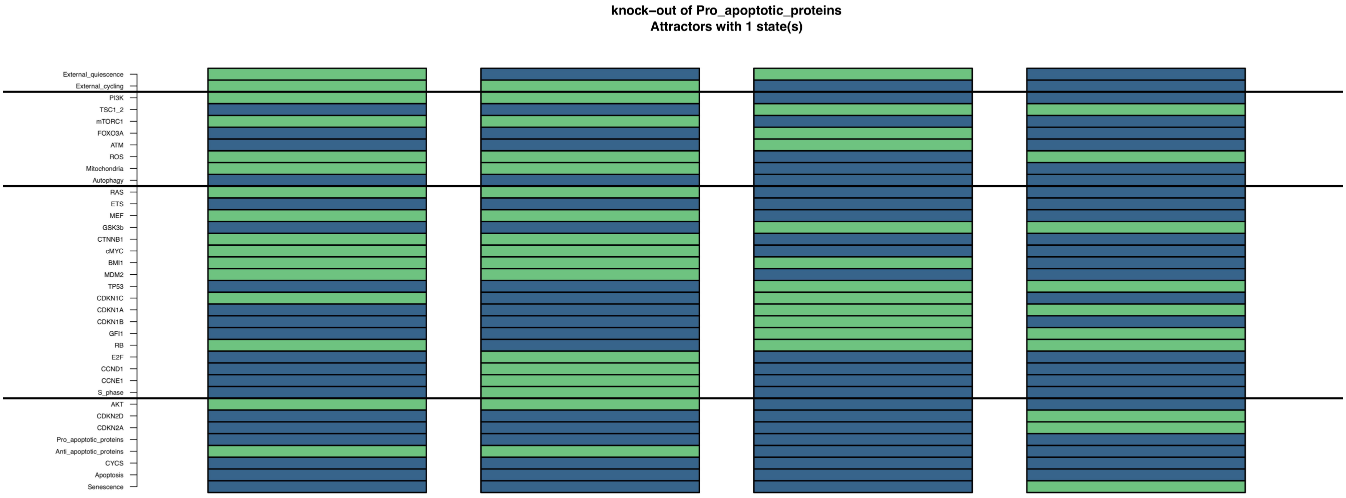
knock–out of Pro_apoptotic_proteins
Attractors with 1 state(s)
External_quiescence
External_cycling
PI3K
TSC1_2
mTORC1
FOXO3A
ATM
ROS
Mitochondria
Autophagy
RAS
ETS
MEF
GSK3b
CTNNB1
cMYC
BMI1
MDM2
TP53
CDKN1C
CDKN1A
CDKN1B
GFI1
RB
E2F
CCND1
CCNE1
S_phase
AKT
CDKN2D
CDKN2A
Pro_apoptotic_proteins
Anti_apoptotic_proteins
CYCS
Apoptosis
Senescence

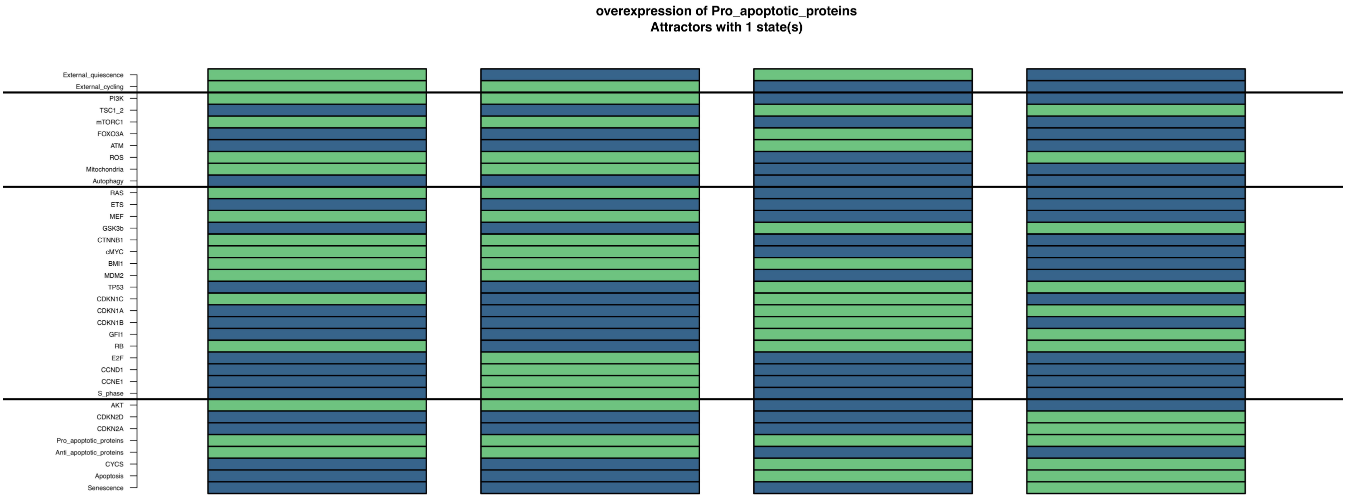
overexpression of Pro_apoptotic_proteins
Attractors with 1 state(s)
External_quiescence
External_cycling
PI3K
TSC1_2
mTORC1
FOXO3A
ATM
ROS
Mitochondria
Autophagy
RAS
ETS
MEF
GSK3b
CTNNB1
cMYC
BMI1
MDM2
TP53
CDKN1C
CDKN1A
CDKN1B
GFI1
RB
E2F
CCND1
CCNE1
S_phase
AKT
CDKN2D
CDKN2A
Pro_apoptotic_proteins
Anti_apoptotic_proteins
CYCS
Apoptosis
Senescence

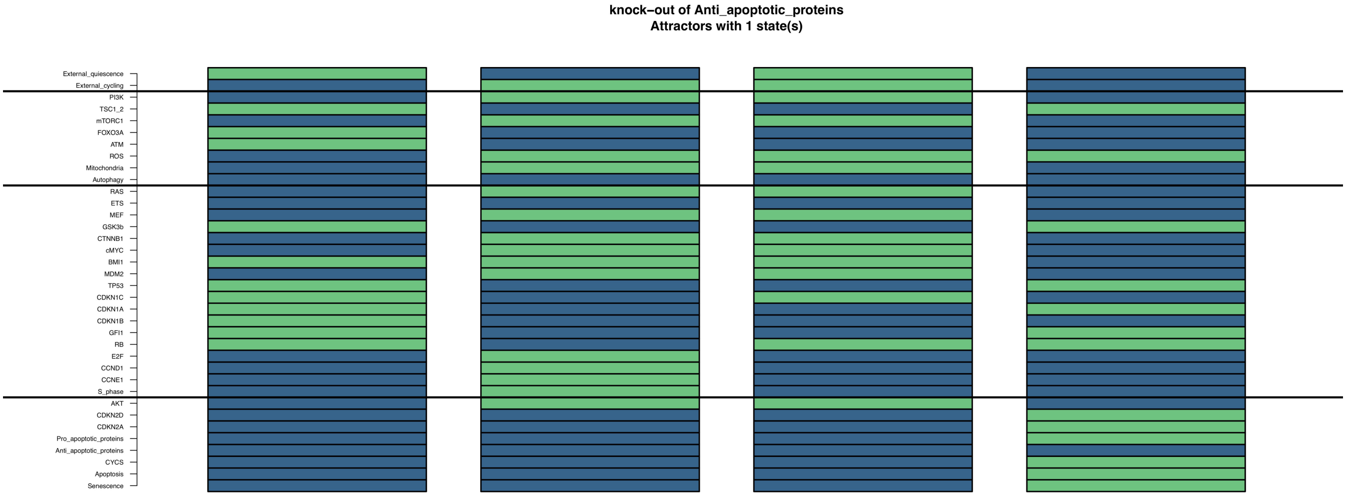
knock–out of Anti_apoptotic_proteins
Attractors with 1 state(s)
External_quiescence
External_cycling
PI3K
TSC1_2
mTORC1
FOXO3A
ATM
ROS
Mitochondria
Autophagy
RAS
ETS
MEF
GSK3b
CTNNB1
cMYC
BMI1
MDM2
TP53
CDKN1C
CDKN1A
CDKN1B
GFI1
RB
E2F
CCND1
CCNE1
S_phase
AKT
CDKN2D
CDKN2A
Pro_apoptotic_proteins
Anti_apoptotic_proteins
CYCS
Apoptosis
Senescence

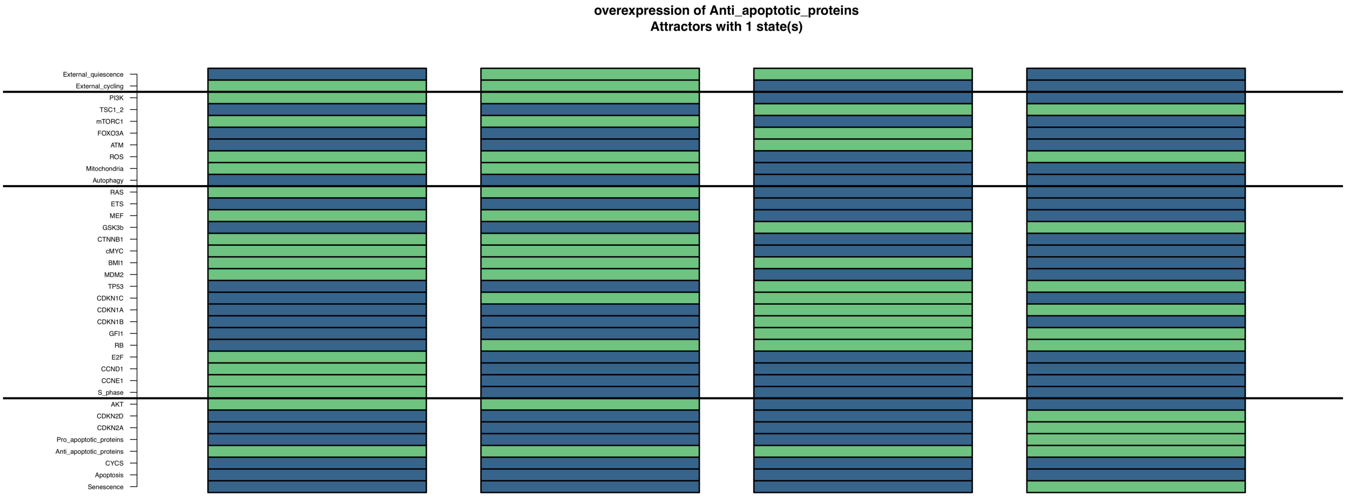
overexpression of Anti_apoptotic_proteins
Attractors with 1 state(s)
External_quiescence
External_cycling
PI3K
TSC1_2
mTORC1
FOXO3A
ATM
ROS
Mitochondria
Autophagy
RAS
ETS
MEF
GSK3b
CTNNB1
cMYC
BMI1
MDM2
TP53
CDKN1C
CDKN1A
CDKN1B
GFI1
RB
E2F
CCND1
CCNE1
S_phase
AKT
CDKN2D
CDKN2A
Pro_apoptotic_proteins
Anti_apoptotic_proteins
CYCS
Apoptosis
Senescence

knock–out of CYCS
Attractors with 1 state(s)

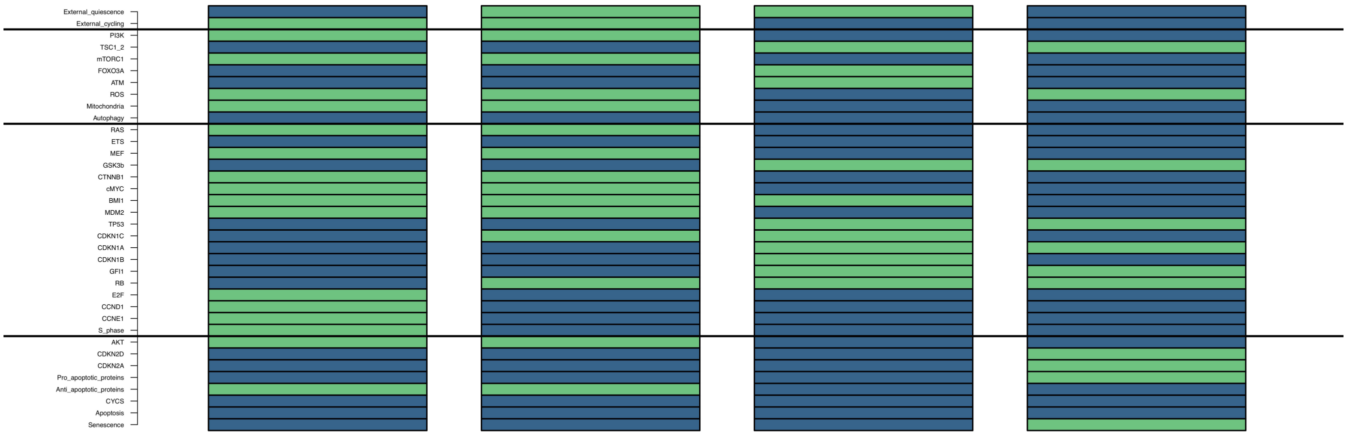

overexpression of CYCS
Attractors with 1 state(s)

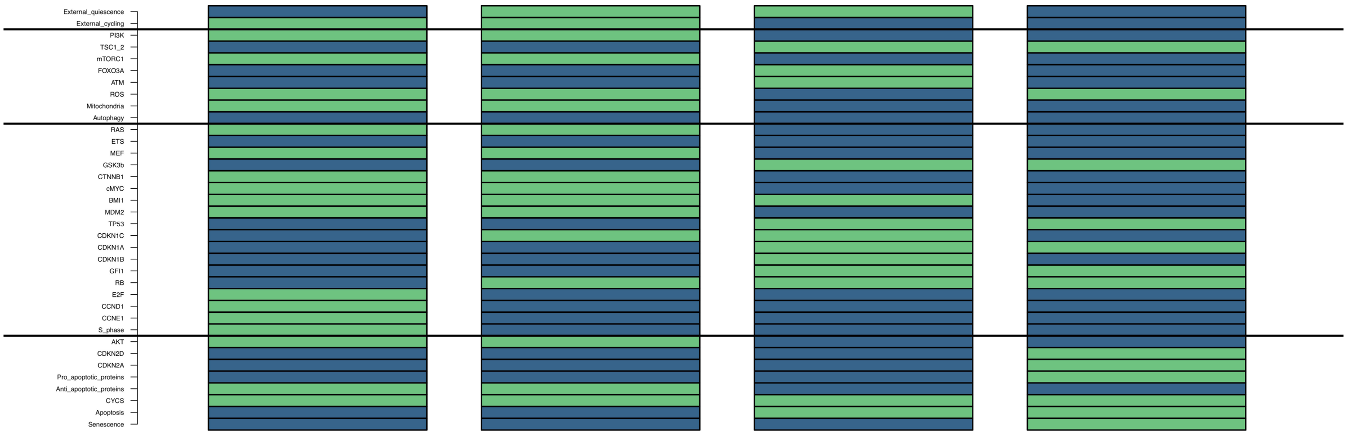

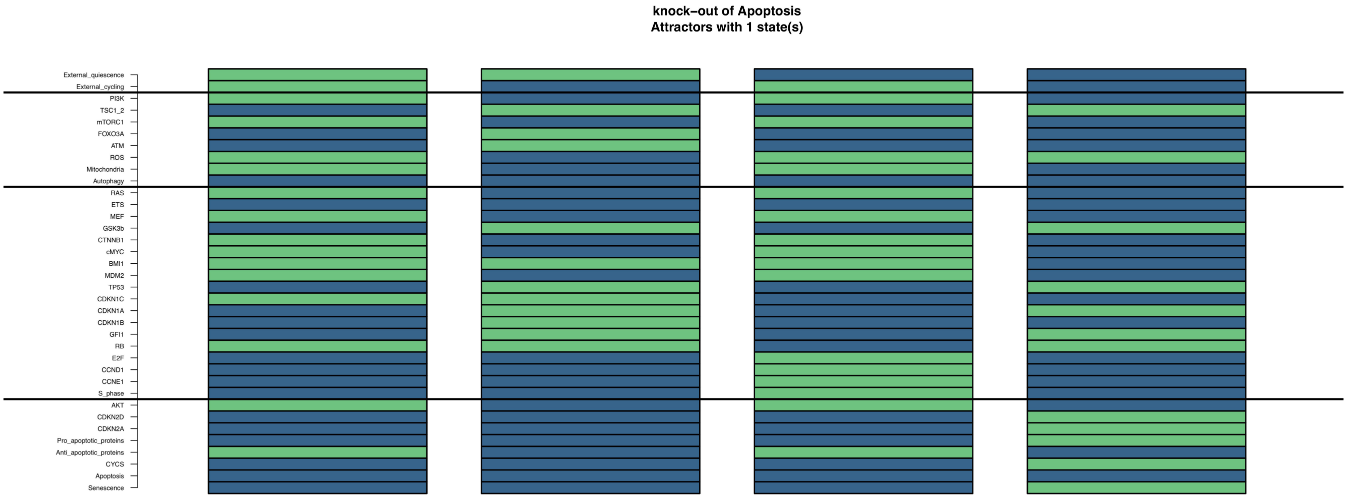

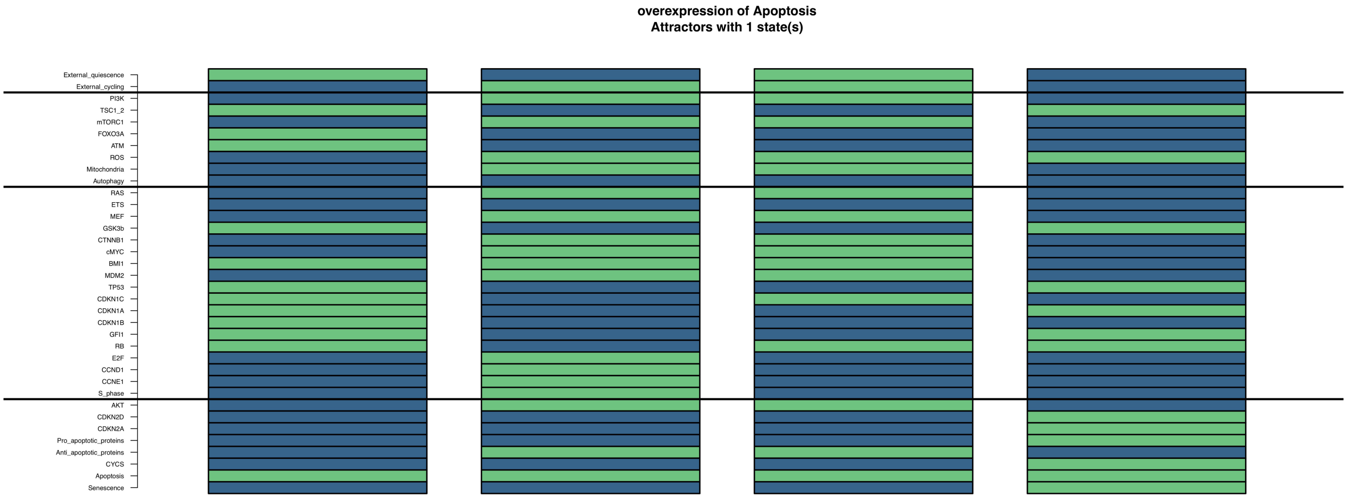

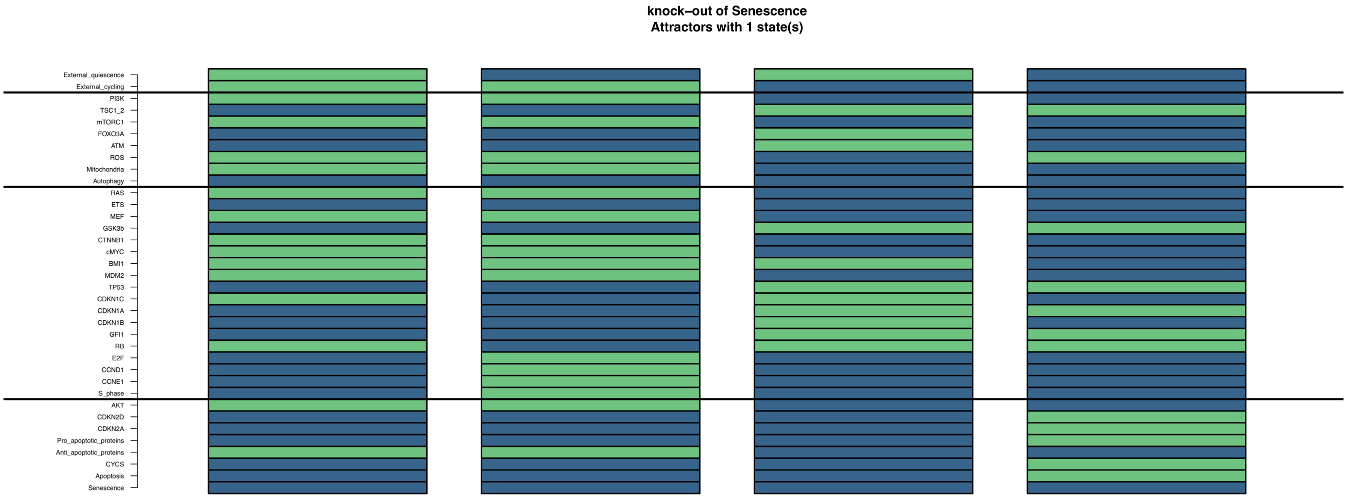

Figure S5: Attractor plots of each single-node-perturbation. The state of each component is depicted by colored rectangles. Green indicates active components; blue indicates inactive ones.

6 CODE

In the following we provide the code base for all simulations that have been performed for the manuscript and supplementary information. Additionally, we add the Boolean network model description in the BoolNet-format. All these files are also available via the github repository: https://github.com/sysbio-bioinf/HSC-boolean-network-model.

```
#Required library
library(BoolNet)
par(xpd = T)
par(mar = c(5,10,3,2))

#LoadNetwork
HSC <- loadNetwork("./HSC_RULES_FOR_SUBMISSION")

#Grouping of genes in the final table
groupingHSC<-list(class=rev(c("", "", "", "")),
                    index= rev(list(rev(c("External quiescence", "
                       External cycling")),
                                rev(c("PI3K", "TSC1_2", "mTORC1", "
                                   FOXO3A", "ATM", "ROS", "
                                   Mitochondria", "Autophagy")),
                                rev(c("RAS","ETS", "MEF", "GSK3b",
                                   "CTNNB1", "cMYC", "BMI1", "MDM2",
                                    "TP53", "CDKN1C", "CDKN1A", "
                                   CDKN1B", "GFI1", "RB", "E2F", "
                                   CCND1", "CCNE1", "S_phase")),
                                rev(c("AKT", "CDKN2D", "CDKN2A", "
                                   Pro_apoptotic_proteins", "Anti_
                                   apoptotic_proteins", "CYCS", "
                                   Apoptosis", "Senescence")))))


#######################Attractors for unperturbed Network
AllInAttr <- getAttractors(HSC, method="sat.exhaustive")
plotAttractors(AllInAttr, mode = "table", grouping = groupingHSC,
   reverse= T, onColor = "#6fc381", offColor = "steelblue4",
   drawLegend = FALSE, title = "Main phenotypes for HSC", allInOnePlot
    = T)


#####################Progressions towards entering of cell cycle:
   from LT to ST to cycling HSC
```

```
ProgressionFromLToSTHSC <- plotSequence (HSC, startState = c
   (1,1,0,1,0,1,1,0,0,0,0,0,0,1,0,0,1,0,1,1,1,1,1,1,0,0,0,0,0,0,0,0,0,0,0,0)
   , onColor = "#6fc381", offColor = "steelblue4", title = "From LT-
   HSC to activated ST-HSC" , drawLegend = "FALSE", grouping =
   groupingHSC, reverse= T)
ProgressionFromSTHSCtoCycling <-  plotSequence (HSC, grouping =
   groupingHSC, reverse = T, startState = c
   (0,1,1,0,1,0,0,1,1,0,1,0,1,0,1,1,1,1,0,1,0,0,0,1,0,0,0,0,1,0,0,0,1,0,0,0)
   , onColor = "#6fc381", offColor = "steelblue4", title = "From ST-
   HSC to cycling HSC" , drawLegend = "FALSE")
ProgressionFromLTHSCtoCycling <- plotSequence(HSC, startState = c
   (0,1,0,1,0,1,1,0,0,0,0,0,0,1,0,0,1,0,1,1,1,1,1,1,0,0,0,0,0,0,0,0,0,0,0,0)
   , onColor = "#6fc381", offColor = "steelblue4", title = "From LT-
   HSC to cycling HSC" , drawLegend = "FALSE", grouping = groupingHSC,
    reverse= T)


####################################simulations of K.O. models for Main
    Manuscript

#FOXO3A knockout
par(mar=c(3,12,2,2))
HSC_FOXO3A_LOSS <- fixGenes(HSC, "FOXO3A", 0)
AttrFOXO3A <- getAttractors(HSC_FOXO3A_LOSS, method="sat.exhaustive")
plotAttractors(AttrFOXO3A, grouping = groupingHSC, reverse = T,
   onColor = "#6fc381", offColor = "steelblue4", drawLegend = FALSE,
   title = "FOXO3A K.O.")

#ATM knockout

par(mar=c(3,12,2,2))
HSC_ATM_LOSS <- fixGenes(HSC, "ATM", 0)
AttrATM <- getAttractors(HSC_ATM_LOSS, method="sat.exhaustive")
plotAttractors(AttrATM, grouping = groupingHSC, reverse = T, onColor
   = "#6fc381", offColor = "steelblue4", drawLegend = FALSE, title = "
   ATM K.O.")

#BMI knockout

par(mar=c(3,12,2,2))
HSC_BMI1_LOSS <- fixGenes(HSC, "BMI1", 0)
AttrBMI1 <- getAttractors(HSC_BMI1_LOSS, method="sat.exhaustive")
plotAttractors(AttrBMI1, grouping = groupingHSC, reverse = T, onColor
    = "#6fc381", offColor = "steelblue4", drawLegend = FALSE, title =
   "BMI-1 K.O.")
```

```
#BMI AND P53 knockout

par(mar=c(2,10,3,1))
HSC_P53_LOSS <- fixGenes(HSC, "TP53", 0)
HSC_BMI_P53_LOSS <- fixGenes(HSC_P53_LOSS, "BMI1", 0 )
AttrP53BMI <- getAttractors(HSC_BMI_P53_LOSS, method="sat.exhaustive"
   )
plotAttractors(AttrP53BMI, grouping = groupingHSC, reverse = T,
   onColor = "#6fc381", offColor = "steelblue4", drawLegend = FALSE,
   title = "Resque of BMI-1 phenotype by loss of TP53")


#TP53 knockout

par(mar=c(2,10,3,1))
HSC_P53_LOSS <- fixGenes(HSC, "TP53", 0)
AttrP53 <- getAttractors(HSC_P53_LOSS, method = "sat.exhaustive")
plotAttractors(AttrP53, grouping = groupingHSC, reverse = T, onColor
   = "#6fc381", offColor = "steelblue4", drawLegend = FALSE, title = "
   p53 K.O.")

#MEF knockout

par(mar=c(2,10,3,1))
HSC_MEF_LOSS <- fixGenes(HSC, "MEF", 0)
Attr_MEF_LOSS <- getAttractors(HSC_MEF_LOSS, method= "sat.exhaustive"
   )
plotAttractors(Attr_MEF_LOSS, grouping = groupingHSC, reverse = T,
   onColor = "#6fc381", offColor = "steelblue4", drawLegend = FALSE,
   title = "MEF K.O.", allInOnePlot = T)



##########################Robustness analysis
#Hamming Distance
set.seed(10000)
Hamming<-testNetworkProperties(HSC, numRandomNets = 1000,
                               testFunction = "
                                  testTransitionRobustness",
                               testFunctionParams = list(numSamples
                                  =1000),
                               alternative="less")
```

```
############################ Cellcyclecheck

#plot attractor of the Faure cell cycle model
par(mar=c(2,10,3,1))
data("cellcycle")
attractorsCellCycle<- getAttractors(cellcycle)
plotAttractors(attractorsCellCycle, allInOnePlot = F, drawLegend = F,
    onColor = "#6fc381", offColor = "steelblue4")

#sequence to attractor by using the as startstrates the ones of our
   cycling HSC attractor
#nodes state: CyclinD=1, E2F=1, RB=0, P27=0, CyclinE=0)

plotSequence(cellcycle, startState = c(1,0,1,1,0,0,0,0,0,0), onColor
   = "#6fc381", offColor = "steelblue4", drawLegend = F)


############################# SINGLE-PETURBATION EXPERIMENTS
   #####################
net <- HSC
perturbation <- c(0,1)
names(perturbation) <- c("knock-out", "overexpression")

groupingHSC<-list(class=rev(c("", "", "", "")),
                  index= rev(list(rev(c("External_quiescence", "
                     External_cycling")),
                                 rev(c("PI3K", "TSC1_2", "mTORC1", "
                                    FOXO3A", "ATM","ROS","
                                    Mitochondria","Autophagy")),
                                 rev(c("RAS","ETS", "MEF", "GSK3b",
                                    "CTNNB1", "cMYC", "BMI1", "MDM2",
                                     "TP53", "CDKN1C", "CDKN1A", "
                                    CDKN1B", "GFI1", "RB", "E2F", "
                                    CCND1", "CCNE1", "S_phase")),
                                 rev(c("AKT", "CDKN2D", "CDKN2A", "
                                    Pro_apoptotic_proteins", "Anti_
                                    apoptotic_proteins", "CYCS", "
                                    Apoptosis", "Senescence")))))

for(g in seq_along(net$genes))
  for(p in perturbation)
  {
    tmp_net <- fixGenes(net, fixIndices = c(g), values=c(p))
    tmp_attr <- getAttractors(tmp_net,method = "sat.exhaustive")
    par(xpd = T)
```

```
    par(mar = c(5,10,3,2))
    par()
    plotAttractors(tmp_attr,
                   title = paste(names(perturbation)[p + 1], " of ",
                     net$genes[g], sep =""),
                   grouping = groupingHSC,
                   drawLegend = F,
                   onColor = "#6fc381", offColor = "steelblue4")
  }

########################### RAS HYPERACTIVATION DOWNREGULTES P53 IN
   ABSENCE OF EXTERNAL CYCLING ###############################
RAS_KI <- fixGenes(HSC, "RAS", 1)
RAS_KI_CYCLING_KO <- fixGenes(RAS_KI, "External_cycling" , 0 )
AttrRAS <- getAttractors(RAS_KI, method="sat.exhaustive")
AttrRAS_EXT <- getAttractors(RAS_KI_CYCLING_KO, method="sat.
   exhaustive")

#attractor for only RAS/PI3K consititutively active (here all
   populations are depicted)
plotAttractors(AttrRAS, grouping = groupingHSC, reverse = T, onColor
   = "#6fc381", offColor = "steelblue4", drawLegend = FALSE, title = "
   RAS K.I-")

#attractor for RAS/PI3K constitutively active and no presence at all
   of external cycling signals
plotAttractors(AttrRAS_EXT, grouping = groupingHSC, reverse = T,
   onColor = "#6fc381", offColor = "steelblue4", drawLegend = FALSE,
   title = "RAS K.I. and EXTERNAL K.O.")


############################### IN ABSENCE OF CYCLING SIGNALS CONST
   FOXO3A and ATM ACTIVATE TP53#################################
#absence of external cycling signals fixed
EXT_CYCLING_KO <- fixGenes(HSC, "External_cycling" , 0 )
#constitutive FOXO3A/ATM
FOXO3AKI <- fixGenes(EXT_CYCLING_KO, "FOXO3A", 1)
attrEXT_CYCLING <- getAttractors(EXT_CYCLING_KO, method="sat.
   exhaustive")
attrEXT_CYCLINg_FOXO3 <- getAttractors(FOXO3AKI, method="sat.
   exhaustive")

#attractor for only absence of external cycling (here all populations
     are depicted)
```

```
plotAttractors(attrEXT_CYCLING, grouping = groupingHSC, reverse = T,
   onColor = "#6fc381", offColor = "steelblue4", drawLegend = FALSE,
   title = "External cycling absence")
#acctractor for absence of external cycling signals and consitutive
   FOXO3A/ATM (results are similar dependening on TP53 activation)
plotAttractors(attrEXT_CYCLINg_FOXO3, grouping = groupingHSC, reverse
   = T, onColor = "#6fc381", offColor = "steelblue4", drawLegend =
   FALSE, title = "External cycling absence and constitutive FOXO3A")

############################### LOSS OF INTERACTION SIMULATION
   ######################
#we simulated loss of interaction mutants for analysis of the
   balancing effect of TP53 regulation via ATM and MEF
#Loss ATM : deleted interaction between ATM and MDM2
lossATM <- loadNetwork("./HSC_MODIFIED_ATM")
lossATMAttr <- getAttractors(lossATM, method ="sat.exhaustive")
plotAttractors(lossATMAttr, grouping = groupingHSC, reverse = T,
   onColor = "#6fc381", offColor = "steelblue4", drawLegend = FALSE,
   title = "Loss of Interaction ATM -> MDM2")
#Loss MEF : deleted interaction between MEF and MDM2
lossMEF <- loadNetwork(("./HSC_MODIFIED_MEF"))
lossMEFAttr <- getAttractors(lossATM, method ="sat.exhaustive")
plotAttractors(lossMEFAttr, grouping = groupingHSC, reverse = T,
   onColor = "#6fc381", offColor = "steelblue4", drawLegend = FALSE,
   title = "Loss of Interaction MEF -> MDM2")
#Loss Combo : both upper interactions removed at the same network
lossCombo <- loadNetwork(("./HSC_MODIFIED_Combo"))
lossComboAttr <- getAttractors(lossCombo, method ="sat.exhaustive")
plotAttractors(lossMEFAttr, grouping = groupingHSC, reverse = T,
   onColor = "#6fc381", offColor = "steelblue4", drawLegend = FALSE,
   title = "Loss of Interaction MEF -> MDM2 + ATM -> MDM2")
```

6.1 Original HSC network model

```
targets, factors

External_quiescence, External_quiescence
External_cycling, External_cycling
PI3K, RAS
TSC1_2, !AKT
mTORC1, !TSC1_2
FOXO3A, (External_quiescence) & !AKT
ATM, FOXO3A
ROS, Mitochondria | !ATM | !FOXO3A | !BMI1 | !TP53
Mitochondria, mTORC1
Autophagy, FOXO3A & ROS & !mTORC1
```

```
RAS, External_cycling
ETS, RAS & !MEF
MEF, RAS
GSK3b, !AKT
CTNNB1, !GSK3b
cMYC, CTNNB1 & !GSK3b
BMI1, cMYC | (ATM & FOXO3A)
MDM2, (TP53 | MEF) & !CDKN2D & !ATM
TP53, !MDM2
CDKN1C, External_quiescence | FOXO3A
CDKN1A, (TP53 | FOXO3A | External_quiescence |GFI1) & !cMYC
CDKN1B, FOXO3A
GFI1, TP53
RB, !CCND1 & !CCNE1
E2F, !RB & !GFI1
CCND1, !CDKN2A & !CDKN1C & cMYC
CCNE1, (!CDKN1C) & ((!CDKN1A & !CDKN1B) | CCND1) & (E2F)
S_phase, CCNE1 & E2F
AKT, PI3K
CDKN2D, !BMI1 & (E2F | ROS)
CDKN2A, (ETS | ROS) & !BMI1
Pro_apoptotic_proteins, TP53 & ROS & !AKT
Anti_apoptotic_proteins, (RAS | External_quiescence) & !GSK3b
CYCS, Pro_apoptotic_proteins & !Anti_apoptotic_proteins
Apoptosis, CYCS & !AKT
Senescence, (CDKN2A & ROS) | (TP53 & ROS & CDKN1A)
```

6.2 Loss of interaction network models

```
targets, factors

External_quiescence, External_quiescence
External_cycling, External_cycling
PI3K, RAS
TSC1_2, !AKT
mTORC1, !TSC1_2
FOXO3A, (External_quiescence) & !AKT
ATM, FOXO3A
ROS, Mitochondria | !ATM | !FOXO3A | !BMI1 | !TP53
Mitochondria, mTORC1
Autophagy, FOXO3A & ROS & !mTORC1
RAS, External_cycling
ETS, RAS & !MEF
MEF, RAS
GSK3b, !AKT
CTNNB1, !GSK3b
```

```
cMYC, CTNNB1 & !GSK3b
BMI1, cMYC | (ATM & FOXO3A)
MDM2, (TP53 | MEF) & !CDKN2D
TP53, !MDM2
CDKN1C, External_quiescence | FOXO3A
CDKN1A, (TP53 | FOXO3A | External_quiescence |GFI1) & !cMYC
CDKN1B, FOXO3A
GFI1, TP53
RB, !CCND1 & !CCNE1
E2F, !RB & !GFI1
CCND1, !CDKN2A & !CDKN1C & cMYC
CCNE1, (!CDKN1C) & ((!CDKN1A & !CDKN1B) | CCND1) & (E2F)
S_phase, CCNE1 & E2F
AKT, PI3K
CDKN2D, !BMI1 & (E2F | ROS)
CDKN2A, (ETS | ROS) & !BMI1
Pro_apoptotic_proteins, TP53 & ROS & !AKT
Anti_apoptotic_proteins, (RAS | External_quiescence) & !GSK3b
CYCS, Pro_apoptotic_proteins & !Anti_apoptotic_proteins
Apoptosis, CYCS & !AKT
Senescence, (CDKN2A & ROS) | (TP53 & ROS & CDKN1A)


targets, factors

External_quiescence, External_quiescence
External_cycling, External_cycling
PI3K, RAS
TSC1_2, !AKT
mTORC1, !TSC1_2
FOXO3A, (External_quiescence) & !AKT
ATM, FOXO3A
ROS, Mitochondria | !ATM | !FOXO3A | !BMI1 | !TP53
Mitochondria, mTORC1
Autophagy, FOXO3A & ROS & !mTORC1
RAS, External_cycling
ETS, RAS & !MEF
MEF, RAS
GSK3b, !AKT
CTNNB1, !GSK3b
cMYC, CTNNB1 & !GSK3b
BMI1, cMYC | (ATM & FOXO3A)
MDM2, (TP53) & !CDKN2D & !ATM
TP53, !MDM2
CDKN1C, External_quiescence | FOXO3A
CDKN1A, (TP53 | FOXO3A | External_quiescence |GFI1) & !cMYC
```

```
CDKN1B, FOXO3A
GFI1, TP53
RB, !CCND1 & !CCNE1
E2F, !RB & !GFI1
CCND1, !CDKN2A & !CDKN1C & cMYC
CCNE1, (!CDKN1C) & ((!CDKN1A & !CDKN1B) | CCND1) & (E2F)
S_phase, CCNE1 & E2F
AKT, PI3K
CDKN2D, !BMI1 & (E2F | ROS)
CDKN2A, (ETS | ROS) & !BMI1
Pro_apoptotic_proteins, TP53 & ROS & !AKT
Anti_apoptotic_proteins, (RAS | External_quiescence) & !GSK3b
CYCS, Pro_apoptotic_proteins & !Anti_apoptotic_proteins
Apoptosis, CYCS & !AKT
Senescence, (CDKN2A & ROS) | (TP53 & ROS & CDKN1A)


targets, factors

External_quiescence, External_quiescence
External_cycling, External_cycling
PI3K, RAS
TSC1_2, !AKT
mTORC1, !TSC1_2
FOXO3A, (External_quiescence) & !AKT
ATM, FOXO3A
ROS, Mitochondria | !ATM | !FOXO3A | !BMI1 | !TP53
Mitochondria, mTORC1
Autophagy, FOXO3A & ROS & !mTORC1
RAS, External_cycling
ETS, RAS & !MEF
MEF, RAS
GSK3b, !AKT
CTNNB1, !GSK3b
cMYC, CTNNB1 & !GSK3b
BMI1, cMYC | (ATM & FOXO3A)
MDM2, (TP53) & !CDKN2D
TP53, !MDM2
CDKN1C, External_quiescence | FOXO3A
CDKN1A, (TP53 | FOXO3A | External_quiescence |GFI1) & !cMYC
CDKN1B, FOXO3A
GFI1, TP53
RB, !CCND1 & !CCNE1
E2F, !RB & !GFI1
CCND1, !CDKN2A & !CDKN1C & cMYC
CCNE1, (!CDKN1C) & ((!CDKN1A & !CDKN1B) | CCND1) & (E2F)
```

```
S_phase, CCNE1 & E2F
AKT, PI3K
CDKN2D, !BMI1 & (E2F | ROS)
CDKN2A, (ETS | ROS) & !BMI1
Pro_apoptotic_proteins, TP53 & ROS & !AKT
Anti_apoptotic_proteins, (RAS | External_quiescence) & !GSK3b
CYCS, Pro_apoptotic_proteins & !Anti_apoptotic_proteins
Apoptosis, CYCS & !AKT
Senescence, (CDKN2A & ROS) | (TP53 & ROS & CDKN1A)
```

REFERENCES

Blank U, Karlsson S. TGF-β signaling in the control of hematopoietic stem cells. *Blood* **125** (2015) 3542–3550. doi:10.1182/blood-2014-12-618090.

Pietras EM, Warr MR, Passegué E. Cell cycle regulation in hematopoietic stem cells. *Journal of Cell Biology* **195** (2011a) 709–720. doi:10.1083/jcb.201102131.

Eliasson P, Jönsson JI. The hematopoietic stem cell niche: low in oxygen but a nice place to be. *Journal of Cellular Physiology* **222** (2010) 17–22. doi:10.1002/jcp.21908.

Piccoli C, Agriesti F, Scrima R, Falzetti F, Di Ianni M, Capitanio N. To breathe or not to breathe: the haematopoietic stem/progenitor cells dilemma. *British journal of pharmacology* **169** (2013) 1652–1671. doi:10.1111/bph.12253.

Scandura JM, Boccuni P, Massagué J, Nimer SD. Transforming growth factor β-induced cell cycle arrest of human hematopoietic cells requires p57KIP2 up-regulation. *Proceedings of the National Academy of Sciences* **101** (2004) 15231–15236. doi:10.1073/pnas.0406771101.

Suda T, Takubo K, Semenza GL. Metabolic regulation of hematopoietic stem cells in the hypoxic niche. *Cell Stem Cell* **9** (2011a) 298–310. doi:10.1016/j.stem.2011.09.010.

Ema H, Takano H, Sudo K, Nakauchi H. In vitro self-renewal division of hematopoietic stem cells. *The Journal of Experimental Medicine* **192** (2000) 1281–1288. doi:10.1084/jem.192.9.1281.

Kaushansky K. Molecular mechanisms of thrombopoietin signaling. *Journal of Thrombosis and Haemostasis* **7** (2009) 235–238. doi:10.1111/j.1538-7836.2009.03419.x.

Kumar S, Geiger H. HSC Niche Biology and HSC Expansion Ex Vivo. *Trends in Molecular Medicine* **23** (2017) 799–819. doi:10.1016/j.molmed.2017.07.003.

Lee D, Kim DW, Cho JY. Role of growth factors in hematopoietic stem cell niche. *Cell Biology and Toxicology* **87** (2020) 2162. doi:10.1007/s10565-019-09510-7.

Martelli AM, Evangelisti C, Chiarini F, Grimaldi C, Cappellini A, Ognibene A, et al. The emerging role of the phosphatidylinositol 3-kinase/Akt/mammalian target of rapamycin signaling network in normal myelopoiesis and leukemogenesis. *Biochimica et Biophysica Acta* **1803** (2010) 991–1002. doi:10.1016/j.bbamcr.2010.04.005.

Pinho S, Frenette PS. Haematopoietic stem cell activity and interactions with the niche. *Nature Reviews Molecular Cell Biology* **20** (2019) 303–320. doi:10.1038/s41580-019-0103-9.

Varghese LN, Defour JP, Pecquet C, Constantinescu SN. The Thrombopoietin Receptor: Structural Basis of Traffic and Activation by Ligand, Mutations, Agonists, and Mutated Calreticulin. *Frontiers in Endocrinology* **8** (2017) 59. doi:10.3389/fendo.2017.00059.

Chung E, Hsu CL, Kondo M. Constitutive MAP kinase activation in hematopoietic stem cells induces a myeloproliferative disorder. *PLOS ONE* **6** (2011) e28350. doi:10.1371/journal.pone.0028350.

Hemmati S, Sinclair T, Tong M, Bartholdy B, Okabe RO, Ames K, et al. PI3K alpha and delta promote hematopoietic stem cell activation. *JCI Insight* **4** (2019).

Warr MR, Pietras EM, Passegué E. Mechanisms controlling hematopoietic stem cell functions during normal hematopoiesis and hematological malignancies. *WIREs Systems Biology and Medicine* **3** (2011) 681–701. doi:10.1002/wsbm.145.

Ludin A, Gur-Cohen S, Golan K, Kaufmann KB, Itkin T, Medaglia C, et al. Reactive oxygen species regulate hematopoietic stem cell self-renewal, migration and development, as well as their bone marrow microenvironment. *Antioxidants & Redox Signaling* **21** (2014a) 1605–1619. doi:10.1089/ars.2014.5941.

Bakker WJ, Harris IS, Mak TW. FOXO3a Is Activated in Response to Hypoxic Stress and Inhibits HIF1-Induced Apoptosis via Regulation of CITED2. *Molecular Cell* **28** (2007) 941–953. doi:10.1016/j.molcel.2007.10.035.

Brunet A, Bonni A, Zigmond MJ, Lin MZ, Juo P, Hu LS, et al. Akt promotes cell survival by phosphorylating and inhibiting a Forkhead transcription factor. *Cell* **96** (1999) 857–868. doi:10.1016/s0092-8674(00)80595-4.

Brunet A, Kanai F, Stehn J, Xu J, Sarbassova D, Frangioni JV, et al. 14-3-3 transits to the nucleus and participates in dynamic nucleocytoplasmic transport. *The Journal of Cell Biology* **156** (2002) 817–828. doi:10.1083/jcb.200112059.

Storz P. Forkhead homeobox type O transcription factors in the responses to oxidative stress. *Antioxidants & Redox Signaling* **14** (2011) 593–605. doi:10.1089/ars.2010.3405.

Yalcin S, Zhang X, Luciano JP, Mungamuri SK, Marinkovic D, Vercherat C, et al. Foxo3 is essential for the regulation of ataxia telangiectasia mutated and oxidative stress-mediated homeostasis of hematopoietic stem cells. *Journal of Biological Chemistry* **283** (2008) 25692–25705. doi:10.1074/jbc.M800517200.

Chen C, Liu Y, Liu R, Ikenoue T, Guan KL, Liu Y, et al. TSC–mTOR maintains quiescence and function of hematopoietic stem cells by repressing mitochondrial biogenesis and reactive oxygen species . *Journal of Experimental Medicine* **205** (2008) 2397–2408. doi:10.1084/jem.20081297.

Gan B, Sahin E, Jiang S, Sanchez-Aguilera A, Scott KL, Chin L, et al. mTORC1-dependent and -independent regulation of stem cell renewal, differentiation, and mobilization. *Proceedings of the National Academy of Sciences* **105** (2008) 19384–19389. doi:10.1073/pnas.0810584105.

Juntilla MM, Patil VD, Calamito M, Joshi RP, Birnbaum MJ, Koretzky GA. AKT1 and AKT2 maintain hematopoietic stem cell function by regulating reactive oxygen species. *Blood* **115** (2010) 4030–4038. doi:10.1182/blood-2009-09-241000.

Yilmaz OH, Valdez R, Theisen BK, Guo W, Ferguson DO, Wu H, et al. Pten dependence distinguishes haematopoietic stem cells from leukaemia-initiating cells. *Nature* **441** (2006) 475–482. doi:10.1038/nature04703.

Abbas HA, Pant V, Lozano G. The ups and downs of p53 regulation in hematopoietic stem cells. *Cell Cycle* **10** (2011) 3257–3262. doi:10.4161/cc.10.19.17721.

Ito K, Hirao A, Arai F, Matsuoka S, Takubo K, Hamaguchi I, et al. Regulation of oxidative stress by ATM is required for self-renewal of haematopoietic stem cells. *Nature* **431** (2004) 997–1002. doi:10.1038/nature02989.

Ito K, Hirao A, Arai F, Takubo K, Matsuoka S, Miyamoto K, et al. Reactive oxygen species act through p38 MAPK to limit the lifespan of hematopoietic stem cells. *Nature Medicine* **12** (2006) 446–451. doi:10.1038/nm1388.

Kharas MG, Okabe R, Ganis JJ, Gozo M, Khandan T, Paktinat M, et al. Constitutively active AKT depletes hematopoietic stem cells and induces leukemia in mice. *Blood* **115** (2010) 1406–1415. doi:10.1182/blood-2009-06-229443.

Liu J, Cao L, Chen J, Song S, Lee IH, Quijano C, et al. Bmi1 regulates mitochondrial function and the DNA damage response pathway. *Nature* **459** (2009a) 387–392. doi:10.1038/nature08040.

Miyamoto K, Araki KY, Naka K, Arai F, Takubo K, Yamazaki S, et al. Foxo3a is essential for maintenance of the hematopoietic stem cell pool. *Cell Stem Cell* **1** (2007) 101–112. doi:10.1016/j.stem.2007.02.001.

Park IK, Qian D, Kiel M, Becker MW, Pihalja M, Weissman IL, et al. Bmi-1 is required for maintenance of adult self-renewing haematopoietic stem cells. *Nature* **423** (2003) 302–305. doi:10.1038/nature01587.

Rizo A, Olthof S, Han L, Vellenga E, de Haan G, Schuringa JJ. Repression of BMI1 in normal and leukemic human CD34+ cells impairs self-renewal and induces apoptosis. *Blood, The Journal of the American Society of Hematology* **114** (2009) 1498–1505. doi:10.1182/blood-2009-03-209734.

Schuringa JJ, Vellenga E. Role of the polycomb group gene BMI1 in normal and leukemic hematopoietic stem and progenitor cells. *Current Opinion in Hematology* **17** (2010) 294–299. doi:10.1097/MOH. 0b013e328338c439.

Tothova Z, Gilliland DG. FoxO transcription factors and stem cell homeostasis: insights from the hematopoietic system. *Cell Stem Cell* **1** (2007) 140–152. doi:10.1016/j.stem.2007.07.017.

Tothova Z, Kollipara R, Huntly BJ, Lee BH, Castrillon DH, Cullen DE, et al. FoxOs are critical mediators of hematopoietic stem cell resistance to physiologic oxidative stress. *Cell* **128** (2007) 325–339. doi:10. 1016/j.cell.2007.01.003.

Liu B, Chen Y, Clair DKS. Ros and p53: a versatile partnership. *Free Radical Biology and Medicine* **44** (2008) 1529–1535. doi:https://doi.org/10.1016/j.freeradbiomed.2008.01.011.

Mortensen M, Watson AS, Simon AK. Lack of autophagy in the hematopoietic system leads to loss of hematopoietic stem cell function and dysregulated myeloid proliferation. *Autophagy* **7** (2011) 1069–1070.

Warr MR, Binnewies M, Flach J, Reynaud D, Garg T, Malhotra R, et al. FOXO3A directs a protective autophagy program in haematopoietic stem cells. *Nature* **494** (2013a) 323–327. doi:10.1038/ nature11895.

Warr MR, Kohli L, Passegué E. Born to survive: autophagy in hematopoietic stem cell maintenance. *Cell Cycle* **12** (2013b) 1979–1980. doi:10.4161/cc.25303.

Lacorazza HD, Yamada T, Liu Y, Miyata Y, Sivina M, Nunes J, et al. The transcription factor MEF/ELF4 regulates the quiescence of primitive hematopoietic cells. *Cancer Cell* **9** (2006) 175–187. doi:10.1016/j. ccr.2006.02.017.

Liu Y, Elf SE, Miyata Y, Sashida G, Liu Y, Huang G, et al. p53 regulates hematopoietic stem cell quiescence. *Cell stem cell* **4** (2009b) 37–48. doi:10.1016/j.stem.2008.11.006.

Sashida G, Liu Y, Elf S, Miyata Y, Ohyashiki K, Izumi M, et al. ELF4/MEF activates MDM2 expression and blocks oncogene-induced p16 activation to promote transformation. *Molecular and Cellular Biology* **29** (2009) 3687–3699. doi:10.1128/MCB.01551-08.

Massagué J. G1 cell-cycle control and cancer. *Nature* **432** (2004) 298–306. doi:10.1038/nature03094.

Dolnikov A, Xu N, Shen S, Song E, Holmes T, Klamer G, et al. GSK-3β inhibition promotes early engraftment of ex vivo-expanded haematopoietic stem cells. *Cell Proliferation* **47** (2014) 113–123. doi:10.1111/cpr.12092.

Holmes T, O'Brien TA, Knight R, Lindeman R, Shen S, Song E, et al. Glycogen synthase kinase-3beta inhibition preserves hematopoietic stem cell activity and inhibits leukemic cell growth. *Stem Cells* **26** (2008) 1288–1297. doi:10.1634/stemcells.2007-0600.

Huang J, Zhang Y, Bersenev A, O'Brien WT, Tong W, Emerson SG, et al. Pivotal role for glycogen synthase kinase–3 in hematopoietic stem cell homeostasis in mice. *The Journal of Clinical Investigation* **119** (2009) 3519–3529. doi:10.1172/JCI40572.

McCubrey JA, Steelman LS, Bertrand FE, Davis NM, Abrams SL, Montalto G, et al. Multifaceted roles of GSK-3 and Wnt/β-catenin in hematopoiesis and leukemogenesis: opportunities for therapeutic intervention. *Leukemia* **28** (2014) 15–33. doi:10.1038/leu.2013.184.

Robertson H, Hayes JD, Sutherland C. A partnership with the proteasome; the destructive nature of GSK3. *Biochemical Pharmacology* **147** (2018) 77–92. doi:10.1016/j.bcp.2017.10.016.

Maurer U, Preiss F, Brauns-Schubert P, Schlicher L, Charvet C. GSK-3 - at the crossroads of cell death and survival. *Journal of Cell Science* **127** (2014) 1369–1378. doi:10.1242/jcs.138057.

Murphy MJ, Wilson A, Trumpp A. More than just proliferation: Myc function in stem cells. *Trends in Cell Biology* **15** (2005) 128–137. doi:10.1016/j.tcb.2005.01.008.

Wilson A, Murphy MJ, Oskarsson T, Kaloulis K, Bettess MD, Oser GM, et al. c-Myc controls the balance between hematopoietic stem cell self-renewal and differentiation. *Genes & Development* **18** (2004a) 2747–2763. doi:10.1101/gad.313104.

Xu C, Kim NG, Gumbiner BM. Regulation of protein stability by GSK3 mediated phosphorylation. *Cell Cycle* **8** (2009) 4032–4039. doi:10.4161/cc.8.24.10111.

Guney I, Wu S, Sedivy JM. Reduced c-myc signaling triggers telomere-independent senescence by regulating bmi-1 and p16ink4a. *Proceedings of the National Academy of Sciences* **103** (2006) 3645–3650. doi:https://doi.org/10.1073/pnas.0600069103.

Jung JW, Lee S, Seo MS, Park SB, Kurtz A, Kang SK, et al. Histone deacetylase controls adult stem cell aging by balancing the expression of polycomb genes and jumonji domain containing 3. *Cellular and Molecular Life Sciences* **67** (2010) 1165–1176. doi:https://doi.org/10.1007/s00018-009-0242-9.

Kim J, Hwangbo J, Wong PKY. p38 MAPK-Mediated Bmi-1 down-regulation and defective proliferation in ATM-deficient neural stem cells can be restored by Akt activation. *PLOS ONE* **6** (2011) e16615. doi:10.1371/journal.pone.0016615.

Passegué E, Wagers AJ, Giuriato S, Anderson WC, Weissman IL. Global analysis of proliferation and cell cycle gene expression in the regulation of hematopoietic stem and progenitor cell fates. *The Journal of Experimental Medicine* **202** (2005) 1599–1611. doi:10.1084/jem.20050967.

Rayess H, Wang MB, Srivatsan ES. Cellular senescence and tumor suppressor gene p16. *International Journal of Cancer* **130** (2012) 1715–1725. doi:10.1002/ijc.27316.

Rizo A, Vellenga E, de Haan G, Schuringa JJ. Signaling pathways in self-renewing hematopoietic and leukemic stem cells: do all stem cells need a niche? *Human Molecular Genetics* **15 Spec No 2** (2006) R210–9. doi:10.1093/hmg/ddl175.

Harris SL, Levine AJ. The p53 pathway: positive and negative feedback loops. *Oncogene* **24** (2005) 2899–2908. doi:10.1038/sj.onc.1208615.

Haupt Y, Maya R, Kazaz A, Oren M. Mdm2 promotes the rapid degradation of p53. *Nature* **387** (1997) 296–299. doi:10.1038/387296a0.

Honda R, Tanaka H, Yasuda H. Oncoprotein MDM2 is a ubiquitin ligase E3 for tumor suppressor p53. *FEBS Letters* **420** (1997) 25–27. doi:10.1016/s0014-5793(97)01480-4.

Kastan MB, Lim DS, Kim ST, Xu B, Canman C. Multiple signaling pathways involving ATM. *Cold Spring Harbor Symposia on Quantitative Biology* (Cold Spring Harbor Laboratory Press) (2000), 521–526. doi:10.1101/sqb.2000.65.521.

Kubbutat MH, Jones SN, Vousden KH. Regulation of p53 stability by Mdm2. *Nature* **387** (1997) 299–303. doi:10.1038/387299a0.

Lowe SW, Sherr CJ. Tumor suppression by Ink4a-Arf: progress and puzzles. *Current Opinion in Genetics & Development* **13** (2003) 77–83. doi:10.1016/s0959-437x(02)00013-8.

Maya R, Balass M, Kim ST, Shkedy D, Leal JFM, Shifman O, et al. ATM-dependent phosphorylation of Mdm2 on serine 395: role in p53 activation by DNA damage. *Genes & Development* **15** (2001) 1067–1077. doi:10.1101/gad.886901.

Meulmeester E, Pereg Y, Shiloh Y, Jochemsen AG. Atm-mediated phosphorylations inhibit mdmx/mdm2 stabilization by hausp in favor of p53 activation. *Cell Cycle* **4** (2005) 1166–1170. doi:https://doi.org/10.4161/cc.4.9.1981.

Momand J, Zambetti GP, Olson DC, George D, Levine AJ. The mdm-2 oncogene product forms a complex with the p53 protein and inhibits p53-mediated transactivation. *Cell* **69** (1992) 1237–1245. doi:10.1016/0092-8674(92)90644-r.

Pant V, Quintás-Cardama A, Lozano G. The p53 pathway in hematopoiesis: lessons from mouse models, implications for humans. *Blood* **120** (2012a) 5118–5127. doi:10.1182/blood-2012-05-356014.

Perry ME. The regulation of the p53-mediated stress response by MDM2 and MDM4. *Cold Spring Harbor Perspectives in Biology* **2** (2010) a000968. doi:10.1101/cshperspect.a000968.

Sherr CJ, Weber JD. The ARF/p53 pathway. *Current Opinion in Genetics & Development* **10** (2000) 94–99. doi:10.1016/s0959-437x(99)00038-6.

Sherr CJ. The INK4a/ARF network in tumour suppression. *Nature Reviews Molecular Cell Biology* **2** (2001) 731–737. doi:10.1038/35096061.

Shvarts A, Steegenga WT, Riteco N, Van Laar T, Dekker P, Bazuine M, et al. MDMX: a novel p53-binding protein with some functional properties of MDM2. *The EMBO Journal* **15** (1996) 5349–5357.

Abbas HA, Maccio DR, Coskun S, Jackson JG, Hazen AL, Sills TM, et al. Mdm2 is required for survival of hematopoietic stem cells/progenitors via dampening of ROS-induced p53 activity. *Cell Stem Cell* **7** (2010) 606–617. doi:10.1016/j.stem.2010.09.013.

Yamazaki S, Iwama A, Takayanagi Si, Morita Y, Eto K, Ema H, et al. Cytokine signals modulated via lipid rafts mimic niche signals and induce hibernation in hematopoietic stem cells. *The EMBO Journal* **25** (2006) 3515–3523. doi:10.1038/sj.emboj.7601236.

Yamazaki S, Iwama A, Morita Y, Eto K, Ema H, Nakauchi H. Cytokine signaling, lipid raft clustering, and HSC hibernation. *Annals of the New York Academy of Sciences* **1106** (2007) 54–63. doi:10.1196/annals.1392.017.

Asai T, Liu Y, Bae N, Nimer SD. The p53 tumor suppressor protein regulates hematopoietic stem cell fate. *Journal of Cellular Physiology* **226** (2011) 2215–2221. doi:https://doi.org/10.4161/cc.8.19.9627.

Baena E, Ortiz M, Martínez-A C, de Alborán IM. c-Myc is essential for hematopoietic stem cell differentiation and regulates Lin(-)Sca-1(+)c-Kit(-) cell generation through p21. *Experimental Hematology* **35** (2007) 1333–1343. doi:10.1016/j.exphem.2007.05.015.

El-Deiry WS. p21/p53, cellular growth control and genomic integrity. *Cyclin Dependent Kinase (CDK) Inhibitors* (Springer) (1998), 121–137. doi:https://doi.org/10.1007/978-3-642-71941-7-6.

Eliasson P, Rehn M, Hammar P, Larsson P, Sirenko O, Flippin LA, et al. Hypoxia mediates low cell-cycle activity and increases the proportion of long-term-reconstituting hematopoietic stem cells during in vitro culture. *Experimental Hematology* **38** (2010) 301–310.e2. doi:10.1016/j.exphem.2010.01.005.

Hock H, Hamblen MJ, Rooke HM, Schindler JW, Saleque S, Fujiwara Y, et al. Gfi-1 restricts proliferation and preserves functional integrity of haematopoietic stem cells. *Nature* **431** (2004) 1002–1007. doi:10.1038/nature02994.

Tran H, Brunet A, Griffith EC, Greenberg ME. The many forks in FOXO's road. *Science's STKE* **2003** (2003) re5. doi:10.1126/stke.2003.172.re5.

Vivanco I, Sawyers CL. The phosphatidylinositol 3-kinase–AKT pathway in human cancer. *Nature Reviews Cancer* **2** (2002) 489–501. doi:10.1038/nrc839.

Zeng H, Yücel R, Kosan C, Klein-Hitpass L, Möröy T. Transcription factor Gfi1 regulates self-renewal and engraftment of hematopoietic stem cells. *The EMBO Journal* **23** (2004) 4116–4125. doi:10.1038/sj.emboj.7600419.

Sherr CJ, Roberts JM. CDK inhibitors: positive and negative regulators of G1-phase progression. *Genes & Development* **13** (1999) 1501–1512. doi:10.1101/gad.13.12.1501.

Tesio M, Trumpp A. Breaking the cell cycle of HSCs by p57 and friends. *Cell Stem Cell* **9** (2011a) 187–192. doi:10.1016/j.stem.2011.08.005.

Giacinti C, Giordano A. RB and cell cycle progression. *Oncogene* **25** (2006) 5220–5227. doi:10.1038/sj.onc.1209615.

Bowie MB, Kent DG, Dykstra B, McKnight KD, McCaffrey L, Hoodless PA, et al. Identification of a new intrinsically timed developmental checkpoint that reprograms key hematopoietic stem cell properties. *Proceedings of the National Academy of Sciences* **104** (2007) 5878–5882. doi:10.1073/pnas.0700460104.

Matsumoto A, Takeishi S, Kanie T, Susaki E, Onoyama I, Tateishi Y, et al. p57 is required for quiescence and maintenance of adult hematopoietic stem cells. *Cell Stem Cell* **9** (2011a) 262–271. doi:10.1016/j.stem.2011.06.014.

Satoh Y, Matsumura I, Tanaka H, Ezoe S, Sugahara H, Mizuki M, et al. Roles for c-myc in self-renewal of hematopoietic stem cells. *Journal of Biological Chemistry* **279** (2004) 24986–24993. doi:10.1074/jbc.M400407200.

Zou P, Yoshihara H, Hosokawa K, Tai I, Shinmyozu K, Tsukahara F, et al. p57Kip2 and p27Kip1 cooperate to maintain hematopoietic stem cell quiescence through interactions with Hsc70. *Cell Stem Cell* **9** (2011a) 247–261. doi:10.1016/j.stem.2011.07.003.

Cheng T, Rodrigues N, Dombkowski D, Stier S, Scadden DT. Stem cell repopulation efficiency but not pool size is governed by p27(kip1). *Nature Medicine* **6** (2000a) 1235–1240. doi:10.1038/81335.

Cheng T, Rodrigues N, Shen H, Yang Yg, Dombkowski D, Sykes M, et al. Hematopoietic stem cell quiescence maintained by p21cip1/waf1. *Science* **287** (2000b) 1804–1808. doi:10.1126/science.287.5459.1804.

Cheng T, Shen H, Rodrigues N, Stier S, Scadden DT. Transforming growth factor β1 mediates cell-cycle arrest of primitive hematopoietic cells independent of p21Cip1/Waf1 or p27Kip1. *Blood* **98** (2001) 3643–3649. doi:10.1182/blood.v98.13.3643.

Foudi A, Hochedlinger K, Van Buren D, Schindler JW, Jaenisch R, Carey V, et al. Analysis of histone 2B-GFP retention reveals slowly cycling hematopoietic stem cells. *Nature Biotechnology* **27** (2009) 84–90. doi:10.1038/nbt.1517.

van Os R, Kamminga LM, Ausema A, Bystrykh LV, Draijer DP, Van Pelt K, et al. A Limited role for p21Cip1/Waf1 in maintaining normal hematopoietic stem cell functioning. *Stem Cells* **25** (2007) 836–843. doi:10.1634/stemcells.2006-0631.

Hao S, Chen C, Cheng T. Cell cycle regulation of hematopoietic stem or progenitor cells. *International Journal of Hematology* **103** (2016) 487–497. doi:10.1007/s12185-016-1984-4.

Jacobs JJ, Kieboom K, Marino S, DePinho RA, van Lohuizen M. The oncogene and Polycomb-group gene bmi-1 regulates cell proliferation and senescence through the ink4a locus. *Nature* **397** (1999) 164–168. doi:10.1038/16476.

Lessard J, Sauvageau G. Bmi-1 determines the proliferative capacity of normal and leukaemic stem cells. *Nature* **423** (2003) 255–260. doi:10.1038/nature01572.

Macleod KF. The role of the RB tumour suppressor pathway in oxidative stress responses in the haematopoietic system. *Nature Reviews Cancer* **8** (2008) 769–781. doi:10.1038/nrc2504.

Muller M. Cellular senescence: molecular mechanisms, in vivo significance, and redox considerations. *Antioxidants & Redox Signaling* **11** (2009) 59–98. doi:10.1089/ars.2008.2104.

Serrano M, Lin AW, McCurrach ME, Beach D, Lowe SW. Oncogenic ras provokes premature cell senescence associated with accumulation of p53 and p16INK4a. *Cell* **88** (1997) 593–602. doi:10.1016/s0092-8674(00)81902-9.

Zhu JW, DeRyckere D, Li FX, Wan YY, DeGregori J. A role for E2F1 in the induction of ARF, p53, and apoptosis during thymic negative selection. *Cell Growth & Differentiation* **10** (1999) 829–838.

Kennedy SG, Kandel ES, Cross TK, Hay N. Akt/Protein kinase B inhibits cell death by preventing the release of cytochrome c from mitochondria. *Molecular and Cellular Biology* **19** (1999) 5800–5810. doi:10.1128/mcb.19.8.5800.

Nii T, Marumoto T, Tani K. Roles of p53 in various biological aspects of hematopoietic stem cells. *Journal of Biomedicine and Biotechnology* **2012** (2012) 903435–10. doi:10.1155/2012/903435.

Redza-Dutordoir M, Averill-Bates DA. Activation of apoptosis signalling pathways by reactive oxygen species. *Biochimica et Biophysica Acta* **1863** (2016) 2977–2992. doi:10.1016/j.bbamcr.2016.09.012.

Song G, Ouyang G, Bao S. The activation of Akt/PKB signaling pathway and cell survival. *Journal of Cellular and Molecular Medicine* **9** (2005) 59–71. doi:10.1111/j.1582-4934.2005.tb00337.x.

Butler JM, Nolan DJ, Vertes EL, Varnum-Finney B, Kobayashi H, Hooper AT, et al. Endothelial cells are essential for the self-renewal and repopulation of Notch-dependent hematopoietic stem cells. *Cell Stem Cell* **6** (2010) 251–264. doi:10.1016/j.stem.2010.02.001.

Gerber HP, Malik AK, Solar GP, Sherman D, Liang XH, Meng G, et al. VEGF regulates haematopoietic stem cell survival by an internal autocrine loop mechanism. *Nature* **417** (2002) 954–958. doi:10.1038/nature00821.

Hannum C, Culpepper J, Campbell D, McClanahan T, Zurawski S, Bazan JF, et al. Ligand for FLT3/FLK2 receptor tyrosine kinase regulates growth of haematopoietic stem cells and is encoded by variant RNAs. *Nature* **368** (1994) 643–648. doi:10.1038/368643a0.

Kollek M, Müller A, Egle A, Erlacher M. Bcl-2 proteins in development, health, and disease of the hematopoietic system. *The FEBS Journal* **283** (2016) 2779–2810. doi:10.1111/febs.13683.

Mojsa B, Lassot I, Desagher S. Mcl-1 ubiquitination: unique regulation of an essential survival protein. *Cells* **3** (2014) 418–437. doi:10.3390/cells3020418.

Opferman JT, Iwasaki H, Ong CC, Suh H, Mizuno Si, Akashi K, et al. Obligate role of anti-apoptotic MCL-1 in the survival of hematopoietic stem cells. *Science* **307** (2005) 1101–1104. doi:10.1126/science.1106114.

Qian H, Buza-Vidas N, Hyland CD, Jensen CT, Antonchuk J, Månsson R, et al. Critical role of thrombopoietin in maintaining adult quiescent hematopoietic stem cells. *Cell Stem Cell* **1** (2007) 671–684. doi:10.1016/j.stem.2007.10.008.

Varnum-Finney B, Xu L, Brashem-Stein C, Nourigat C, Flowers D, Bakkour S, et al. Pluripotent, cytokine-dependent, hematopoietic stem cells are immortalized by constitutive Notch1 signaling. *Nature Medicine* **6** (2000) 1278–1281. doi:10.1038/81390.

Wang MW, Consoli U, Lane CM, Durett A, Lauppe MJ, Champlin R, et al. Rescue from apoptosis in early (CD34-selected) versus late (non-CD34-selected) human hematopoietic cells by very late antigen 4- and vascular cell adhesion molecule (VCAM) 1-dependent adhesion to bone marrow stromal cells. *Cell growth & Differentiation* **9** (1998) 105–112.

Wang R, Xia L, Gabrilove J, Waxman S, Jing Y. Downregulation of Mcl-1 through GSK-3β activation contributes to arsenic trioxide-induced apoptosis in acute myeloid leukemia cells. *Leukemia* **27** (2013) 315–324. doi:10.1038/leu.2012.180.

Yoshimoto G, Miyamoto T, Jabbarzadeh-Tabrizi S, Iino T, Rocnik JL, Kikushige Y, et al. FLT3-ITD up-regulates MCL-1 to promote survival of stem cells in acute myeloid leukemia via FLT3-ITD-specific STAT5 activation. *Blood* **114** (2009) 5034–5043. doi:10.1182/blood-2008-12-196055.

Domen J. The role of apoptosis in regulating hematopoiesis and hematopoietic stem cells. *Immunologic Research* **22** (2000) 83–94. doi:10.1385/IR:22:2-3:83.

Oguro H, Iwama A. Life and death in hematopoietic stem cells. *Current Opinion in Immunology* **19** (2007) 503–509. doi:10.1016/j.coi.2007.05.001.

Orelio C, Dzierzak E. Bcl-2 expression and apoptosis in the regulation of hematopoietic stem cells. *Leukemia & Lymphoma* **48** (2007) 16–24. doi:10.1080/10428190601032529.

Shao L, Li H, Pazhanisamy SK, Meng A, Wang Y, Zhou D. Reactive oxygen species and hematopoietic stem cell senescence. *International Journal of Hematology* **94** (2011) 24–32. doi:10.1007/s12185-011-0872-1.

Ludin A, Gur-Cohen S, Golan K, Kaufmann KB, Itkin T, Medaglia C, et al. Reactive oxygen species regulate hematopoietic stem cell self-renewal, migration and development, as well as their bone marrow microenvironment. *Antioxidants & redox signaling* **21** (2014b) 1605–1619. doi:10.1089/ars.2014.5941.

Chen C, Liu Y, Liu Y, Zheng P. The axis of mTOR-mitochondria-ROS and stemness of the hematopoietic stem cells. *Cell cycle (Georgetown, Tex.)* **8** (2009) 1158–1160. doi:10.4161/cc.8.8.8139.

Jang YY, Sharkis SJ. A low level of reactive oxygen species selects for primitive hematopoietic stem cells that may reside in the low-oxygenic niche. *Blood* **110** (2007) 3056–3063. doi:10.1182/blood-2007-05-087759.

Cabezas-Wallscheid N, Buettner F, Sommerkamp P, Klimmeck D, Ladel L, Thalheimer FB, et al. Vitamin a-retinoic acid signaling regulates hematopoietic stem cell dormancy. *Cell* **169** (2017) 807–823. doi:https://doi.org/10.1016/j.cell.2017.04.018.

Baumgartner C, Toifl S, Farlik M, Halbritter F, Scheicher R, Fischer I, et al. An erk-dependent feedback mechanism prevents hematopoietic stem cell exhaustion. *Cell Stem Cell* **22** (2018) 879–892. doi:https://doi.org/10.1016/j.stem.2018.05.003.

Rodgers JT, King KY, Brett JO, Cromie MJ, Charville GW, Maguire KK, et al. mtorc1 controls the adaptive transition of quiescent stem cells from g 0 to g alert. *Nature* **510** (2014) 393–396. doi:https://doi.org/10.1038/nature13255.

Suda T, Takubo K, Semenza GL. Metabolic regulation of hematopoietic stem cells in the hypoxic niche. *Cell stem cell* **9** (2011b) 298–310. doi:10.1016/j.stem.2011.09.010.

Wilson A, Murphy MJ, Oskarsson T, Kaloulis K, Bettess MD, Oser GM, et al. c-Myc controls the balance between hematopoietic stem cell self-renewal and differentiation. *Genes & development* **18** (2004b) 2747–2763. doi:10.1101/gad.313104.

Forsberg EC, Prohaska SS, Katzman S, Heffner GC, Stuart JM, Weissman IL. Differential expression of novel potential regulators in hematopoietic stem cells. *PLoS genetics* **1** (2005) e28. doi:10.1371/journal.pgen.0010028.

Pant V, Quintás-Cardama A, Lozano G. The p53 pathway in hematopoiesis: lessons from mouse models, implications for humans. *Blood* **120** (2012b) 5118–5127. doi:10.1182/blood-2012-05-356014.

Matsumoto A, Takeishi S, Kanie T, Susaki E, Onoyama I, Tateishi Y, et al. p57 is required for quiescence and maintenance of adult hematopoietic stem cells. *Cell stem cell* **9** (2011b) 262–271. doi:10.1016/j.stem.2011.06.014.

Zou P, Yoshihara H, Hosokawa K, Tai I, Shinmyozu K, Tsukahara F, et al. p57(Kip2) and p27(Kip1) cooperate to maintain hematopoietic stem cell quiescence through interactions with Hsc70. *Cell stem cell* **9** (2011b) 247–261. doi:10.1016/j.stem.2011.07.003.

Tesio M, Trumpp A. Breaking the Cell Cycle of HSCs by p57 and Friends. *Cell stem cell* **9** (2011b) 187–192. doi:10.1016/j.stem.2011.08.005.

Umemoto T, Yamato M, Nishida K, Yang J, Tano Y, Okano T. p57Kip2 is expressed in quiescent mouse bone marrow side population cells. *Biochemical and Biophysical Research Communications* **337** (2005) 14–21. doi:10.1016/j.bbrc.2005.09.008.

Chabanon A, Desterke C, Rodenburger E, Clay D, Guerton B, Boutin L, et al. A cross-talk between stromal cell-derived factor-1 and transforming growth factor-β controls the quiescence/cycling switch of cd34+ progenitors through foxo3 and mammalian target of rapamycin. *Stem Cells* **26** (2008) 3150–3161. doi:10.1634/stemcells.2008-0219.

Pietras EM, Warr MR, Passegué E. Cell cycle regulation in hematopoietic stem cells. *The Journal of Cell Biology* **195** (2011b) 709–720. doi:10.1083/jcb.201102131.

Orford KW, Scadden DT. Deconstructing stem cell self-renewal: genetic insights into cell-cycle regulation. *Nature Reviews Genetics* **9** (2008) 115–128. doi:10.1038/nrg2269.

Fauré A, Naldi A, Chaouiya C, Thieffry D. Dynamical analysis of a generic boolean model for the control of the mammalian cell cycle. *Bioinformatics* **22** (2006) e124–e131. doi:https://doi.org/10.1093/bioinformatics/btl210.
